# Supplementary material for: The gap in contraceptive knowledge and use between the military and non-military populations of Kinshasa, DRC, 2016–2019
Source: PLoS One. 2021 Jul 27;16(7):e0254915. doi: 10.1371/journal.pone.0254915 (PMC8315532; doi:10.1371/journal.pone.0254915)
Supplement: S1 File — (DOCX) [file pone.0254915.s001.docx]

**S1 File. 2016 military survey questionnaire, English**

| **Female Questionnaire** |
| --- |

| **NO** | **QUESTIONS AND FILTERS** | **CODING CATEGORIES** | **SKIP** |
| --- | --- | --- | --- |
| **IDENTIFICATION** | | | |
| A | **Are you in the correct household?**  **This is the picture of the front of the home taken during the Household Questionnaire.**  **IF NOT, RETURN TO INTERVIEW THE CORRECT HOUSEHOLD.**  [ODK will display the photo attached to the linked Household Questionnaire] | Yes 1  No 0 |  |
| B | **Your name:** [Interviewer name from **Female Questionnaire**]  **Is this your name?** | Yes 1  No 0 |  |
|  | **Enter your name below.**  *Please record your name* | Interviewer’s Name |  |
| C | **Current date and time.** [ODK will display on screen]  **Is this date and time correct?** | Yes 1  No 0 | Skip to E if Yes |
| D | **Record the correct date and time.** | \| **Day** \| **Month** \| **Year** \| \| --- \| --- \| --- \| \|  \|  \|  \| \| **Hours** \| **Min** \| **AM/PM** \| \|  \|  \|  \| |  |
| E | **The following information is from the Female Questionnaire. Please review to make sure you are interviewing the correct respondent.**  [ODK will display the province, city, commune, and quartier for Kinshasa EAs and the province, district, *aire de santé*, and village for Kongo Central EAs. In addition, the Enumeration Area, Structure Number, and Household Number entered into the Household Questionnaire linked to this Female Questionnaire will be displayed.]  **Is the above information correct?** | Yes 1  No 0 |  |
|  | **CHECK: You should be attempting to interview [Respondent’s Name]. Is that correct?**  *If misspelled, select “yes” here and update the name in question “L.”*  *If this is the wrong person, you have two options:*  *(1) exit and ignore changes to this form. Open the correct form.*  *Or*  *(2) find and interview the person whose name appears above.* | Yes 1  No 0 |  |
| F | **Is the respondent present and available to be interviewed today?** | Yes 1  No 0 | Skip to K if No |
| G | **How well acquainted are you with the respondent?** | Very well acquainted 1  Well acquainted 2  Not well acquainted 3  Not acquainted 4 |  |
| G2 | **Has the respondent participated in a PMA2020 survey before?** | Yes 1  No 0  Don’t know -88  No response -99 |  |

|  |  |  |  |
| --- | --- | --- | --- |

| **INFORMED CONSENT**  *Find the woman between the ages of 15-49 associated with this Female Follow Up Questionnaire. The interview must have auditory privacy. Read the following greeting:* | | | | | | | | | | | | | |
| --- | --- | --- | --- | --- | --- | --- | --- | --- | --- | --- | --- | --- | --- |
| Hello. My name is ____________________________________ and I am working for the Kinshasa School of Public Health in collaboration with the Ministry of Health. We are conducting a survey in Kinshasa and Kongo Central that asks women about various reproductive health issues. We would very much appreciate your participation in this survey. This information will help us inform the government to better plan health services. The survey usually takes between 15 and 20 minutes to complete. Whatever information you provide will be kept strictly confidential and will not be shown to anyone other than members of our survey team.  Participation in this survey is voluntary, and if we should come to any question you don't want to answer, just let me know and I will go on to the next question; or you can stop the interview at any time. However, we hope that you will participate in this survey since your views are important.  At this time, do you want to ask me anything about the survey? | | | | | | | | | | | | | |
| H | | | Provide a paper copy of the Consent Form to the respondent and explain it. Then, ask:  **May I begin the interview now?** | | Yes 1  No 0 | | | | | | | Skip to K if No | |
|  | | | **Respondent’s signature**  *Please ask the respondent to sign or check the box in agreement of their participation.* | | GATHER SIGNATURE:  Check box: ☐ | | | | | | |  | |
| I | | | **Interviewer’s name:** [Interviewer name from Household Questionnaire]  *Mark your name as a witness to the consent process.* | | \|  \| \| --- \| | | | | | | |  | |
| J | | | **Respondent’s name**  [ODK will display the Respondent’s name from linked Household Roster]  *You may correct the spelling here if it is not correct, but you must be interviewing the person whose name appears below.* | | \|  \| \| --- \| | | | | | | |  | |
| **Section 1 – Respondent’s Background, Marital Status, HH characteristics**  *Now I would like to ask about your background and socioeconomic conditions.* | | | | | | | | | | | | | |
| 0 | | **In what month and year were you born?**  **The age in the household roster is [AGE].** | | | \| Month \|  \| \| --- \| --- \| \| Year \|  \| | | | | | | |  | |
| 1 | | **How old were you at your last birthday?**  *Must be more than 14. Must agree with FQ0.* | | | \| Age \|  \| \| --- \| --- \| | | | | | | |  | |
| 2 | | **What is the highest level of school you attended?** | | | Never Attended 0  Primary 1  Secondary 2  Tertiary………………………………. 3  No response -99 | | | | | | |  | |
| 3 | | **Are you currently married or living together with a man as if married?**  *Probe: If no, ask whether the respondent is divorced, separated, or widowed.* | | | No, never in union 0  Yes, currently married 1  Yes, living with a man 2  Not currently in union:  Divorced / separated 3  Not currently in union: Widow 4  No response -99 | | | | | | | Skip to 8 if No, never in union | |
| 4 | | **Have you been married or lived with a man only once or more than once?** | | | Only once 1  More than once 2  No response -99 | | | | | | | Skip to 5b if Only once | |
| 5a | | **In what month and year did you start living with your FIRST husband / partner?**  *Enter Jan 2020 for no response.* | | | \| Month \|  \| \| --- \| --- \| \| Year \|  \| | | | | | | |  | |
|  | | [If ≤15 years old at marriage date ODK will display:]  **CHECK: Based on the response you entered in FQ5a, the respondent was possibly 15 years old or younger at the time of her first marriage. Did you enter FQ5a correctly?** | | | Yes 1  No 0 | | | | | | |  | |
| 5b | | **Now I would like to ask about when you started living with your CURRENT or MOST RECENT husband / partner. In what month and year was that?**  *Enter Jan 2020 for no response.* | | | \| Month \|  \| \| --- \| --- \| \| Year \|  \| | | | | | | |  | |
|  | | [If ≤15 years old at marriage date ODK will display:]  **CHECK: Based on the response you entered in FQ5b, the respondent was possibly 15 years old or younger at the time of her first marriage. Did you enter FQ5b correctly?** | | | Yes 1  No 0 | | | | | | |  | |
|  | | **CHECK 3:** Currently married/cohabitating? | | | Yes 1  No 0 | | | | | | | Skip to 8 if No | |
| 6 | | **Does your husband / partner have other wives or does he live with other women as if married?** | | | Yes 1  No 0  Don’t know -88  No response -99 | | | | | | |  | |
| 7 | | **Is your husband / partner living with you now or is he staying elsewhere?** | | | Living with respondent 1  Staying elsewhere 2  No response -99 | | | | | | |  | |
| **Section 2 – Reproduction, Pregnancy & Fertility Preferences**  *Now I would like to ask about all the births you have had during your life.* | | | | | | | | | | | | | |
| 8a | | **How many times have you given birth?**  *Enter -99 for no response. 0 is a possible answer.* | | | \| Number \|  \| \| --- \| --- \| | | | | | | | Skip to 13 if 0 | |
|  | | **Were all of those live births?**  *If no, go back and change FQ8 to record only live birth events.* | | | Yes 1  No 0 | | | | | | | Skip to 9 if 8 was 1 | |
| 8b | | **How many sons and daughters have you given birth to and who were born alive?** | | | \| Number \|  \| \| --- \| --- \| | | | | | | |  | |
| 8c | | **Have you ever given birth to a boy or girl who was born alive but later died?**  *IF NO, PROBE: Any baby who cried or showed signs of life but did not survive?* | | | Yes 1  No 0 | | | | | | | Skip to 8e if No | |
| 8d | | **How many have died?**  *Enter -88 for do not know and -99 for No response.*  *Change FQ8c to 'No' if zero deaths.* | | | \| Number \|  \| \| --- \| --- \| | | | | | | |  | |
|  | | **READ THIS CHECK OUT LOUD: Just to make sure I have this right: you had a total of ___ birth(s) during your life, resulting in ____ son(s) or daughter(s) born alive.**  **Is that correct?** | | | Yes 1  No 0 | | | | | | | If no, go back and probe to correct 8a-c. | |
| 8e | | **When was your FIRST birth?**  *Please record the date of the first live birth. Date should be found by calculating forward or backward from memorable events if needed. Enter Jan 2020 for no response.* | | | \| Month \|  \| \| --- \| --- \| \| Year \|  \| | | | | | | |  | |
| 9 | | **When was your MOST RECENT birth?**  *Please record the date of the MOST RECENT live birth. The date should be found by calculating backwards from memorable events if needed.*  *Enter Jan 2020 for no response.* | | | \| Month \|  \| \| --- \| --- \| \| Year \|  \| | | | | | | | Skip to 11 if not in last year and/or Q8 is 1 | |
| 10 | | **When did you give birth before the most recent one?**  *Please record the date of the birth before the last. The date should be found by calculating backwards from memorable events if needed.*  *Enter Jan 2020 for no response.* | | | \| Month \|  \| \| --- \| --- \| \| Year \|  \| | | | | | | |  | |
| 11 | | **Is your last baby / child still alive?** | | | Yes 1  No 0  Don’t know -88  No response -99 | | | | | | | Skip to 13 if Yes | |
| 12 | | **When did your last baby / child die?**  *Please record the date of the child’s death.*  *The date should be found by calculating backwards from memorable events if needed.*  *Enter Jan 2020 for no response.* | | | \| Month \|  \| \| --- \| --- \| \| Year \|  \| | | | | | | |  | |
| 13 | | **When did your last menstrual period start?**  *If you select days, weeks, months or years, you will enter a number for x on the next screen.*  *Enter 0 days for today, not 0 weeks/months/years.* | | | ______ days ago | | | | | | |  | |
|  |  |  |  |  | ______ weeks ago | | | | | | |  |  |
|  |  |  |  |  | _______ months ago | | | | | | |  |  |
|  |  |  |  |  | _______ years ago | | | | | | |  |  |
|  |  |  |  |  | Menopausal / Hysterectomy 5  Before last birth 6  Never menstruated 7  No response -99 | | | | | | |  |  |
| 14 | | **Are you pregnant now?** | | | Yes 1  No 0  Unsure 2  No response -99 | | | | | | | Skip to 16 if No or Unsure | |
| 15 | | **How many months pregnant are you?**  **The most recent birth was: [Date of most recent birth]**  *Please record the number of completed months. Enter -88 for do not know, -99 for no response.* | | | \| Number of months \|  \| \| --- \| --- \| | | | | | | |  | |
|  | | **CHECK 14:** Currently pregnant? | | | Yes 1  No 0 | | | | | | | 16a if no  16b if yes | |
| 16a | | **Now I have some questions about the future. Would you like to have a/another child or would you prefer not to have any / any more children?** | | | Have a/another child 1  No more 2  Says she can’t get pregnant 3  Undecided / Don’t know -88  No response -99 | | | | | | | Skip to 17a if 1 and 18 for all other | |
| 16b | | **Now I have some questions about the future.**  **After the child you are expecting now, would you like to have another child, or would you prefer not to have any more children?** | | | Have a/another child 1  No more/prefer no children 2  Says she can’t get pregnant 3  Undecided / Don’t know -88  No response -99 | | | | | | | Skip to 17b if 1 and 18 for all other | |
| 17a | | **How long would you like to wait from now before the birth of a/another child?**  *If you select months or years, you will enter a number for x on the next screen.*  *Select “Years” if more than 36 months.* | | | \| Months \|  \| \| --- \| --- \| \|  \|  \| \| Years \|  \| | | | | | | |  | |
|  |  |  |  |  | Soon / now 1  Says she can’t get pregnant 2  Other 3  Don’t know -88  No response -99 | | | | | | |  |  |
| 17b | | **After the birth of the child you are expecting now, how long would you like to wait before the birth of another child?**  *If you select months or years, you will enter a number for x on the next screen.*  *Select “Years” if more than 36 months.* | | | \| Months \|  \| \| --- \| --- \| \|  \|  \| \| Years \|  \| | | | | | | |  | |
|  |  |  |  |  | Soon / now 1  Says she can’t get pregnant 2  Other 3  Don’t know -88  No response -99 | | | | | | |  |  |
|  | | **CHECK 8:** Number of births  **CHECK 14**: Currently pregnant? | | | \| Number of births \|  \| \| --- \| --- \| | | | | | | | Skip to 19 if 0 births and 14: No.  Skip to 18a if 14: no and 18b if 14: yes | |
|  |  |  |  |  | Yes………………………………… 1  No………………………………….0 | | | | | | |  |  |
| 18a | | **Now I would like to ask a question about your last birth.**  **At the time you became pregnant, did you want to become pregnant then, did you want to wait until later, or did you not want to have any / any more children at all?** | | | Then 1  Later 2  Not at all 3  No response -99 | | | | | | |  | |
| 18b | | **Now I would like to ask a question about your current pregnancy.**  **At the time you became pregnant, did you want to become pregnant then, did you want to wait until later, or did you not want to have any / any more children at all?** | | | Then 1  Later 2  Not at all 3  No response -99 | | | | | | |  | |
| **Section 3A – Contraception**  *Now I would like to talk about family planning - the various ways or methods that a couple can use to delay or avoid a pregnancy.*  *An image will appear on the screen for some methods. If the respondent says that she has not heard of the method or if she hesitates to answer, read the probe aloud and show her the image, if available.* | | | | | | | | | | | | | |
| 19 | **Have you ever heard of female sterilization?**  PROBE: Women can have an operation to avoid having any more children.  [NO IMAGE] | | | Yes 1  No 0  No response………………………..-99 | | | | | | |  | | |
| 19 | **Have you ever heard of male sterilization?**  PROBE: Men can have an operation to avoid having any more children.  [NO IMAGE] | | | Yes 1  No 0  No response………………………..-99 | | | | | | |  | | |
| 19 | **Have you ever heard of the contraceptive implant?**  PROBE: Women can have one or several small rods placed in their upper arm by a doctor or nurse, which can prevent pregnancy for one or more years.  [IMAGE OF METHOD WILL APPEAR ON SCREEN] | | | Yes 1  No 0  No response………………………..-99 | | | | | | |  | | |
| 19 | **Have you ever heard of the IUD?**  PROBE: Women can have a loop or coil placed inside them by a doctor or a nurse.  [IMAGE OF METHOD WILL APPEAR ON SCREEN] | | | Yes 1  No 0  No response………………………..-99 | | | | | | |  | | |
| 19 | **Have you ever heard of injectables?**  PROBE: Women can have an injection by a health provider that stops them from becoming pregnant for one or more months.  [IMAGE OF SAYANA PRESS AND DEPO PROVERA WILL APPEAR ON SCREEN] | | | Yes 1  No 0  No response………………………..-99 | | | | | | |  | | |
| 19 | **Have you ever heard of the (birth control) pill?**  PROBE: Women can take a pill every day to avoid becoming pregnant.  [IMAGE OF METHOD WILL APPEAR ON SCREEN] | | | Yes 1  No 0  No response………………………..-99 | | | | | | |  | | |
| 19 | **Have you ever heard of emergency contraception?**  PROBE: As an emergency measure after unprotected sexual intercourse women can take special pills at any time within five days to prevent pregnancy.  [NO IMAGE] | | | Yes 1  No 0  No response………………………..-99 | | | | | | |  | | |
| 19 | **Have you ever heard of condoms?**  PROBE: Men can put a rubber sheath on their penis before sexual intercourse.  [IMAGE OF METHOD WILL APPEAR ON SCREEN] | | | Yes 1  No 0  No response………………………..-99 | | | | | | |  | | |
| 19 | **Have you ever heard of female condoms?**  PROBE: Women can put a sheath in their vagina before sexual intercourse.  [IMAGE OF METHOD WILL APPEAR ON SCREEN] | | | Yes 1  No 0  No response………………………..-99 | | | | | | |  | | |
| 19 | **Have you ever heard of the diaphragm?**  PROBE: Women can place a thin flexible disk in their vagina before sexual intercourse.  [IMAGE OF METHOD WILL APPEAR ON SCREEN] | | | Yes 1  No 0  No response………………………..-99 | | | | | | |  | | |
| 19 | **Have you ever heard of foam or jelly as a contraceptive method?**  PROBE: Women can place a suppository, jelly, or cream in their vagina before sexual intercourse to prevent pregnancy.  [IMAGE OF METHOD WILL APPEAR ON SCREEN] | | | Yes 1  No 0  No response………………………..-99 | | | | | | |  | | |
| 19 | **Have you ever heard of the standard days method or Cycle Beads?**  PROBE: A Woman can use a string of colored beads to know the days she can get pregnant. On the days she can get pregnant, she and her partner use a condom or do not have sexual intercourse.  [IMAGE OF METHOD WILL APPEAR ON SCREEN] | | | Yes 1  No 0  No response………………………-99 | | | | | | |  | | |
| 19 | **Have you ever heard of the Lactational Amenorrhea Method or LAM?**  [NO DESCRIPTION; NO IMAGE] | | | Yes 1  No 0  No response………………………..-99 | | | | | | |  | | |
| 19 | **Have you ever heard of the rhythm method?**  PROBE: Women can avoid pregnancy by not having sexual intercourse on the days of the month they think they can get pregnant.  [NO IMAGE] | | | Yes 1  No 0  No response………………………..-99 | | | | | | |  | | |
| 19 | **Have you ever heard of the withdrawal method?**  PROBE: Men can be careful and pull out before climax.  [NO IMAGE] | | | Yes 1  No 0  No response………………………..-99 | | | | | | |  | | |
| 19 | **Have you ever heard of any other ways or methods that women or men can use to avoid pregnancy?** | | | Yes 1  No 0  No response………………………..-99 | | | | | | |  | | |
|  | **CHECK 14:** Currently pregnant? | | | Yes 1  No 0 | | | | | | | Skip to 23 if yes | | |
| 20 | **Are you or your partner currently doing something or using any method to delay or avoid getting pregnant?** | | | Yes 1  No 0 | | | | | | | Skip to 23 if No | | |
| 21 | **Which method or methods are you using?**  **Probe: Anything else?**  *Select all methods mentioned. Be sure to scroll to bottom to see all choices.* | | | Female sterilization  Male sterilization  Implant  IUD  Injectable  Pill  Emergency Contraception  Male Condom  Female Condom  Diaphragm  Foam/Jelly  Std. Days/Cycle beads  LAM  Rhythm method  Withdrawal  Other traditional method  No response -99 | | | | | 1  2  3  4  5  7  8  9  10  11  12  13  14  30  31  39  -99 | | Skip based on most effective method only  If injectable is selected, skip to FQ21a  If LAM is selected but not injectables, skip to 21b  If FS or MS was selected without injectables or LAM, skip to 22  If LAM, FS, MS, and injectables are not selected, skip to 26b | | |
| 21a | **PROBE:** Was the injection administered via syringe or small needle?  *Show the image to the respondent.*  [IMAGES OF BOTH INJECTION SYSTEMS WILL APPEAR ON SCREEN] | | | Syringe 1  Small needle (Sayana Press) 2  Both 3  No response………………………..-99 | | | | | | | Skip to CHECK FQ21 | | |
| 21b | **Are you breastfeeding to delay or avoid becoming pregnant?** | | | Yes 1  No 0  No Response……………………… -99 | | | | | | |  | | |
|  | **CHECK FQ21:** Using Female Sterilization and/or Male Sterilization? | | | Female Sterilization  Male Sterilization  None of the above | | | | Y  1  1  -77 | | N  0  0 | Skip to 26b if -77 | | |
| 22 | **Did the provider tell you or your partner that this method was permanent?** | | | Yes 1  No 0  No response………………………..-99 | | | | | | | Skip to 26b | | |
| 23 | **Do you know of a place where you can obtain a method of family planning?** | | | Yes 1  No 0  No response………………………..-99 | | | | | | |  | | |
|  | **CHECK 14:** Currently pregnant? | | | Yes 1  No 0 | | | | | | | Skip to  24b if yes | | |
| 24a | **You said that you are not currently using a contraceptive method. Do you think you will use a contraceptive method to delay or avoid getting pregnant at any time in the future?** | | | Yes 1  No 0  No response………………………..-99 | | | | | | |  | | |
| 24b | **Do you think you will use a contraceptive method to delay or avoid getting pregnant at any time in the future?** | | | Yes 1  No 0  No response………………………..-99 | | | | | | |  | | |
| 25 | **In the last 12 months, have you ever done something or used a method to delay or avoid getting pregnant?** | | | Yes 1  No 0  No response………………………..-99 | | | | | | | Skip to 41 if No | | |
| 26 | **Which method did you use most recently?**  **Probe: Anything else?**  *Select most effective method (highest method on list). Scroll to bottom to see all choices.* | | | Implant  IUD  Injectable  Pill  Emergency Contraception  Male Condom  Female Condom  Diaphragm  Foam/Jelly  Std. Days/Cycle beads  LAM  Rhythm method  Withdrawal  Other traditional method  No response | | | | | | 3  4  5  7  8  9  10  11  12  13  14  30  31  39  -99 | Skip to FQ26b unless injectables selected | | |
| 26a | **PROBE:** Was the injection administered via syringe or small needle?  *Show the image to the respondent.*  [IMAGES OF BOTH INJECTION SYSTEMS WILL APPEAR ON SCREEN] | | | Syringe 1  Small needle (Sayana Press) 2  Both 3  No response………………………..-99 | | | | | | |  | | |
| 26b | **Before you started using [MOST RECENT / CURRENT METHOD], did you talk with your husband / partner about using a contraceptive method?** | | | Yes 1  No 0  Don’t know -88  No response………………………..-99 | | | | | | |  | | |
| 27 | **When did you begin using your [MOST RECENT / CURRENT METHOD]?**  *Calculate backwards from memorable events if needed.*  **Age at first use: [Age from FQ20]**  **Most Recent Birth: [mm-yyyy]**  **Current Marriage: [mm-yyyy]**  *Must be at least the ages she started using a contraceptive method (FQ20).*  *Must be before today. Respondent must be at least 10 years old.*  *Enter Jan 2020 for no response.* | | | \| Month \|  \| \| --- \| --- \| \| Year \|  \| | | | | | | |  | | |
|  | **CHECK 20:** Currently using contraceptives? | | | Yes 1  No 0 | | | | | | | Skip to 30 if Yes | | |
| 28 | **When did you stop using your [MOST RECENT METHOD]?**  *Please record the date.*  *The date should be found by calculating backwards from memorable events if needed. Must be after FQ27.*  *Enter Jan 2020 for no response.* | | | \| Month \|  \| \| --- \| --- \| \| Year \|  \| | | | | | | |  | | |
| 29 | **Why did you stop using your (MOST RECENT METHOD)?** | | | Infrequent sex / husband away 1  Became pregnant while using 2  Wanted to become pregnant 3  Husband / partner disapproved 4  Wanted more effective method 5  No method available 6  Health concerns 7  Fear of side effects 8  Lack of access / too far 9  Costs too much 10  Inconvenient to use 11  Fatalistic 12  Difficult to get pregnant /  menopausal 13  Interferes with body’s processes 14  Other 15  Don’t know -88  No response -99 | | | | | | |  | | |
| 30 | **You first started using [CURRENT/MOST RECENT METHOD] in [DATE FROM FQ27]. Where did you get it at that time?**  *Scroll to bottom to see all choices.* | | | **PUBLIC SECTOR:**  **NATIONAL HOSPITAL………………………………………11**  **FAMILY PLANNING CLINIC……………………….…. 12**  **HEALTH CENTRE/POSTE DE SANTE……………….13**  **MATERNITY.………………………………………………..14**  **COMMUNITY HEALTH VOLUNTEER………………15**  **REGIONAL HOSPITAL………………………………..….16**  **PRIVATE MEDICAL SECTOR:**  **PRIVATE HOSPITAL/CLINIC…………………………. 21**  **PHARMACY …………………………………………………22**  **NGO………………………………………………………….…23**  **PRIVATE HEALTH CENTER……………………………24**  **PRIVATE PRACTICE ………………………………………25**  **PRIVATE DOCTOR ……………………………………….26**  **MOBILE NURSE………………………………………….…27**  **Community health worker (ASC)……………………28**  **Community-based medical student………………………………………….…29**  **OTHER SOURCE:**  **BOUTIQUE…………………………………………………….31**  **RELIGIOUS INSTITUTION………………………………32**  **FRIEND/RELATIVE ……………………………………...33**  **BAR/NIGHT CLUB………………………………………….34**  **LIGABLO/KIOSK……………………………………………35**  **CHAYEUR………………………………………..…………..36**  **OTHER ………………………………………………………. 37**  **NO RESPONSE…………………………………………… -99** | | | | | | |  | |  |
| 31 | **When you obtained your [MOST RECENT / CURRENT METHOD], were you told by the provider about side effects or problems you might have with a method to delay or avoid getting pregnant?** | | | Yes 1  No 0  No response -99 | | | | | | | Skip to 33 if No | | |
| 32 | **Were you told what to do if you experienced side effects or problems?** | | | Yes 1  No 0  No response -99 | | | | | | |  | | |
| 33 | **At that time, were you told by the family planning provider about methods of family planning other than the [MOST RECENT/CURRENT METHOD] that you could use?** | | | Yes 1  No 0  No response -99 | | | | | | |  | | |
| 34 | **During that visit, did you obtain the method you wanted to delay or avoid getting pregnant?** | | | Yes 1  No 0  No response -99 | | | | | | | Skip to 36 if yes | | |
| 35 | **Why didn’t you obtain the method you wanted?** | | | Method out of stock that day 1  Method not available at all 2  Provider not trained to provide the method 3  Provider recommended a different method 4  Not eligible for method 5  Decided not to adopt a method 6  Too costly 7  Other 8  No response -99 | | | | | | |  | | |
| 36 | **During that visit, who made the final decision about what method you got?** | | | You alone 1  Provider 2  Partner 3  You and provider 4  You and partner 5  Other 6  No response -99 | | | | | | |  | | |
|  | **CHECK 30:**  **You first started using [CURRENT/MOST RECENT METHOD] in [DATE FROM FQ27]. Where did you get it at that time?** | | | **PUBLIC SECTOR:**  **NATIONAL HOSPITAL……………………………………11**  **FAMILY PLANNING CLINIC……………………….…. 12**  **HEALTH CENTRE/POSTE DE SANTE……………….13**  **MATERNITY.………………………………………………..14**  **COMMUNITY HEALTH VOLUNTEER………………15**  **REGIONAL HOSPITAL………………………………..….16**  **PRIVATE MEDICAL SECTOR:**  **PRIVATE HOSPITAL/CLINIC…………………………. 21**  **PHARMACY …………………………………………………22**  **NGO……………………………………………………….……23**  **PRIVATE HEALTH CENTER………………………….…24**  **PRIVATE PRACTICE ………………………………………25**  **PRIVATE DOCTOR ……………………………….……….26**  **MOBILE NURSE………………………………………….…27**  **Community health worker (ASC) ..………………28**  **Community-based medical student………………………………………….…29**  **OTHER SOURCE:**  **BOUTIQUE…………………………………………..……….31**  **RELIGIOUS INSTITUTION………………………………32**  **FRIEND/RELATIVE …………………………………..…..33**  **BAR/NIGHT CLUB…………………………………..…….34**  **LIGABLO/KIOSK……………………………………………35**  **CHAYEUR……………………………………..……………..36**  **OTHER ………………………………………………………. 37**  **NO RESPONSE…………………………………………… -99** | | | | | | | Skip to 41 if 30 is 33 OR 37 | | |
| 37 | **Would you return to this provider?**  **Provider: [Type of Provider from FQ30]** | | | Yes 1  No 0  No response -99 | | | | | | |  | | |
| 38 | **Would you refer your relative or friend to this provider / facility?** | | | Yes 1  No 0  No response -99 | | | | | | |  | | |
| 39 | **In the last 12 months, have you paid any fees for family planning services (including the most current method)?** | | | Yes 1  No 0 | | | | | | | Skip to 41 if No | | |
| 40 | **How much did you pay?**  *Enter all prices in Congolese Francs. Enter -88 if respondent does not know, -99 for no response.* | | | \| Fee \|  \| \| --- \| --- \| | | | | | | |  | | |
| 40a | **Where did you obtain [CURRENT METHOD] the last time?**  ***Scroll to bottom to see all choices.*** | | | **PUBLIC SECTOR:**  **NATIONAL HOSPITAL………………………………………11**  **FAMILY PLANNING CLINIC………………………….…. 12**  **HEALTH CENTRE/POSTE DE SANTE………………….13**  **MATERNITE.…………………………………………………..14**  **COMMUNITY HEALTH VOLUNTEER…………………15**  **REGIONAL HOSPITAL…………………………………..….16**  **PRIVATE MEDICAL SECTOR:**  **PRIVATE HOSPITAL/CLINIC……………………………. 21**  **PHARMACY ……………………………………………………22**  **NGO………………………………………………………….……23**  **PRIVATE HEALTH CENTER…………………………….…24**  **PRIVATE PRACTICE …………………………………………25**  **PRIVATE DOCTOR ………………………………….……….26**  **MOBILE NURSE…………………………………………….…27**  **FIELDWORKER………………………..………………………28**  **Community health worker (ASC)..………..……28**  **Community-based medical student………………………………………….…29**  **OTHER SOURCE:**  **BOUTIQUE……………………………………………..……….31**  **RELIGIOUS INSTITUTION…………………………………32**  **FRIEND/RELATIVE ……………………………………..…..33**  **BAR/NIGHT CLUB……………………………………..…….34**  **LIGABLO/KIOSK………………………………………………35**  **CHAYEUR………………………………………..……………..36**  **OTHER …………………………………………………………. 37**  **NO RESPONSE……………………………………………… -99** | | | | | | |  | | |
| 41 | **Have you ever done anything or tried in any way to delay or avoid getting pregnant?** | | | | Yes 1  No 0  No response -99 | | | | | | Skip to 43 if No | | |
| 41b | **How old were you when you first used a method to delay or avoid getting pregnant?**  **The respondent said she was [age from FQ1] years old at her last birthday.**  *Enter the age in years.*  *Enter -88 if respondent does not know.*  *Enter -99 if there is no response.*  *Cannot be younger than 9.* | | | | \| Age \|  \| \| --- \| --- \| | | | | | |  | | |
| 41c | **How many living children did you have at that time, if any?**  **Note: the respondent said that she gave birth [number of live births] times in FQ8.**  *Enter -99 for no response* | | | | \| Number \|  \| \| --- \| --- \| | | | | | |  | | |
| 42 | **Which method did you first use to delay or avoid getting pregnant?**  *Do not read the method choices. Be sure to scroll to bottom to see all choices.* | | | | Female sterilization  Male sterilization  Implant  IUD  Injectable  Pill  Emergency Contraception  Male Condom  Female Condom  Diaphragm  Foam/Jelly  Std. Days/Cycle beads  LAM  Rhythm method  Withdrawal  Other traditional method  No response -99 | | | | | 1  2  3  4  5  7  8  9  10  11  12  13  14  30  31  39  -99 | If Injectables is selected, go to 42a | | |
| 42a | **PROBE:** Was the injection administered via syringe or small needle?  *Show the image to the respondent.*  [IMAGES OF BOTH INJECTION SYSTEMS WILL APPEAR ON SCREEN] | | | Syringe 1  Small needle (Sayana Press) 2  Both 3  No response………………………..-99 | | | | | | |  | | |
|  | **CHECK 16:** Desire for future child?  **CHECK 17**: 2 or more years before next child?  **CHECK 20:** Currently using contraceptive method? | | | | Have a/another child 1  No more/none 2  Says she can’t get pregnant 3  Undecided / Don’t know -88 | | | | | | Ask 43 to non-users (current or ever) who do not want a/another child or not before 2 years. | | |
|  |  |  |  |  | No more/none 1  Less than 2 years 2  2 or more years 3 | | | | | |  |  |  |
|  |  |  |  |  | Yes, using contraceptive 1  No, not using contraceptive 0 | | | | | |  |  |  |
| 43 | **You said that you do not want any / anymore children and that you are not using a method to avoid pregnancy.**  **Can you tell me the reason why you are not using a method to prevent pregnancy?**  **PROBE: Any other reason?**  *RECORD ALL REASONS MENTIONED.*  *Cannot select “Do Not Know” or “No response” with other options.*  *Cannot select “Not married” if FQ3 is “Yes, currently married”.*  *Scroll to the bottom to see all choices.* | | | | Not married 1  Infrequent sex / husband away 2  Menopausal/Hysterectomy 3  Subfecund / infecund 4  Not menstruated since last birth 5  Breastfeeding 6  Husband away for many days 7  Up to God / fatalistic 8  Respondent opposed 9  Husband / partner opposed 10  Others opposed 11  Religious prohibition 12  Knows no method 13  Knows no source 14  Fear of side effects 15  Health concerns 16  Lack of access / too far 17  Costs too much 18Preferred method not available 19  No method available 20  Inconvenient to use 21  Interferes with body’s processes 22  Takes too much time away from regular duties / too busy to go get one....…………23  Intention to use one but did not get a chance to go to the clinic yet…...…24  Mother-in-law opposed 25  Other 36  Don’t know -88  No response -99 | | | | | |  | | |
| 44 | **In the last 12 months, were you visited by a** **community health worker who talked to you about family planning?** | | | | Yes 1  No 0  No response -99 | | | | | |  | | |
| 44a | **In the last 12 months, did you participate in a group talk at the community level about family planning?** | | | Yes 1  No 0  No response -99 | | | | | | |  | | |
| 45 | **In the last 12 months, have you visited a health facility for care for yourself or your children?**  *For any health services* | | | Yes 1  No 0  No response -99 | | | | | | | Skip to 47 if no | | |
| 46 | **Did any staff member at the health facility speak to you about family planning methods?** | | | Yes 1  No 0  No response -99 | | | | | | |  | | |
| 47 | **In the last few months have you:**  **Heard about family planning on the radio?**  **Seen anything about family planning on the television?**  **Read about family planning in a newspaper or magazine?** | | | ………………… | | Yes  1  1  1 | No  0  0  0 | | NR  -99  -99  -99 | |  | | |
| 47b | **Have you seen this image before?**    ***Show the logo to the respondent.***  **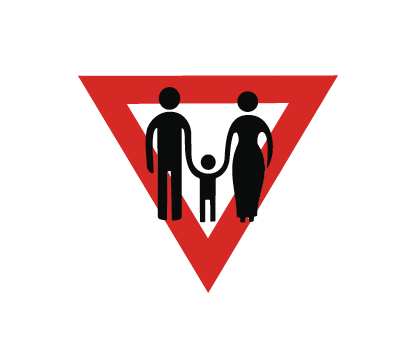** | | | Yes 1  No 0  Don’t know/Don’t recall -88  No response -99 | | | | | | | Skip to 47d if 0, -88, or -99 | | |
| 47b1 | **Where did you see this image?**  **PROBE: Anywhere else?**  *Select all that apply.* | | | In a health center 1  In the street 2  In a pharmacy 3  On a billboard ……………………….4  On the TV ……………………………5  On a leaflet ………………………….6  On a community heath agent’s vest …7  Other 8  Don’t know/Don’t recall -88  No response -99 | | | | | | |  | | |
| 47c | **What is this image trying to say?**  ***Select all that apply.*** | | | Family planning 1  Birth spacing 2  Reproductive health 3  The family unit 4  Other 5  Don’t know -88  No response -99 | | | | | | |  | | |
| 47d | **Have you seen the billboard that states “How many children do you want?”, and shows the FP logo ?**  **Show a photo of the billboard to the respondent.**  **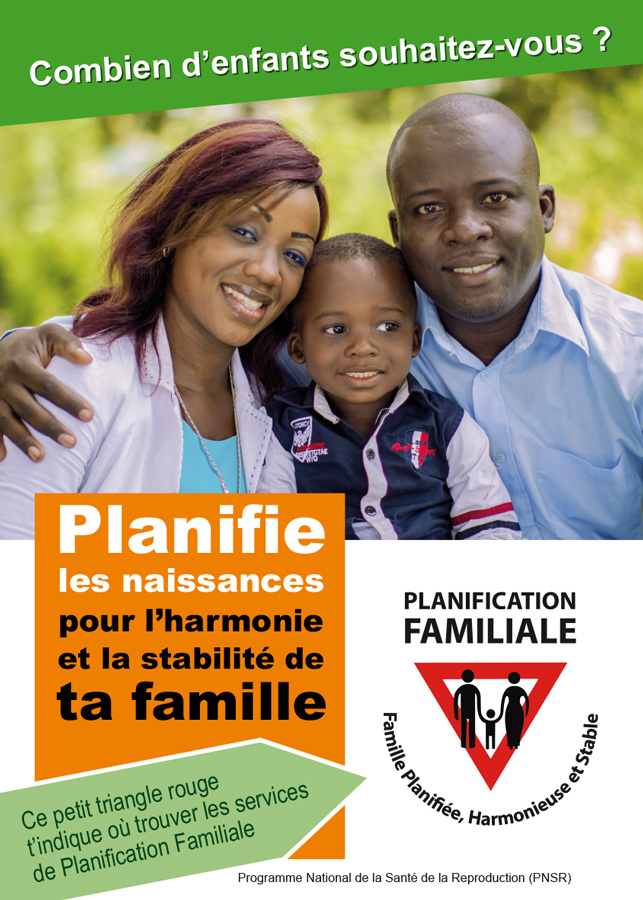** | | | Yes 1  No 0  Don’t know/Don’t recall -88  No response -99 | | | | | | | Skip to 48 if 0, -88 or -99 | | |
| 47e | **What is this image trying to say?**  ***Select all that apply.*** | | | Family planning 1  Birth spacing 2  Reproductive health 3  The family unit 4  Other 5  Don’t know / Don’t recall -88  No response -99 | | | | | | |  | | |
|  | **CHECK FOR THE PRESENCE OF OTHERS. BEFORE CONTINUING, MAKE EVERY EFFORT TO ENSURE PRIVACY.**  ***Verbally prepare the respondent for sexual activity questions.*** | | | | | | | | | |  | | |
| 48 | **How old were you when you first had sexual intercourse?**  ***The respondent said she was [age from FQ1] years old at her last birthday.***  ***[She has had x live births.]***  *Enter the age in years.*  *Enter -77 if she never had sex.*  *Enter -88 if respondent does not know.*  *Enter -99 for no response.* | | | | \| Age \|  \| \| --- \| --- \| | | | | | | Skip to MM_1 if -77 | | |
|  | [If age at first sex <10 years:]  **You have entered that the respondent was X years old when she first had sexual intercourse. Is this what she said?**  *Go back and correct FQ48 if it is not correct.* | | | | Yes 1  No 0 | | | | | |  | | |
| 49 | **When was the last time you had sexual intercourse?**  *If less than 12 months ago, answer must be recorded in months, weeks, or days.*  *Enter 0 days for today.*  *You will enter a number for X on the next screen.* | | | | ______ days ago  ______ weeks ago  _______ months ago  _______ years ago | | | | | |  | | |

| **Section 4 – Exposure to Mass Media**  *Now I would like to talk in more detail about your exposure to family planning information through mass media.* | | | |
| --- | --- | --- | --- |
| MM_1 | **Did you watch the television show** ‘**Libala Ya Bosembo’ within the past 6 months?** | Yes 1  No 0  No response -99 | Skip to MM_6 if 0 or -99 |
| MM_2 | At the end of each libala ya bosembo show a message is given. The last time you watched the show, what messages had the greatest impression on you? ?  **PROBE: What else?**  ***Select all that apply.*** | Couple communication………………1  Harmony in the family 2  Family planning methods 3  Antenatal care 4  Care for mother and child’s health in the postpartum period 5  Amenorrheic period after pregnancy 6  Birth spacing 7  Breast feeding 8  Other 9  Don’t know / Don’t recall -88  No response -99 |  |
| MM_3 | **Did they talk about using family planning methods during the show?** | Yes 1  No 0  Don’t know / Don’t recall -88  No response -99 | Skip to MM_6 if 0, -88 or -99 |
| MM_4 | **Did you speak with someone about the family planning messages from the television show ‘Libala Ya Bosembo?’** | Yes 1  No 0  Don’t know / Don’t recall -88  No response -99 | Skip to MM_6 if 0,  -88 or -99 |
| MM_5 | **With whom did you speak?**  **PROBE: Anyone else?**  ***Select all that apply.*** | Medical provider 1  Spouse 2  Other relatives 3  Friends/neighbors 4  Pharmacist/pharmacist’s aid 5  Outreach worker 6  People at a seminar/community meeting 7  Other 8  Don’t know / Don’t recall -88  No response -99 |  |
| MM_6 | **Have you ever watched the mini-television show called “Elengi”?** | Yes 1  No 0  No response -99 | Skip to MM_8 if 0 or -99 |
| MM_7 | **Last time you watched the mini-television show Elengi, what was it about?**  **PROBE: What else?**  ***Select all that apply.*** | Couple communication………………1  Harmony in the family 2  Family planning methods 3  Antenatal care 4  Care for mother and child’s health in the postpartum period 5  Amenorrheic period after pregnancy 6  Birth spacing 7  Breast feeding 8  Other 9  Don’t know / don’t recall -88  No response -99 |  |
| MM_8 | **Have you ever called the telephone hotline ‘3-2-1’?** | Yes 1  No 0  No response -99 | Skip to SE_1 if 0 or -99 |
| MM_9 | The last time you called the hotline 3-2-1, what did you want information about?  **PROBE: Anything else?**  ***Select all that apply.*** | Family planning service locations 1  Information about family planning methods 2  Side-effects of FP methods 3  Post-abortion care 4  Post intimate partner violence care 5  HIV/AIDS 6  Health in general 7  Other 8  Don’t know -88  No response -99 |  |

|  | | **Section 5 – Perceived self-efficacy in contraceptive use**  *Now I would like to talk about your level of confidence in your ability to access and use family planning methods. If you are not currently married or have a regular partner, try to imagine how you would respond if you were currently in union.* | | | | | |
| --- | --- | --- | --- | --- | --- | --- | --- |
|  | How confident are you that: | | *Very Confident* | *Confident* | *Somewhat confident* | *Not very confident* | *Not at all confident* |
| SE_1 | You can start a conversation with your husband/spouse/partner about family planning/birth spacing? | | 5 | 4 | 3 | 2 | 1 |
| SE_2 | You can convince your husband/spouse/partner to use a method of family planning/birth spacing? | | 5 | 4 | 3 | 2 | 1 |
| SE_3 | You can go to a site where methods of family planning / birth spacing are available if you decide to use one? | | 5 | 4 | 3 | 2 | 1 |
| SE_4 | You can get a method of family planning / birth spacing if you decide to use one? | | 5 | 4 | 3 | 2 | 1 |
| SE_5 | You could use a method of family planning / birth spacing, even if your husband/partner does not want to? | | 5 | 4 | 3 | 2 | 1 |
| SE_6 | You can use a method of family planning / birth spacing, even if none of your friends or neighbors use one? | | 5 | 4 | 3 | 2 | 1 |
| SE_7 | You can use a method of family / child spacing planning, even if your religious leader does not think you should use it? | | 5 | 4 | 3 | 2 | 1 |
| SE_8 | You will continue to use a method of family planning / birth spacing, even if you experience side effects? | | 5 | 4 | 3 | 2 | 1 |

| **Section 6 – Diarrheal Disease Among Children**  *Now I would like to ask about your water practices.* | | | | |
| --- | --- | --- | --- | --- |
| 50 | | **How many children under age 5, if any, live in this household for which you are the primary caregiver?** | \| Number \|  \| \| --- \| --- \| | |
|  | | Starting with the youngest child, I’d like to ask you some questions.  **[ODK Will repeat the FQ51-FQ53 each child under age 5.]** | | |
| 51 | | In what month and year was this child born?  **ENTER JAN 2020 FOR NO RESPONSE.** | \| Month \|  \| \| --- \| --- \| \| Year \|  \| | |
| 52 | | The last time this child passed stools, what was done to dispose of the stools?  **FOR ALL FECES, NORMAL OR DIARRHEA.**  Children use a latrine / toilet  Leave waste where it is  Bury waste in field / yard  Dispose of waste in latrine / toilet  Dispose of waste with rubbish / garbage  Dispose of waste with waste water  Use it as manure  Burn it  No response | Yes  1  1  1  1  1  1  1  1  1  -99 | No  0  0  0  0  0  0  0  0  0 |
| 53 | | In the past 7 days, has this child had diarrhea?  ***Diarrhea is determined as perceived by mother/ caretaker. If the respondent is not sure what we mean by diarrhea, tell her it means “three or more runny stools per day.”*** | Yes 1  No 0  No Response -99 | |
| 54 | | Is it you or your spouse who is military/police personnel? | 1) Myself  2) Spouse  3) Son  4) Daughter  5) Other (specify): _____ | |
| 55 | | In total how many members of your immediate family are part of the FARDC? | Number__________________________ | |
| 56 | | What is your/his rank in this service? | \| **1** \| Soldier 1^st^ class \| \| --- \| --- \| \| **2** \| Soldier 2^nd^ class \| \| **3** \| Corporal \| \| **4** \| Sargent \| \| **5** \| Sargent major \| \| **6** \| 1^st^ Sargent \| \| **7** \| 1^st^ Sargent Adjoint \| \| **8** \| Adjudant de 2^ème^ classe \| \| **9** \| Adjudant \| \| **10** \| Adjudant in chef \| \| **11** \| Second Lieutenant \| \| **12** \| Lieutenant \| \| **13** \| Captain \| \| **14** \| Major \| \| **15** \| Lieutenant Colonel \| \| **16** \| Colonel \| \| **17** \| General Bragadier \| \| **18** \| Lieutenant General \| \| **19** \| General of Army Corps \| \| **20** \| General or Army \| | |
| 57 | | Have you seen a billboard or banner about family planning that shows a military family? | 0 No  1 Yes  88 Don’t know | |
| 58 | | In what province were you born?  If Kinshasa, skip to question “K” | List provinces | |
| 59 | | For how many years have you lived in Kinshasa?  0=less than one year | _____________Years | |
| **Thank the respondent for her time**  *The respondent is finished, but there are still 2 more questions for you to complete outside the home.* | | | | |
| **LOCATION** | | | | |
| K | | **Location**  *Take a GPS point near the entrance to the household. Record location when the accuracy is smaller than 6m.*  *GPS coordinates can only be collected when outside.* | Record Location | |
| **QUESTIONNAIRE RESULT** | | | | |
| L | **How many times have you visited this household to interview this female respondent?** | | 1^st^ time 1  2^nd^ time 2  3^rd^ time 3 | |
| M | | **Questionnaire result**  *Record the result of the Female Questionnaire* | Completed 1  Not at home 2  Postponed 3  Refused 4  Partly completed 5  Incapacitated 6 | |
