# Supplementary material for: The gap in contraceptive knowledge and use between the military and non-military populations of Kinshasa, DRC, 2016–2019
Source: PLoS One. 2021 Jul 27;16(7):e0254915. doi: 10.1371/journal.pone.0254915 (PMC8315532; doi:10.1371/journal.pone.0254915)
Supplement: S2 File — (DOCX) [file pone.0254915.s002.docx]

**S2 File. 2016 military survey questionnaire, French**

| **Questionnaire Femme** |
| --- |

| **NO** | **QUESTIONS ET FILTRES** | | | | **CATEGORIES CODEES** | | | | | | | **ALLER A** | | | |
| --- | --- | --- | --- | --- | --- | --- | --- | --- | --- | --- | --- | --- | --- | --- | --- |
| **IDENTIFICATION**  **Veuillez enregistrer l’information suivante avant de commencer l’entretien** | | | | | | | | | | | | | | | |
| A | | **Êtes-vous dans le bon ménage ?**  **Voici une photo de l’entrée de la maison prise lors du Questionnaire ménage.**  **SI NON, ALLEZ INTEROGER LE BON MENAGE.**  [ODK affichera la photo prise au cours du Questionnaire ménage.] | | | Oui 1  Non 0 | | | | | | |  | | | |
|  | |  | | |  | | | | | | |  | | | |
| B | | **Votre nom:** [nom de l’enquêtrice saisi dans le Questionnaire Ménage]  **Est-ce bien votre nom?** | | | Oui 1  Non 0 | | | | | | |  | | | |
|  |  | **Indiquez votre nom ci-dessus.**  *Veuillez saisir votre nom:* | | | Prénom de l’enquêtrice | | | | | | |  |  |  |  |
| C | | **Date et heure locale.** ODK les affichera sur l’écran.  **La date et l’heure sont-elles correctes?** | | | Oui 1  Non 0 | | | | | | | Aller à E si Oui | | | |
| D | | **Veuillez saisir la date et l’heure.** | | | \| **Jour** \| **Mois** \| **Année** \| \| --- \| --- \| --- \| \|  \|  \|  \| \| **Heure** \| **Minutes** \| **AM/PM** \| \|  \|  \|  \| | | | | | | |  | | | |
| E | | **Les informations suivantes proviennent du Questionnaire ménage. Veuillez les relire pour vous assurer que vous interrogez la bonne personne.**  [ODK affichera la province, la ville, la commune, et le quartier pour les ZD de Kinshasa OU la province, le district, l’aire de santé, et le village pour les ZDde Kongo Central . En plus, la Zone de Dénombrement, le Numéro de la Structure, et le Numéro du Ménage saisis dans le Questionnaire Ménage associé à ce Questionnaire Femme seront affichés.]  **Les informations ci-dessus, sont-elles correctes?** | | | Oui 1  Non 0 | | | | | | |  | | | |
|  | | **CONTROLE: La personne que vous allez interroger est [Nom de l’enquêtée]. Est-ce correct?**  *Si le prénom est mal orthographié, sélectionnez “oui” et actualisez le prénom à la question “J”*  *S’il s’agit de la mauvaise personne, vous avez deux options:*  *(1) Sortir et ignorer les changements apportés à ce questionnaire. Ouvrir le bon questionnaire.*  *Ou*  *(2) Trouver et interroger la personne dont le nom s’est affiché précédemment* | | | Oui 1  Non 0 | | | | | | |  | | | |
| F | | La personne à interroger est-elle présente et disponible pour répondre au questionnaire aujourd’hui? | | | Oui 1  Non 0 | | | | | | | Aller à J si NON | | | |
| G | | Connaissez-vous l’enquêtée? | | | Oui, très bien 1  Oui, bien 2  Oui, mais pas très bien 3  Non 4 | | | | | | |  | | | |
| G2 | | L’enquêtée a-t-elle déjà participé à une enquête PMA2020 ? | | | Oui 1  Non 0  Ne sait pas -88  Pas de réponse -99 | | | | | | |  | | | |
| **CONSENTEMENT ECLAIRE**  **Veuillez trouver la femme entre 15 et 49 ans associée à ce Questionnaire Femme. L’entretien doit se dérouler en privé. Veuillez lire le message suivant à la personne interrogée:** | | | | | | | | | | | | | | | |
| Bonjour. Je m’appelle ________________________________ et je travaille pour l’École de Santé Publique de Kinshasa en collaboration avec le Ministère de la Santé. Nous menons actuellement une enquête à Kinshasa et à Kongo Central sur plusieurs thèmes liés à la santé reproductive des femmes. Nous apprécions beaucoup que vous fassiez partie de cette enquête. Les informations que nous collecterons aideront à informer le gouvernement afin de mieux planifier les services de santé. Le questionnaire prend généralement entre 15 et 20 minutes. Toutes les informations que vous nous donnerez seront strictement confidentielles et ne seront partagées avec personne d’autre que les membres de notre équipe.  La participation à cette enquête est volontaire, et s’il y a une question à laquelle vous ne souhaitez pas répondre, faîtes le moi savoir et je passerai à la suivante; ou vous pouvez également interrompre l’entretien à tout moment. Cependant, nous espérons que vous accepterez de participer à cette enquête car votre point de vue est important.  Avant de continuer, avez-vous des questions sur cette enquête? | | | | | | | | | | | | | | | |
| H | | Veuillez remettre un exemplaire papier du Formulaire de Consentement à la personne interrogée et lui expliquer. Puis, demandez: **Puis-je commencer l’interview à présent ?** | | | Oui 1  Non, 0 | | | | | | | Aller à K si NON | | | |
|  | | **La signature de l’enquêtée**  *Veuillez demander à l’enquêtée de signer ou de cocher la case indiquant leur accord pour participer.* | | | Recueillir la signature:  Case à cocher: ☐ | | | | | | |  | | | |
| I | | **Nom de l’enquêtrice: [**Nom de l’enquêtrice saisi dans le Questionnaire Ménage]  *Veuillez cocher la case en tant que témoin au consentement.* | | | \|  \| \| --- \| | | | | | | |  | | | |
| J | | **Prénom de l’enquêtée**  [ODK affichera le prénom de l’enquêtée enregistré au cours du Questionnaire ménage.]  *Vous pouvez corriger si le nom est mal orthographié, mais vous devez interroger la personne dont le prénom s’est affiché ci-dessous.* | | | \|  \| \| --- \| | | | | | | |  | | | |
| **Section 1 – Information sur l’enquêtée, Statut Conjugal et Caractéristique du Ménage**  *J’aimerais d’abord vous poser des questions concernant vos caractéristiques socio-économiques.* | | | | | | | | | | | | | | | |
| 0 | | **En quel mois et quelle année êtes-vous née ?** | | \| Mois \|  \| \| --- \| --- \| \| Année \|  \| | | | | | | | | | | |  |
| 1 | | **Quel âge aviez-vous à votre dernier anniversaire ?** | | \| Age \|  \| \| --- \| --- \| | | | | | | | | | | |  |
| 2 | | **Quel est le plus haut niveau d'études que vous avez atteint?** | | Jamais scolarisée……………………...0  Primaire 1  Secondaire 2  Supérieur………………..……….3  Pas de réponse………………………-99 | | | | | | | | | | |  |
| 3 | | **Etes-vous actuellement mariée ou vivez-vous avec un homme comme si vous étiez mariés ?**  *RELANCER: Si la réponse est "non", demandez si l’enquêtée est divorcée, séparée ou veuve.* | | Non, jamais en union 0  Oui, actuellement mariée 1  Oui, vit avec un homme 2  Pas en union actuellement: Divorcée / séparée  3  Pas en union actuellement: Veuve 4  Pas de réponse -99 | | | | | | | | | | | Aller à 8 si NON, jamais en union |
| 4 | | **Avez-vous déjà été mariée ou avez-vous déjà vécu avec un homme au moins une fois ou plus d’une fois ?** | | Seulement une fois 1  Plus d’une fois 2  Pas de réponse -99 | | | | | | | | | | | Aller à 5b si SEULEMENT UNE FOIS |
| 5a | | **En quel mois et quelle année avez-vous commencé à vivre avec votre PREMIER mari/conjoint ?**  *Saisir JAN 2020 si pas de réponse.* | | \| Mois \|  \| \| --- \| --- \| \| Année \|  \| | | | | | | | | | | |  |
|  | | Si l’enquêtée avait moins de 15 ans lors de son mariage, ODK affichera:  **CONTROLE: Selon la réponse que vous avez inscrite pour QF5a, la participante avait moins de 15 ans lors de son premier mariage, avez-vous saisi la bonne réponse?** | | Oui 1  Non 0 | | | | | | | | | | |  |
| 5b | | **Maintenant j’aimerais vous demander à partir de quand vous avez commencé à vivre avec votre mari/conjoint ACTUEL. C’était en quel mois et en quelle année?**  *Saisir JAN 2020 si pas de réponse.* | | \| Mois \|  \| \| --- \| --- \| \| Année \|  \| | | | | | | | | | | |  |
|  | | Si l’enquêtée avait moins de 15 ans lors de son mariage ODK affichera:  **CONTROLE: Selon la réponse que vous avez saisie pour QF5b, l’enquêtée avait moins de 15 ans lors de son premier mariage. Avez-vous saisi la bonne réponse?** | | Oui 1  Non 0 | | | | | | | | | | |  |
|  | | **CONTROLE 3:** Actuellement mariée/en cohabitation ? | | Oui 1  Non 0 | | | | | | | | | | | Aller à 8 si Non |
| 6 | | **Votre mari/conjoint a-t-il d’autres femmes ou vit-il avec d’autres femmes comme s’il était marié ?** | | Oui 1  Non 0  Ne sait pas -88  Pas de réponse -99 | | | | | | | | | | |  |
| 7 | | **Votre mari/conjoint vit-il avec vous en ce moment ou habite-t-il ailleurs ?** | | Vit avec l’enquêtée 1  Vit ailleurs 2  Pas de réponse -99 | | | | | | | | | | |  |
| **Section 2 – Reproduction, Grossesses &Intentions de fécondité**  *J’aimerais maintenant vous poser des questions concernant toutes les naissances que vous avez eues durant votre vie.* | | | | | | | | | | | | | | | |
| 8a | | **Combien de fois avez-vous donné naissance?**  *Saisir -99 si pas de réponse. 0 est une réponse possible.* | | \| Nombre de naissance(s) \|  \| \| --- \| --- \| | | | | | | |  | | Aller à 13 si 0 | | |
|  | | **Toutes ces naissances étaient-elles vivantes?**  *Si non, revenir en arrière et changer QF8 pour saisir uniquement les naissances vivantes.* | | Oui 1  Non 0 | | | | | | | |  | | | |
| 8b | | **A combien de garçons et de filles nés vivants avez-vous donné naissance ?** | | \| Nombre \|  \| \| --- \| --- \| | | | | | | | |  | | | |
| 8c | | **Avez-vous au moins une fois donné naissance à un garçon ou une fille qui est née vivant mais qui est décédé par la suite?**  **SI NON, RELANCER :**  Un bébé qui a crié ou fait un mouvement, ou qui a émis un son, ou qui a essayé de respiré ou qui a montré des signes de vie mais qui n’a pas survécu? | | Oui 1  Non 0 | | | | | | | | Aller sur 8e si Non | | | |
| 8d | | **Combien d’enfants sont décédés ?**  *Saisir -88 si ne sait pas et -99 si pas de réponse. Remplacez QF8c à ‘Non’ s’il n’y avait pas de morts.* | | \| Nombre \|  \| \| --- \| --- \| | | | | | | | |  | | | |
|  | | **LIRE A HAUTE VOIX : Pour être sûre que j’ai compris : vous avez eu un total de ____ naissance(s) pendant votre vie, avec _____ garçon(s) ou fille(s) qui sont nés vivants.** | | Oui 1  Non 0 | | | | | | | | Si Non, aller sur 8a-c pour relancer et corriger. | | | |
| 8e | | **Quand avez-vous eu votre PREMIÈRE naissance?**  *Saisir la date de la PREMIERE naissance vivante. Celle-ci peut* ê*tre calculée en remontant le temps à partir d’événements mémorables si nécessaire.*  *Saisir JAN 2020 si pas de réponse.* | | \| Mois \|  \| \| --- \| --- \| \| Année \|  \| | | | | | | | |  | | | |
| 9 | | **Quand avez-vous eu votre DERNIÈRE naissance?**  *Saisir la date de la DERNIERE naissance vivante. Celle-ci peut être calculée en remontant le temps à partir d’événements mémorables si nécessaire.*  *Saisir JAN 2020 si pas de réponse* | | \| Mois \|  \| \| --- \| --- \| \| Année \|  \| | | | | | | | | Aller à 11 si pas au cours de l’année passée et/ou si Q8 est 1 | | | |
| 10 | | **Quand avez-vous eu votre avant-dernière naissance (la plus récente avant votre dernière naissance) ?**  *Saisir la date de l’AVANT DERNIERE naissance. Celle-ci peut être calculée en remontant le temps à partir d’évènements mémorables si nécessaire.*  *Saisir JAN 2020 si pas de réponse* | | \| Mois \|  \| \| --- \| --- \| \| Année \|  \| | | | | | | | |  | | | |
| 11 | | **Votre dernier bébé/ enfant est-il encore en vie ?** | | Oui 1  Non 0  Ne sait pas -88 | | | | | | | | Aller à 13 si OUI | | | |
| 12 | | **Quand votre dernier bébé/ enfant est-il mort ?**  *Veuillez saisir la date de la mort de l’enfant.*  *Celle-ci peut être calculée en remontant le temps à partir d’évènements mémorables si nécessaire.*  *Saisir JAN 2020 si pas de réponse.* | | \| Mois \|  \| \| --- \| --- \| \| Année \|  \| | | | | | | | |  | | | |
| 13 | | **Quand vos dernières règles ont-elles commencé ?**  *Si vous choisissez jours, semaines, mois ou années, vous saisirez un nombre sur le prochain écran. Saisir 0 jours pour aujourd’hui, pas 0 semaine/mois/année * | | Il y a ______ jours | | | | | |  | |  | | | |
|  |  |  |  | Il y a ______ semaines | | | | | |  | |  |  |  |  |
|  |  |  |  | Il y a _______ mois | | | | | |  | |  |  |  |  |
|  |  |  |  | Il y a _______ années | | | | | |  | |  |  |  |  |
|  |  |  |  | Ménopause/Hystérectomie 5  Avant la dernière naissance 6  Jamais eu de règles 7  Pas de réponse -99 | | | | | | | |  |  |  |  |
| 14 | | **Etes-vous actuellement enceinte?** | | Oui 1  Non 0  Pas sûre 2  Pas de réponse -99 | | | | | | | | Aller à 16 si Non ou Pas sûre | | | |
| 15 | | **De combien de mois êtes-vous enceinte ?**  **La naissance la plus récente était: [Date de la naissance la plus récente]**  *Veuillez saisir le nombre de mois*  *révolus. Saisir -88 si ne sait pas et -99 si pas de réponse.* | | \| Nombre de mois \|  \| \| --- \| --- \| | | | | | | |  | |  | | |
|  | | **CONTROLE 14:** Enceinte actuellement? | | Oui 1  Non 0 | | | | | | | | 16a si non 16b si oui | | | |
| 16a | | **Je voudrais maintenant vous poser quelques questions sur l’avenir.**  **Voudriez-vous avoir un / une autre enfant ou préféreriez-vous ne pas / plus avoir d'enfants ?** | | Avoir un autre enfant 1  Ne plus en avoir 2  Ne peut plus concevoir 3  Indécise/Ne sait pas -88  Pas de réponse -99 | | | | | | | | Aller à 17a si 1 et 18 pour toutes autres réponses | | | |
| 16b | | **Je voudrais maintenant vous poser quelques questions sur l’avenir.**  **Après l'enfant que vous attendez maintenant, voudriez-vous un autre enfant ou préféreriez-vous ne plus avoir d'enfants** | | Avoir un autre enfant 1  Ne plus en avoir 2  Ne peut plus concevoir 3  Indécise / Ne sait pas -88  Pas de réponse -99 | | | | | | | | Aller à 17b si 1 et 18 pour toutes autres réponses | | | |
| 17a | | **Combien de temps voudriez-vous attendre à partir de maintenant avant votre prochaine naissance ?**  *Si vous choisissiez mois ou années, vous saisirez un nombre dans le prochain écran.*  *Choisissez « Années » si plus de 36 mois.* | | \| Mois \|  \| \| --- \| --- \| \| Année \|  \| | | | | | | | |  | | | |
|  |  |  |  | Bientôt / maintenant 1  Dit ne pas pouvoir tomber enceinte 2  Autre 3  Indécise /Ne sait pas -88  Pas de réponse -99 | | | | | | | |  |  |  |  |
| 17b | | **Après la naissance de l’enfant que vous attendez maintenant, combien de temps voudriez-vous attendre avant la naissance d’un autre enfant?**  *Si vous choisissiez mois ou années, vous saisirez un nombre dans le prochain écran.*  *Choisissez « Années » si plus de 36 mois.* | | \| Mois \|  \| \| --- \| --- \| \| Année \|  \| | | | | | | | |  | | | |
|  |  |  |  | Bientôt / maintenant 1  Dit ne pas pouvoir tomber enceinte 2  Autre 3  Indécise / NSP -88  Pas de réponse -99 | | | | | | | |  |  |  |  |
|  | | **CONTROLE 8:** Nombre de naissances ?  **CONTROLE 14**: Enceinte actuellement? | | \| Nombre de naissance(s) \|  \| \| --- \| --- \| | | | | | | |  | | Aller à 19 si 0 naissance et 14: NON.  Aller à 18a si 14: NON et 18b si 14: OUI | | |
|  |  |  |  | Oui………………………………… 1  Non………………………………….0 | | | | | | | |  | | | |
| 18a | | **J’aimerais maintenant vous poser une question sur votre dernière naissance. Quand vous êtes tombée enceinte, vouliez-vous être enceinte à ce moment-là, est ce que vous vouliez avoir un enfant plus tard ou est-ce que vous ne vouliez pas/ plus avoir d’enfant?** | | À ce moment 1  Plus tard 2  Pas d’enfants du tout 3  Pas de réponse -99 | | | | | | | |  | | | |
| 18b | | **J’aimerais maintenant vous poser une question sur votre grossesse actuelle.**  **Quand vous êtes tombée enceinte, vouliez-vous être enceinte à ce moment-là, est ce que vous vouliez avoir un enfant plus tard, ou est-ce que vous ne vouliez pas/ plus avoir d’enfant?** | | À ce moment 1  Plus tard 2  Pas d’enfants du tout 3  Pas de réponse -99 | | | | | | | |  | | | |
| **Section 3 – Contraception**  *J’aimerais maintenant vous poser des questions sur le planning familial – les façons ou méthodes qu’un couple peut utiliser pour retarder ou éviter une grossesse.*  *ODK affichera une image sur les écrans pour quelques méthodes. Si l’enquêtée dit qu’elle n’a pas entendu parler de la méthode ou si elle hésite à répondre, veuillez lire la description de la méthode puis lui montrer l’image, si applicable.* | | | | | | | | | | | | | | | |
| 19 | | **Avez-vous déjà entendu parler de la stérilisation féminine ?**  **RELANCER**: Les femmes peuvent avoir uneopération pour ne plus avoir d'enfants. | | Oui 1  Non 0  Pas de réponse -99 | | | | | | | | | |  | |
| 19 | | **Avez-vous déjà entendu parler de la stérilisation masculine ?**  **RELANCER :** Les hommes peuvent avoir uneopération pour ne plus avoir d'enfants. | | Oui 1  Non 0  Pas de réponse -99 | | | | | | | | | |  | |
| 19 | | **Avez-vous déjà entendu parler des implants contraceptifs ?**  **RELANCER :** Les femmes peuvent se faire insérer par un médecin ou une infirmière un bâtonnet ou plus sous la peau du haut du bras pour les empêcher de tomber enceinte, pendant une année ou plus  [ODK AFFICHERA UNE IMAGE DE LA METHODE SUR L’ECRAN] | | Oui 1  Non 0  Pas de réponse -99 | | | | | | | | | |  | |
| 19 | | **Avez-vous déjà entendu parler des DIU / Stérilet?**  **RELANCER:** Les femmes peuvent avoir un stérilet qu'un médecin ou une infirmière leur place dans l'utérus.  [ODK AFFICHERA UNE IMAGE DE LA METHODE SUR L’ECRAN] | | Oui 1  Non 0  Pas de réponse -99 | | | | | | | | | |  | |
| 19 | | **Avez-vous déjà entendu parler des injectables ?**  **RELANCER :** Les femmes peuvent avoir une injection faite par du personnel de santé qui les empêche de tomber enceinte pendant un mois ou plus.  [ODK AFFICHERA UNE IMAGE DE SAYANA PRESS EN PLUS DEPO PROVERA SUR L’ECRAN] | | Oui 1  Non 0  Pas de réponse -99 | | | | | | | | | |  | |
| 19 | | **Avez-vous déjà entendu parler de la pilule (contraceptive) ?**  **RELANCER :** Les femmes peuvent prendre une pilule chaque jour pour éviter de tomber enceinte.  [ODK AFFICHERA UNE IMAGE DE LA METHODE SUR L’ECRAN] | | Oui 1  Non 0  Pas de réponse -99 | | | | | | | | | |  | |
| 19 | | **Avez-vous déjà entendu parler de la pilule du lendemain / contraception d’urgence?**  **RELANCER :** Les femmes peuvent prendre pendant trois jours après des rapports sexuels non protégés des pilules spéciales qui les empêchent de tomber enceintes.  [PAS D’IMAGE] | | Oui 1  Non 0  Pas de réponse -99 | | | | | | | | | |  | |
| 19 | | **Avez-vous déjà entendu parler des condoms (préservatifs masculins)?**  **RELANCER :**Les hommes peuvent mettre une capote encaoutchouc sur leur pénis avant les rapports sexuels.  [ODK AFFICHERA UNE IMAGE DE LA METHODE SUR L’ECRAN] | | Oui 1  Non 0  Pas de réponse -99 | | | | | | | | | |  | |
| 19 | | **Avez-vous déjà entendu parler des condoms (préservatifs féminins)?**  **RELANCER:** Les femmes peuvent placer un fourreau dans leur vagin avant les rapports sexuels.  [ODK AFFICHERA UNE IMAGE DE LA METHODE SUR L’ECRAN] | | Oui 1  Non 0  Pas de réponse -99 | | | | | | | | | |  | |
| 19 | | **Avez-vous déjà entendu parler du diaphragme ?**  **RELANCER:** Les femmes peuvent placer unerondelle de latex ou un petit "bonnet" sur le col de l'utérus avant les  rapports sexuels.  [ODK AFFICHERA UNE IMAGE DE LA METHODE SUR L’ECRAN] | | Oui 1  Non 0  Pas de réponse -99 | | | | | | | | | |  | |
| 19 | | **Avez-vous déjà entendu parler des comprimés, la mousse, ou la gelée contraceptive ?**  **RELANCER :** Les femmes peuvent mettre dans leurvagin une crème avant tout rapport sexuel afin de tuer lesspermatozoïdes de l'homme. Cette crème peut être aussi mise sur le  diaphragme.  [ODK AFFICHERA UNE IMAGE DE LA METHODE SUR L’ECRAN] | | Oui 1  Non 0  Pas de réponse -99 | | | | | | | | | |  | |
| 19 | | **Avez-vous déjà entendu parler de la méthode des jours fixes ou le collier du cycle ?**  **RELANCER:** Les femmes utilisent un collier de perles de couleur différente pour connaitre les jours où elles peuvent tomber enceinte. Durant ces jours, elles utilisent un condom ou elles s’abstiennent de rapports sexuels.  [ODK AFFICHERA UNE IMAGE DE LA METHODE SUR L’ECRAN] | | Oui 1  Non 0  Pas de réponse -99 | | | | | | | | | |  | |
| 19 | | **Avez-vous déjà entendu parler de la méthode d’allaitement exclusif ou MAMA?**  [PAS DE DESCRIPTION ; PAS D’IMAGE] | | Oui 1  Non 0  Pas de réponse -99 | | | | | | | | | |  | |
| 19 | | **Avez-vous déjà entendu parler de la méthode du rythme ?**  **RELANCER :** Pour éviter une grossesse, les femmes n’ont pas de rapports sexuels les jours du mois où elles pensent qu’elles peuvent tomber enceintes.  [PAS D’IMAGE] | | Oui 1  Non 0  Pas de réponse -99 | | | | | | | | | |  | |
| 19 | | **Avez-vous déjà entendu parler de la méthode du retrait / coït interrompu ?**  **RELANCER :** Les hommes peuvent faire attention et se retirer avant l'éjaculation.  [PAS D’IMAGE] | | Oui 1  Non 0  Pas de réponse -99 | | | | | | | | | |  | |
| 19 | | **Avez-vous entendu parler d’autres moyens ou méthodes qu’une femme ou un homme peut utiliser pour éviter une grossesse ?** | | Oui 1  Non 0  Pas de réponse -99 | | | | | | | | | |  | |
|  | | **CONTROLE 14:** Enceinte actuellement? | | Oui 1  Non 0 | | | | | | | | | | Aller à 23 si OUI | |
| 20 | | **Est ce que vous, ou votre partenaire faites actuellement quelque chose ou utilisez-vous actuellement une méthode pour retarder ou éviter une grossesse?** | | Oui 1  Non 0 | | | | | | | | | | Aller à 23 si NON | |
| 21 | | **Que faites-vous pour retarder ou éviter une grossesse?**  **Relancer : Faites-vous quelque chose d’autre?**  *Sélectionnez toutes les méthodes mentionnées. Assurez-vous de faire dérouler la liste JUSQU’EN BAS pour voire tous les choix possibles* | | Stérilisation féminine  Stérilisation masculine  Implants  DIU/Stérilet  Injectable  Pilule  Pilule du lendemain Préservatif masculin  Préservatif féminin  Diaphragme  Mousse/Gelée spermicide  Méthode des jours fixes/Collier du cycle  MAMA  Méthode du rythme  Retrait  Autres méthodes traditionnelles  Pas de réponse -99 | | | 1  2  3  4  5  7  8  9  10  11  12  13  14  30  31  39  -99 | | | | | | | Saut en fonction de la méthode la plus efficace uniquement  Si Injectables est sélectionnée, aller à 21a  Si MAMA est sélectionnée, aller à 21b  Si stérilisation masculine ou féminine sont sélectionnées sans la sélection des injectables ou MAMA, aller à 22  Si MAMA, injectables, et stérilisation ne sont pas sélectionnées, aller à 26b | |
| 21a | | **RELANCER :** Est-ce que l’injection a été administrée par seringue ou par petite aiguille ?  *Veuillez montrer l’image à l’enquêtée.*  [ODK AFFICHERA UNE IMAGE DE LA METHODE SUR L’ECRAN] | | Seringue 1  Petite aiguille (SayanaPress) 2  Les deux 3  Pas de réponse -99 | | | | | | | | | | Aller au CONTRÔLE avant QF22 | |
| 21b | | **Allaitez-vous votre enfant pour retarder ou éviter une grossesse ?** | | Oui 1  Non 0  Pas de réponse -99 | | | | | | | | | |  | |
|  | | **CONTRÔLE :** Utilise-elle la stérilisation féminine ou masculine ? | | Stérilisation féminine 1  Stérilisation masculine 2  Aucun des éléments ci-dessus .-77 | | | | | | | | | | Aller à 26b si -77 | |
| 22 | | **Le prestataire de santé qui vous a fourni cette méthode vous a-t-il dit, où a-t-il dit à votre conjoint, que cette méthode est permanente ?** | | Oui 1  Non 0  Pas de réponse -99 | | | | | | | | | | Aller à 26b | |
| 23 | | **Connaissez-vous un endroit où vous pouvez vous procurer une méthode de planification familiale ?** | | Oui 1  Non 0  Pas de réponse -99 | | | | | | | | | |  | |
|  | | **CONTROLE 14:** Enceinte actuellement? | | Oui 1  Non 0 | | | | | | | | | | Aller à  24b si OUI | |
| 24a | | **Vous avez dit que vous n’utilisez pas de méthode contraceptive en ce moment. Pensez-vous que vous utiliserez une méthode contraceptive pour retarder ou éviter une grossesse dans le futur ?** | | Oui 1  Non 0  Pas de réponse -99 | | | | | | | | | |  | |
| 24b | | **Pensez-vous que vous utiliserez une méthode contraceptive pour retarder ou éviter une grossesse dans le futur ?** | | Oui 1  Non 0  Pas de réponse -99 | | | | | | | | | |  | |
| 25 | | **Au cours des 12 derniers mois, avez-vous utilisé une méthode ou fait quelque chose pour retarder ou éviter une grossesse?** | | Oui 1  Non 0  Pas de réponse -99 | | | | | | | | | | Aller à 41 si NON | |
| 26 | | **Quelle méthode avez-vous utilisé le plus récemment?**  **RELANCER: Y en a-t-il une autre?**  *Sélectionnez la méthode la plus efficace (par ordre d’efficacité décroissante dans la liste). Faire dérouler la liste jusqu’en bas pour voir tous les choix possibles.* | | ~~Stérilisation féminine~~  ~~Stérilisation masculine~~  Implants  DIU/Stérilet  Injectable  Pilule  Pilule du lendemain  Préservatif masculin  Préservatif féminin  Diaphragme  Mousse/Gelée spermicide  Méthode des jours fixes/Collier du cycle  MAMA  Méthode du rythme  Retrait  Autres méthodes traditionnelles Pas de réponse -99  Pas de réponse -99 | | | | ~~1~~  ~~2~~  3  4  5  7  8  9  10  11  12  13  14  30  31  39  -99 | | | | | | Aller à QF26b sauf si ‘injectables’ est sélectionnés | |
| 26a | | **RELANCER :** Est-ce que l’injection a été administrée par seringue ou par petite aiguille ?  *Veuillez montrer l’image à l’enquêtée.*  [ODK AFFICHERA UNE IMAGE DE LA METHODE SUR L’ECRAN] | | Seringue 1  Petite aiguille (SayanaPress) 2  Les deux 3  Pas de réponse -99 | | | | | | | | | |  | |
| 26b | | **Avant de commencer à utiliser [MÉTHODE LA PLUS RÉCENTE / MÉTHODE ACTUELLE], avez-vous parlé avec votre mari/partenaire de la possibilité d’utiliser une méthode contraceptive ?** | | Oui 1  Non 0  Ne sait pas -88  Pas de réponse -99 | | | | | | | | | |  | |
| 27 | | **Quand avez-vous commencé à utiliser [MÉTHODE LA PLUS RÉCENTE / MÉTHODE ACTUELLE]?**  *Calculer la date en remontant le temps à partir d'évènements mémorables si nécessaire.*  **Age d’utiliser la contraception pour la première fois : [l’Age enregistré pour QF20]**  **Naissance la plus récente : [mm-aaaa]**  **Mariage actuel : [mm-aaaa]**  *Doit être au moins l’âge à partir duquel elle a commencé à utiliser une méthode contraceptive (QF20).*  *Doit être avant la date d'aujourd'hui. L'enquêtée doit avoir au moins dix ans.*  *Saisir JAN 2020 si pas de réponse.* | | \| Mois \|  \| \| --- \| --- \| \| Année \|  \| | | | | | | | | | |  | |
|  | | **CONTROLE 20:** Utilise en ce moment un contraceptif? | | Oui 1  Non 0 | | | | | | | | | | Aller à 30 si OUI | |
| 28 | | **Quand avez-vous arrêté d’utiliser [MÉTHODE LA PLUS RÉCENTE]?**  *Veuillez saisir la date. Calculez la date en remontant le temps à partir d’événements mémorables si nécessaire. Doit être après QF29.*  *Saisir JAN 2020 si pas de réponse.* | | \| Mois \|  \| \| --- \| --- \| \| Année \|  \| | | | | | | | | | |  | |
| 29 | | **Pourquoi avez-vous arrêté d’utiliser [MÉTHODE LA PLUS RÉCENTE]?** | | Peu de rapports sexuels/conjoint absent 1  Est tombée enceinte en l’utilisant 2  Voulait tomber enceinte 3  Mari/conjoint en désaccord 4  Voulait une méthode plus efficace 5  Aucune méthode disponible 6  Problèmes de santé 7  Peur d’effets secondaires 8  Manque d’accès/trop loin 9  Coûte trop cher 10  Utilisation peu pratique 11  Fataliste 12  Des difficultés à tomber enceinte/ménopausée 13  Interfère avec les processus  du corps 14  Autre 15  Ne sait pas -88  Pas de réponse -99 | | | | | | | | | |  | |
| 30 | | **Vous avez commencé à utiliser [METHODE ACTUELLE / PLUS RECENTE] en [DATE DE QF27]. Où l’avez-vous obtenue à ce moment-là ?**  *Descendre jusqu’en bas pour voir toutes les réponses possibles* | | **SECTEUR PUBLIC:**  Centre hospitalier national ..……………….11  Clinique de Planning familiale ………………. 12  Centre/Poste de santé…………………13  Maternité……………………………………14  Relais communautaire …………15  Centre hospitalier régional …………………16  **SECTEUR MEDICAL PRIVE:**  Hôpital/Clinique privé…………………………..…. 21  Pharmacie…………………………….……………………22  ONG …………………….…………………….23  Centre de santé privé…………………….………………24  Cabinet médical privé…………………….………………25  Médecin privé…………………….…………………….26  Infirmier ambulant………………….…………………….27  Relais communautaire / agent de santé communautaire (ASC)………………….…………………….28  Etudiants – agents de santé communautaire …………………………………………29  **AUTRES SOURCES:**  Boutique………………….…………………….………………31  Institution religieuse ……………….……………….32  Ami(e)/Parent(e) ……………………………………..33  Bar/Boîte de nuit…………………….…………………….34  LIGABLO/Kiosk…………………….………………35  Chayeur……………………………………………………..36  Autre ……………………………………………………. 37  Pas de réponse -99 | | | | | | | | | |  | |
| 31 | | **Quand vous avez obtenu [MÉTHODE LA PLUS RÉCENTE/ ACTUELLE], l’agent de santé ou de planification familiale vous a-t-il parlé des effets secondaires ou des problèmes que vous pourriez avoir en utilisant une méthode pour retarder ou éviter une grossesse ?** | | Oui 1  Non 0  Pas de réponse -99 | | | | | | | | | | Aller à 33 si NON | |
| 32 | | **Vous a-t-on dit ce qu'il fallait faire si vous aviez ces effets secondaires ou des problèmes ?** | | Oui 1  No 0  Pas de réponse -99 | | | | | | | | | |  | |
| 33 | | **À ce moment-là, vous a-t-on parlé d’autres méthodes que [MÉTHODE ACTUELLE/ LA PLUS RÉCENTE] que vous pourriez utiliser?** | | Oui 1  Non 0  Pas de réponse -99 | | | | | | | | | |  | |
| 34 | | **Pendant cette visite, avez-vous obtenu la méthode que vous souhaitiez pour éviter ou retarder une grossesse ?** | | Oui 1  Non 0  Pas de réponse -99 | | | | | | | | | | Aller à 36 si OUI | |
| 35 | | **Pourquoi n’avez-vous pas obtenu la méthode que vous souhaitiez?** | | Rupture de stock ce jour-là 1  Méthode pas disponible 2  Prestataire pas formé pour fournir cette méthode 3  Prestataire recommandait une autre méthode 4  Pas éligible pour cette méthode 5  A décidé de ne plus utiliser cette méthode 6  Trop cher 7  Autre 8  Pas de réponse -99 | | | | | | | | | |  | |
| 36 | | **Pendant cette visite, qui a pris la décision concernant la méthode adoptée ?** | | Enquêtée seule 1  Prestataire 2  Partenaire 3  Enquêtée et prestataire 4  Enquêtée et partenaire 5  Autre 6  Pas de réponse -99 | | | | | | | | | |  | |
|  | | **CONTROLE 30:Vous avez commencé à utiliser [METHODE ACTUELLE / PLUS RECENTE] en [DATE DE QF27]. Où l’avez-vous obtenue à ce moment-là ?** | | **SECTEUR PUBLIC:**  Centre hospitalier national ..……………….11  Clinique de Planning familiale ………………. 12  Centre/Poste de santé…………………13  Maternité……………………………………14  Relais communautaire …………15  Centre hospitalier régional …………………16  **SECTEUR MEDICAL PRIVE:**  Hôpital/Clinique privé…………………………..…. 21  Pharmacie…………………………….……………………22  ONG …………………….…………………….23  Centre de santé privé………………….…………………24  Cabinet médical privé………………….…………………25  Médecin privé…………………….…………………….26  Infirmier ambulant………………….…………………….27  Relais communautaire / agent de santé communautaire (ASC) ……….28  Etudiants – agents de santé communautaire…………………………………………….29  **AUTRES SOURCES:**  Boutique………………….…………………….………………31  Institution religieuse ……………….……………….32  Ami(e)/Parent(e) ……………………………………..33  Bar/Boîte de nuit…………………….…………………….34  LIGABLO/Kiosk…………………….………………35  Chayeur……………………………………………………..36  Autre.. ……………………………………………………. 37  Pas de réponse -99 | | | | | | | | | | Aller à 41 si 30 est 31 OU 37, | |
| 37 | | **Retourneriez-vous voir ce prestataire de santé?**  **[ODK affichera le type de prestataire sélectionné à QF30]** | | Oui 1  Non 0  Pas de réponse -99 | | | | | | | | | |  | |
| 38 | | **Recommanderiez-vous ce prestataire à un/e ami(e) ou un membre de votre famille ?** | | Oui 1  Non 0  Pas de réponse -99 | | | | | | | | | |  | |
| 39 | | **Au cours des 12 derniers mois, avez-vous payé des frais pour des services de planification familiale (y compris pour votre méthode actuelle/ méthode la plus récente) ?** | | Oui 1  Non 0 | | | | | | | | | | Aller à 41 si NON | |
| 40 | | **Combien avez-vous payé ?**  *Saisir tous les prix en FRANCS CONGOLAIS. Saisir -88 si ne sait pas. Saisir -99 si pas de réponse.* | | \| Montant total des frais \|  \| \| --- \| --- \| | | | | | | | | | |  | |
| VST_1 | | | **Où avez-vous obtenu [METHODE LA PLUS RECENTE / METHODE ACTUELLE] la dernière fois?**  *Descendre jusqu’en bas pour voir toutes les réponses possibles* | **SECTEUR PUBLIC:**  Centre hospitalier national ..……………….11  Clinique de Planning familiale ………………. 12  Centre/Poste de santé…………………13  Maternité……………………………………14  Relais communautaire …………15  Centre hospitalier régional …………………16  **SECTEUR MEDICAL PRIVE:**  Hôpital/Clinique privé…………………………..…. 21  Pharmacie…………………………….……………………22  ONG …………………….…………………….23  Centre de santé privé………………….…………………24  Cabinet médical privé…………………….………………25  Médecin privé…………………….…………………….26  Infirmier ambulant………………….…………………….27  Relais communautaire / agent de santé communautaire (ASC)……….28  Etudiants – agents de santé communautaire …………………………………………….29  **AUTRES SOURCES:**  Boutique……………………………………….………………31  Institution religieuse ……………….……………….32  Ami(e)/Parent(e) ……………………………………..33  Bar/Boîte de nuit…………………….…………………….34  LIGABLO/Kiosk…………………….………………35  Chayeur……………………………………………………..36  Autre.. ……………………………………………………. 37  Pas de réponse -99 | | | | | | | | | |  | |
| 41 | | **Avez-vous déjà utilisé quelque chose ou essayé par divers moyens de retarder ou d'éviter une grossesse ?** | | Oui 1  Non 0  Pas de réponse -99 | | | | | | | | | | Aller à 43 si NON | |
| 41b | | **Quel âge aviez-vous lorsque vous avez essayé une méthode vous permettant de retarder ou éviter une grossesse  pour la première fois?**  **La participante a dit qu’elle avait [âge à QF1] ans à son dernière anniversaire.**  *Saisir l’âge en années. Saisir -88 si la participante ne sait pas. Saisir -99 s’il n’y a pas de réponse. L'âge indiqué ne peut pas être inférieur à 9 ans.* | | Age | | | |  | | | | | |  | |
| 41c | | **Combien d’enfants en vie aviez-vous à ce moment-là ?**  **Notez: L’enquêtée a indiqué qu’elle avait donné naissance [nombre des naissances vivantes] fois à QF8.**  *Saisir -99 si pas de réponse* | | Nombre | | | |  | | | | | |  | |
| 42 | | **Quelle méthode avez-vous utilisé pour retarder ou éviter une grossesse la première fois?**  *Ne lisez pas les réponses. Descendre jusqu’en bas pour voir tous les choix possibles* | | Stérilisation féminine  Stérilisation masculine  Implants  DIU/Stérilet  Injectable  Pilule  Pilule du lendemain  Préservatif masculin  Préservatif féminin  Diaphragme  Mousse/Gelée spermicide  Méthode des jours fixes/Collier du cycle  MAMA  Méthode du rythme  Retrait  Autres méthodes traditionnelles Pas de réponse -99  Pas de réponse -99 | | | | 1  2  3  4  5  7  8  9  10  11  12  13  14  30  31  39  -99 | | | | | | Si l’Injectable est sélectionnée, aller à 42a | |
| 42a | | **RELANCER :** Est-ce que l’injection a été administrée par seringue ou par petite aiguille ?  *Veuillez montrer l’image à l’enquêtée.*  [ODK AFFICHERA UNE IMAGE DE LA METHODE SUR L’ECRAN] | | Seringue 1  Petite aiguille (SayanaPress) 2  Les deux 3  Pas de réponse -99 | | | | | | | | | |  | |
|  | | **CONTROLE 16:** Souhaite avoir des enfants plus tard?  **CONTROLE 17**: 2 ans ou plus avant le prochain enfant ?  **CONTROLE 20:** Utilise actuellement une méthode de contraception ?  **CONTROLE 41:** A déjà utilisé une méthode contraceptive? | | Avoir un autre enfant 1  Ne plus en avoir 2  Ne peut plus concevoir 3  Indécise/NSP -88 | | | | | | | | | | Poser Q. 43 aux non-usagers (actuellement ou en général) qui ne souhaitent pas d’enfant ou qui ne souhaite pas en avoir avant au moins 2 ans. | |
|  |  |  |  | Ne plus avoir d’enfant 1  Moins de 2 ans 2  2 ans ou plus 3 | | | | | | | | | |  |  |
|  |  |  |  | Oui, utilise un contraceptif 1  Non, n’utilise pas de contraceptif 0 | | | | | | | | | |  |  |
|  |  |  |  | Oui 1  Non 0 | | | | | | | | | |  |  |
| 43 | | **Vous avez dit que vous ne souhaitiez pas/plus avoir d’enfant et que vous n’utilisez pas de méthode contraceptive.**  Pouvez-vous me dire pourquoi vous n'utilisez pas une méthode?  SELECTIONNER TOUTES LES RESPONSES MENTIONNEES.  *Vous ne pouvez ni sélectionner “Ne sait pas” ni “Pas de réponse” avec des autres réponses.*  *Vous ne pouvez pas sélectionner “Pas en union actuellement” si QF3 est “Oui,actuellement mariée”.*  *Descendre jusqu’en bas pour voir toutes les réponses possibles.* | | Pas mariée 1  Peu/pas de rapports sexuels 2  Ménopause/Hystérectomie 3  Infertile / peu fertile 4  N’a pas eu de règles depuis la dernière naissance 5  Allaitement 6  Conjoint absent depuis plusieurs  jours 7  S’en remet à Dieu / Fataliste 8  Enquêtée opposée 9  Mari/conjoint opposée 10  Autres personnes opposées 11  Prohibition religieuse 12  Ne connaît aucune méthode 13  Ne connaît pas où se procurer 14  Peur des effets secondaires 15  Préoccupations de santé 16  Manque d’accès/trop loin 17  Trop cher 18  Méthode préférée non disponible 19  Aucune méthode disponible 20  Peu pratique à utiliser 21  Interfère avec les processus du  corps 22  Prend trop de temps à coté de ses tâches quotidiennes/ Trop occupée pour aller en obtenir………….23  Intention d’en utiliser une, mais n’a pas encore eu la chance de se rendre à la clinique……………………………… 24  Belle-mère opposée……………..25  Autre 36  Ne sait pas -88  Pas de réponse -99 | | | | | | | | | |  | |
| 44 | | **Au cours des 12 derniers mois, est-ce que vous avez reçu la visite d’un agent de santé qui vous avez parlé de planification familiale ?** | | Oui 1  Non 0  Pas de réponse -99 | | | | | | | | | |  | |
| 44a | | **Au cours des 12 derniers mois, avez-vous participé à une causerie sur la planification familiale au niveau communautaire?** | | Oui 1  Non 0  Pas de réponse -99 | | | | | | | | | |  | |
| 45 | | **Au cours des 12 derniers mois, êtes-vous allée dans un établissement de santé pour recevoir des soins pour vous-même ou pour vos enfants?**  *Cela peut être pour n’importe quel service* | | Oui 1  Non 0  Pas de réponse -99 | | | | | | | | | | Aller à 47 si NON | |
| 46 | | **Est ce qu’un membre de l’établissement de santé vous a parlé de méthodes de planification familiale ?** | | Oui 1  Non 0  Pas de réponse -99 | | | | | | | | | |  | |
| 47 | | **Au cours des derniers mois, avez-vous :  Entendu parler du planning familial à la radio ?  Vu quelque chose sur le planning familial à la télévision?  Lu quelque chose à propos du planning familial dans un magazine ou dans les journaux?** | |  | | Oui  1  1  1 | | | Non  0  0  0 | | | | |  | |
| 47b | | **Avez-vous déjà vu cette image ?**  *Veuillez montrer l’image à l’enquêtée.*  **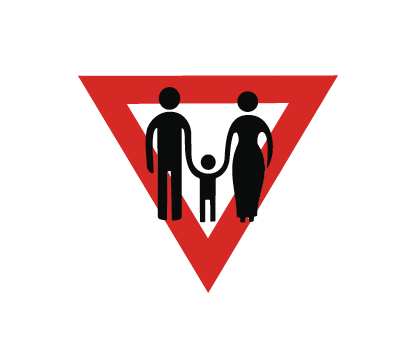** | | Oui 1  Non 0  Ne sait pas/ Ne se souvient pas .-88  Pas de réponse -99 | | | | | | | | | | Aller à 47d si 0,  -88 ou  -99 | |
| 47b1 | | **Où avez-vous vu cette image ?**  **RELANCER : Un autre endroit ?**  *Veuillez sélectionner toutes les réponses mentionnées.* | | Dans une centre de santé 1  Dans la rue 2  Dans une pharmacie 3  Sur un panneau d'affichage ………. 4  A la télévision………………………..5  Sur un dépliant ou prospectus……………………………6  Sur la veste d’un agent de santé communautaire………………………7  Autre 8  Ne sait pas/ Ne se souvient pas -88  Pas de réponse -99 | | | | | | | | | |  | |
| 47c | | **Qu’est-ce que cette image signifie pour vous ?**  *Veuillez sélectionner toutes les réponses mentionnées.* | | La planification familiale 1  L’espacement des naissances 2  La santé de la reproduction 3  La famille 4  Autre 5  Ne sait pas / Ne se souvient pas -88  Pas de réponse -99 | | | | | | | | | |  | |

| 47d | **Avez-vous vu le panneau d'affichage qui indique « Combien d'enfants souhaitez-vous ? » et qui montre le logo de planification familiale?**  *Veuillez montrer l’image à l’enquêtée.*  **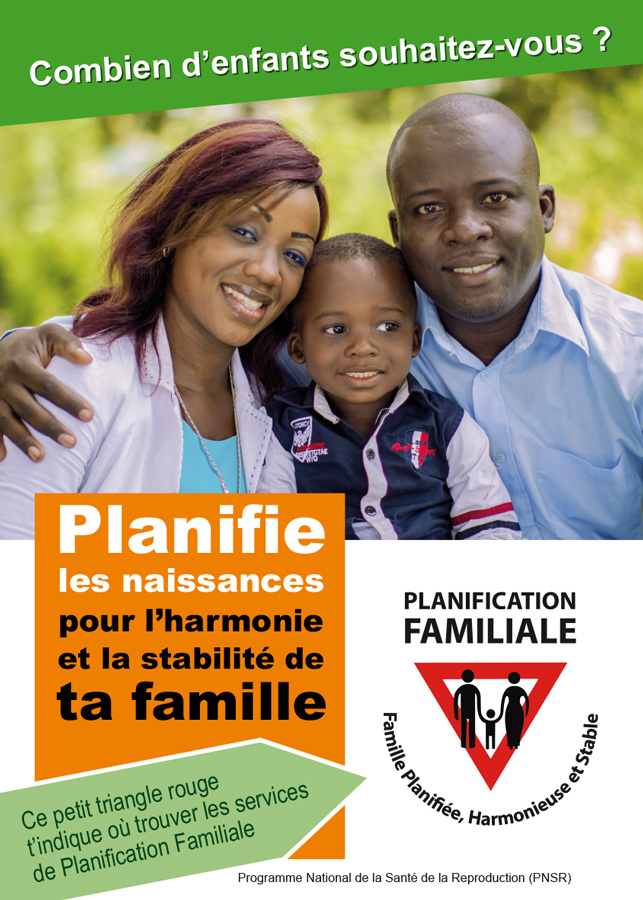** | Oui 1  Non 0  Ne sait pas/ Ne se souvient pas….-88  Pas de réponse -99 | Aller à 48 si 0, -88 ou -99 |
| --- | --- | --- | --- |
| 47e | **Qu’est-ce que cette image signifie pour vous ?**  *Veuillez sélectionner toutes les réponses mentionnées.* | La planification familiale 1  L’espacement des naissances 2  La santé de la reproduction 3  La famille 4  Autre 5  Ne sait pas / Ne se souvient pas -88  Pas de réponse -99 |  |
|  | **VERIFIEZ LA PRESENCE D'AUTRES PERSONNES AVANT DE CONTINUER. FAITES TOUS LES EFFORTS POUR ASSURER L'ISOLEMENT DE L'ECOUTE DES AUTRES.**  *Préparez verbalement l’enquêtée pour les questions sur l’activité sexuelle.* | |  |
| 48 | **Quel âge aviez-vous quand vous avez eu des rapports sexuels pour la première fois ?**  *Saisir l’âge en années.*  *La participante a dit qu’elle avait [âge de QF1] ans à son dernier anniversaire.*  *[Elle avait X naissances vivantes] Saisir -77 si elle n’a jamais eu de rapport sexuel. Saisir -88 si ne sait pas. Saisir -99 si pas de réponse* | \| Age \|  \| \| --- \| --- \| | Aller à MM_1 si -77 |
|  | **Si l’âge lors du premier rapport sexuel est <10 :**  **CONTROLE:**  Vous avez noté que l’enquêtée avait X ans lors de son premier rapport sexuel. Est-ce ce qu’elle a dit ?  **SI NON, RETOURNEZ A QF48 ET CORRIGEZ** | Oui 1  Non 0 |  |
| 49 | **Quand avez-vous eu des rapports sexuels pour la dernière fois ?**  *Si cela fait moins de 12 mois, la réponse doit être indiquée en mois, semaines, ou jours.*  *Saisissez 0 pour aujourd'hui.*  *Vous saisirez le nombre à l'écran suivant.* | Il y a ______ jours  Il y a ______ semaines  Il y a _______ mois  Il y a _______ années |  |
| **Section 4 – Exposition aux médias de masse**  *Maintenant, je voudrais vous poser des questions sur l’exposition aux informations sur la planification familiale à travers les médias de masse.* | | | |
| MM_1 | **Avez-vous regardé l’émission télévisée qui s’appelle ‘LibalaYa Bosembo’ durant les 6 derniers mois ?** | Oui 1  Non 0  Pas de réponse -99 | Aller à MM_6 si 0 ou -99 |
| MM_2 | **Avant la fin de chaque émission libella ya bosembo un message destiné aux couples est donné. La dernière fois que vous avez regardé cette émission, quel (s) était (ent) le(s) message(s) qui vous a (ont) marquée le plus ?**  **RELANCER : Autres choses ?**  *Veuillez sélectionner toutes les réponses mentionnées.* | Communication au sein du couple…………………...………………1  Harmonie au sein de la famille 2  Méthodes de planification familiale… 3  Soins prénataux 4  Soins pour la mère et santé de l’enfant durant la période postpartum 5  Aménorrhée après grossesse 6  Espacement des naissances 7  Allaitement 8  Autres 9  Ne sait pas / Ne se souvient pas -88  Pas de réponse -99 |  |
| MM_3 | **Ont-ils parlé des méthodes de planification familiale au cours de l’émission ‘Libala Ya Bosembo’ ?** | Oui 1  Non 0  Ne sait pas / Ne se souvient pas -88  Pas de réponse -99 | Aller à MM_6 si 0, -88 ou -99 |
| MM_4 | **Avez-vous parlé à quelqu’un des messages de planification familiale de l’émission télévisée ‘Libala Ya Bosembo’ ?** | Oui 1  Non 0  Ne sait pas / Ne se souvient pas -88  Pas de réponse -99 | Aller à MM_6 si 0, -88, ou -99 |
| MM_5 | **A qui en avez-vous parlé ?**  **RELANCER : Quelqu’un d’autre ?**  *Veuillez sélectionner toutes les réponses mentionnées* | Prestataire de santé 1  Conjoint 2  Autres membres de la famille 3  Amies (Amis) / Voisines (Voisins) 4  Pharmacien/Aide du pharmacien 5  Agent de sensibilisation 6  Des gens lors d’une séminaire/ réunion communautaire 7  Autres 8  Ne sait pas / Ne se souvient pas -88  Pas de réponse -99 |  |
| MM_6 | **Avez-vous déjà regardé les** **petits films télévisés dénommés ‘Elengi’ ?** | Oui 1  Non 0  Pas de réponse -99 | Aller à MM_8 si -0 ou -99 |
| MM_7 | **La dernière fois vous avez regardé le petit film dénommé ‘Elengi’, de quoi s’agissait-il ?**  **RELANCER : Autres choses ?**  *Veuillez sélectionner toutes les réponses mentionnées* | Communication au sein du couple…………………...………………1  Harmonie au sein de la famille ………2  Méthodes de la planification familiale. 3  Soins prénataux ………………………4  Soins pour la mère et santé de l’enfant durant la période postpartum …………5  Aménorrhée après grossesse 6  Espacement des naissances 7  Allaitement 8  Autres 9  Ne sait pas / Ne se souvient pas 10  Pas de réponse -99 |  |
| MM_8 | **Avez-vous déjà appelé le numéro vert ‘3-2-1’ ?** | Oui 1  Non 0  Pas de réponse -99 | Aller à SE_1 si 0 ou -99 |
| MM_9 | **La dernière fois vous avez appelé le numéro vert ‘3-2-1’, de quelles informations aviez-vous besoin ?**  **RELANCER : Autres choses ?**  *Veuillez sélectionner toutes les réponses mentionnées* | Où se trouvent des services de planification familiale 1  Information sur les méthodes de planification familiale 2  Effets secondaires des méthodes de planification familiale 3  Soins après avortement 4  Soins après violence du partenaire intime 5  VIH/SIDA 6  Santé en général 7  Autre 8  Ne sait pas / Ne se souvient pas -88  Pas de réponse -99 |  |

|  | **Section 5 –Auto-efficacité perçue dans l’utilisation des méthodes contraceptives**  *Maintenant je voudrais vous poser des questions sur votre capacité à poser avec confiance certaines actions en rapport avec l’utilisation des méthodes de planification familiale. Si vous n’êtes pas actuellement mariée ou n’a pas un partenaire habituel, essayez d’imaginer comment vous répondriez si étiez en union.* | | | | | | |
| --- | --- | --- | --- | --- | --- | --- | --- |
|  | | Dans quelle mesure êtes-vous confiante que : | *Très confiante* | *Confiante* | *Un peu confiante* | *Pas confiante* | *Pas du tout confiante* |
| SE_1 | | Vous pouvez entamer une conversation avec votre mari/conjoint/partenaire sur la planification familiale/l'espacement des naissances ? | 5 | 4 | 3 | 2 | 1 |
| SE_2 | | Vous pouvez convaincre votre mari/conjoint/partenaire d’utiliser une méthode de planification familiale/d’espacement des naissances ? | 5 | 4 | 3 | 2 | 1 |
| SE_3 | | Vous pouvez vous rendre à un établissement où des méthodes de planification familiale/d’espacement des naissances sont offertes si vous décidiez d’en utiliser une ? | 5 | 4 | 3 | 2 | 1 |
| SE_4 | | Vous pouvez obtenir une méthode de planification familiale/d’espacement des naissances si vous décidiez d’en utiliser une ? | 5 | 4 | 3 | 2 | 1 |
| SE_5 | | Vous pouvez utiliser une méthode de planification familiale/d'espacement des naissances, même si votre mari/conjoint ne le souhaite pas ? | 5 | 4 | 3 | 2 | 1 |
| SE_6 | | Vous pouvez utiliser une méthode de planification familiale/d'espacement des naissances, même si aucun(e) de vos ami(e)s ou de vos voisin(e)s n’en utilise ? | 5 | 4 | 3 | 2 | 1 |
| SE_7 | | Vous pouvez utiliser une méthode de planification familiale/d'espacement des naissances, même si votre chef religieux ne pense pas que vous devriez en utiliser ? | 5 | 4 | 3 | 2 | 1 |
| SE_8 | | Vous pouvez continuer à utiliser une méthode de planification familiale/d’espacement des naissances, même si vous ressentez des effets secondaires ? | 5 | 4 | 3 | 2 | 1 |

| **Section 6 – Les maladies diarrhéiques chez les enfants**  *Maintenant, je voudrais vous poser quelques questions sur la santé des enfants de moins de 5 ans.* | | | | | | |
| --- | --- | --- | --- | --- | --- | --- |
| 50 | | | Pour combien d'enfants de moins de 5 ans êtes-vous la mère ou nourrice ?  *Saisissez 0 si vous n’en avez pas. Saisissez -99 pour pas de réponse.* | \| Nombre \|  \| \| --- \| --- \| | | Aller à K si 0  Ninon, aller à QF51 |
|  | | | Commençant par l’enfant le plus jeune, je voudrais vous poser quelques questions.  **[Les questions QF51-53 seront répétées pour chaque enfant âgés de moins de 5 ans].** |  | |  |
| 51 | | | En quel mois et quelle année est né l’enfant ?  ***Saisissez « Jan 2020 » pour pas de réponse.*** | \| Mois \|  \| \| --- \| --- \| \| Année \|  \| | |  |
| 52 | | | La dernière fois que cet enfant a fait ses besoins, qu’avez vous fait/ ou qu’est que l’enfant a fait avec des déchets ?  Relancer : Pour tout type de selles, soit normales ou diarrhéiques.  L’enfant a utilisé des latrines / toilettes  Déchets laissés là où ils étaient  Déchets enterré dans un champ / jardin  Déchets jetés dans des latrines/ toilettes  Déchets jetés avec les autres ordures  Déchets jetés avec les eaux usagées  Déchets utilisés comme fumier  Déchets brûlés  Ne sait pas  Pas de réponse | Oui  1  1  1  1  1  1  1  1  -88  -99 | Non  0  0  0  0  0  0  0  0 |  |
| 53 | | | Est-ce que cet enfant a eu la diarrhée au cours des 7 derniers jours?  ***La diarrhée est déterminée par la répondante. Si l’enquêtée n’est pas familière avec le terme « diarrhée, » veuillez lui indiquer que cela signifie « au moins trois selles liquides par jour. »*** | Oui 1  Non 0  Pas de réponse -99 | |  |
| 54 | | | Qui dans votre famille fait partie des Forces Armées de la RDC? | 1) Moi-même  2) Mon époux / épouse  3) Mon fils / Mes fils  4) Ma fille / Mes filles  5) Autres (précisez): _____ | |  |
| 55 | | | Au total, combien de membre de votre famille immédiate (époux, enfants) font partie des FARDC? | Combien____________________ | |  |
| 56 | | | Quel est votre / son rang au sein de l’armée? | \| **1** \| Soldat 2^ème^ classe \| \| --- \| --- \| \| **2** \| Soldat 1 ^ère^ classe \| \| **3** \| Caporal \| \| **4** \| Sergent \| \| **5** \| Sergent major \| \| **6** \| 1 ^er^ sergent \| \| **7** \| 1 ^er^ Sergent Adjoint \| \| **8** \| Adjudant de 2^ème^ classe \| \| **9** \| Adjudant \| \| **10** \| Adjudant en chef \| \| **11** \| Sous-Lieutenant \| \| **12** \| Lieutenant \| \| **13** \| Capitaine \| \| **14** \| Major \| \| **15** \| Lieutenant Colonel \| \| **16** \| Colonel \| \| **17** \| General de brigades \| \| **18** \| Lieutenant General \| \| **19** \| General des corps d’armée \| \| **20** \| General d’armée \| | |  |
| 57 | | | Avez-vous déjà vu un panneau ou une banderole parlant de planification familiale qui montre une famille de militaires? | 0 Non  1 Oui  88 Ne sait pas | |  |
| **Remerciez la personne interrogée pour le temps qu’elle a bien voulu vous accorder**  *L’enquêtée a terminé, mais il vous reste d’autres questions à compléter en dehors de la maison* | | | | | | |
| **LOCALISATION** | | | | | | |
| K | | | **Géoréférenciation**  *Veuillez faire une mesure des coordonnées GPS prêt de l’entrée de la maison. Saisir les coordonnées quand leur exactitude est inférieure à 6m.* | SAISIR LES COORDONNÉES GPS. | |  |
| **RESULTAT DU QUESTIONNAIRE** | | | | | | |
| L | | **Combien de fois avez-vous rendu visite à ce ménage pour interroger cette femme?** | 1^ère^ fois 1  2^ème^ fois 2  3^ème^ fois 3 | |  |  |
| M | **Résultat du questionnaire**  *Sélectionnez le résultat du Questionnaire femme.* | Complété 1  Pas à la maison 2  Différé 3  Refusé 4  Complété en partie 5  Incapacité 6 | |  |  |  |
