# Supplementary material for: The gap in contraceptive knowledge and use between the military and non-military populations of Kinshasa, DRC, 2016–2019
Source: PLoS One. 2021 Jul 27;16(7):e0254915. doi: 10.1371/journal.pone.0254915 (PMC8315532; doi:10.1371/journal.pone.0254915)
Supplement: S3 File — (DOCX) [file pone.0254915.s003.docx]

**S3 File. 2020 military survey questionnaire, English**

**Section A: Identification**

| **NO** | **QUESTION** | **RESPONSES** | | **SKIP** |
| --- | --- | --- | --- | --- |
| A1 | **Are you in the correct household?**  **This is the picture of the front of the home taken during the Household Questionnaire.**  **IF NOT, RETURN TO INTERVIEW THE CORRECT HOUSEHOLD.**  [ODK will display the photo attached to the linked Household Questionnaire] | Yes  No | 1  0 |  |
| A2 | **Your name:** [Interviewer name from **Female Questionnaire**]  **Is this your name?** | Yes  No | 1  0 | If “yes,” skip to A4. |
| A3 | **Enter your name.**  *Please record your name* |  |  |  |
| A4 | **Current date and time.** [ODK will display on screen]  **Is this date and time correct?** | Yes  No | 1  0 | If “yes,” skip to A6. |
| A5 | **Record the correct date and time.** |  |  |  |
| A6 | **The following information is from the Female Questionnaire. Please review to make sure you are interviewing the correct respondent.**  [ODK will display the province, city, commune, and quartier for Kinshasa EAs and the province, district, *aire de santé*, and village for Kongo Central EAs. In addition, the Enumeration Area, Structure Number, and Household Number entered into the Household Questionnaire linked to this Female Questionnaire will be displayed.]  **Is the above information correct?** | Yes  No | 1  0 |  |
| A7 | **CHECK: You should be attempting to interview [Respondent’s Name]. Is that correct?**  *If this is the wrong person, you have two options:*  *(1) exit and ignore changes to this form. Open the correct form.*  *Or*  *(2) find and interview the person whose name appears above.* | Yes  No | 1  0 |  |
| A8 | **Is the respondent present and available to be interviewed today?** | Yes  No | 1  0 | If “no,” skip to H1. |

**Section B: Informed consent**

*Trouvez la femme entre 15 et 49 ans qui est associée avec un Questionnaire de Suivi Femme. La femme doit être mariée ou vivre en union.*

*L’enquête doit avoir lieu en privé, sans que personne d’autre ne puisse entendre. Merci de lire le texte qui suit après avoir salué la / le répondant(e) :*

*Bonjour. Je m’appelle ________________________________ et je travaille pour l’École de Santé Publique de Kinshasa en collaboration avec le Ministère de la Santé. Nous menons actuellement une enquête à Kinshasa et au Kongo Central sur plusieurs thèmes liés à la santé reproductive. Nous apprécions beaucoup que vous fassiez partie de cette enquête. Les informations que nous collecterons aideront à informer le gouvernement afin de mieux planifier les services de santé. Le questionnaire prend généralement entre 15 et 20 minutes.*

*Toutes les informations que vous nous donnerez seront strictement confidentielles et ne seront partagées avec personne d’autre que les membres de notre équipe. Nous interviewerons également votre partenaire mais nous ne partagerons pas vos réponses avec lui. La participation à cette enquête est volontaire, et s’il y a une question à laquelle vous ne souhaitez pas répondre, faîtes le moi savoir et je passerai à la suivante ; ou vous pouvez également interrompre l’entretien à tout moment. Cependant, nous espérons que vous accepterez de participer à cette enquête car votre point de vue est important.*

*Est-ce que vous souhaitez maintenant me poser des questions concernant cette enquête ? Est-ce que vous êtes d’accord pour participer à cette enquête ?*

| **NO** | **QUESTION** | **RESPONSES** | | **SKIP** |
| --- | --- | --- | --- | --- |
| B1 | *Provide a paper copy of the Consent Form to the respondent and explain it. Then, ask*:  **May I begin the interview now?** | Yes  No | 1  0 | If “no,” skip to H1. |
| B2 | **Respondent’s signature**  *Please ask the respondent to sign or check the box in agreement of their participation.* | Check box:  |  |  |
| B3 | **Interviewer’s name:** [Interviewer name from Household Questionnaire]  *Mark your name as a witness to the consent process.* |  |  |  |
| B4 | **Respondent’s name**  [ODK will display the Respondent’s name from linked Household Roster]  *You may correct the spelling here if it is not correct, but you must be interviewing the person whose name appears below.* |  |  |  |
| B5 | **Was this respondent interviewed in the previous round?** | Yes  No  Don’t know |  |  |

**Section C: Respondent’s background, marital status, and household characteristics**

Now I would like to ask about your background and socioeconomic conditions.

| **NO** | **QUESTION** | **RESPONSES** | | **SKIP** |
| --- | --- | --- | --- | --- |
| C1 | **In what month and year were you born?**  **The age in the household roster is [AGE].** | Month:  Year: |  |  |
| C2 | **How old were you at your last birthday?**  *Must agree with C1.* |  |  | If less than 15, stop the interview and skip to H1. |
| C3 | **What is the highest level of school you attended?** | Never attended  Primary  Secondary  Tertiary  No response | 0  1  2  3  -99 |  |
| C4 | Are you in the military? | Yes No |  | If no, skip to C6. |
| C5 | What is your rank in this service? | Soldier 1st class  Soldier 2nd class  Corporal  Sargent  Sargent major  1st Sargent  1st Sargent Adjoint  Adjudant de 2ème classe  Adjudant  Adjudant in chef  Second Lieutenant  Lieutenant  Captain  Major  Lieutenant Colonel  Colonel  General Brigadier  Lieutenant General  General of Army Corps  General or Army | 1  2  3  4  5  6  7  8  9  10  11  12  13  14  15  16  17  18  19  20 |  |
| C6 | In general, would you say your health is:  *Read responses.* | Excellent  very good  Good  Fair Poor  No response | 1  2  3  4  5  -99 |  |
| C7 | **(Confirm) Are you currently married or living together with a man as if married?** | No  Yes, married  Yes, living together | 0  1  2 | If “no,” stop the interview and skip to H1. |
| C8 | **Have you been married or lived with a man only once or more than once?** | Only once  More than once  No response | 1  2  -99 | If “only once,” skip to C11. |
| C9 | **In what month and year did you start living with your FIRST husband / partner?**  *Enter Jan 2020 for no response.* | Month:  Year: |  |  |
| C10 | [If ≤15 years old at marriage date ODK will display:]  **CHECK: Based on the response you entered in C7, the respondent was possibly 15 years old or younger at the time of her first marriage. Did you enter C7 correctly?** | Yes  No | 1  0 |  |
| C11 | **Now I would like to ask about when you started living with your CURRENT husband/ partner. In what month and year was that?**  *Enter Jan 2020 for no response.* | Month:  Year: |  |  |
| C12 | [If ≤15 years old at marriage date ODK will display:]  **CHECK: Based on the response you entered in C9, the respondent was possibly 15 years old or younger at the time of her first marriage. Did you enter C11 correctly?** | Yes  No | 1  0 |  |
| C13 | **Does your husband / partner have other wives, or does he live with other women as if married?** | Yes  No  Don’t know  No response | 1  0  -88  -99 |  |
| C14 | **Is your husband / partner living with you now or is he staying elsewhere?** | Living with respondent  Staying elsewhere  No response | 1  2  -99 |  |

**Section D: Reproduction and Fertility Preferences**

Now I would like to ask about all the births that you have had during your life.

| **NO** | **QUESTION** | **RESPONSES** | | **SKIP** |
| --- | --- | --- | --- | --- |
| D1 | **How many times have you been pregnant?** |  |  |  |
| D2 | **How many times have you given birth?**  *Zero is a possible answer.* | Number  No response | #  -99 |  |
| D2 | **Were all of those live births?**  *If no, go back and change D2 to record only live birth events.* | Yes  No  No response | 1  0  -99 |  |
| D3 | **Have you given birth to a child who was born alive but later died?**  *IF NO, PROBE: Any baby who cried or showed signs of life but did not survive?* | Yes  No  No response | 1  0  -99 | If “no” or “no response,” skip to D5. |
| D4 | **How many have died?** | Number  Don’t know  No response | #  -88  -99 |  |
| D5 | **Just to make sure I have this right: you have had a total of __ births during your life, resulting in __ sons and daughters born alive.**  **Is that correct?** | Yes  No  No response | 1  0  -99 | If “no,” go back and correct D2-D4. |
| D6 | **Have you adopted or do you have any children who are living with you instead of with their biological parents?** | Yes  No | 1  0 | If “no,” skip to D8. |
| D7 | **How many of these children have you adopted/live with you?** |  |  |  |
| D8 | **When was your FIRST birth?**  *Please record the date of the first live birth. Date should be found by calculating forward or backward from memorable events if needed. Enter Jan 2020 for no response.* | Month  Year |  |  |
| D9 | **When was your MOST RECENT birth?**  *Please record the date of the MOST RECENT live birth. The date should be found by calculating backwards from memorable events if needed.*  *Enter Jan 2020 for no response.* | Month  Year |  | If not in last year, and/or D5=1, skip to D11. |
| D10 | **When did you give birth before the most recent one?**  *Please record the date of the birth before the last. The date should be found by calculating backwards from memorable events if needed.*  *Enter Jan 2020 for no response.* | Month  Year |  | If D5 = 1, skip to D11 |
| D11 | **Is your last baby / child still alive?** | Yes  No  Don’t know  No response | 1  0  -88  -99 | If “yes,” “don’t know,” or “no response,” skip to D13. |
| D12 | **When did your last baby / child die?**  *Please record the date of the child’s death.*  *The date should be found by calculating backwards from memorable events if needed.*  *Enter Jan 2020 for no response.* | Month  Year |  |  |
| D13 | **When did your last menstrual period start?**  *If you select days, weeks, months or years, you will enter a number for x on the next screen.*  *Enter 0 days for today, not 0 weeks/months/years.* | _ days ago  _ weeks ago  _months ago  _years ago  Menopausal/hysterectomy  Before last birth  Never menstruated  No response | 5  6  7  -99 |  |
| D13a | **From one menstrual period to the next, are there certain days when a woman is more likely to become pregnant?** | Yes  No  Don’t know  No response | 1  2  3  -99 | If no or no response, skip to D14 |
| D13b | **Is this time just before her period begins, during her period, right after her period has ended, or halfway between two periods?** | Just before her period beings  Right after her period has ended  Halfway between two periods  Other  Don’t know  No response | 1  2  3  4  -98  -99 |  |
| D14 | **Are you pregnant now?** | Yes  No  Don’t know  No response | 1  0  -88  -99 |  |
| D15 | **How many months pregnant are you?**  *Record the number of completed months.* | Number  Don’t know  No response | 1-9  -88  -99 |  |
|  | *CHECK D14: Currently pregnant?* |  |  | If not currently pregnant, proceed to D16.  If currently pregnant, skip to D19. |
| D16a | **Now I would like to ask a question about your last birth.**  **At the time you became pregnant, did you want to become pregnant then, did you want to wait until later, or did you not want to have any / any more children at all?** | Then  Later  Not at all  No response | 1  2  3  -99 |  |
| D16b | **At the time you became pregnant, did your partner want you to become pregnant then, did he want to wait until later, or did he not want to have any / any more children at all?** | Then  Later  Not at all  Don’t know  No response | 1  2  3  -98  -99 |  |
| D17a | **Now I have some questions about the future. Would you like to have a/another child, or would you prefer not to have any / any more children?** | Have a/another child  No more  Couple is infertile  Undecided/DK  No response | 1  2  3  -88  -99 | If “have another child,” proceed to D18.  If any other response, skip to E1. |
| D17b | **How long would you like to wait from now before the birth of a/another child?**  *If you select months or years, you will enter a number for x on the next screen.*  *Select “Years” if more than 36 months.* | Months:  Years:  Soon/now  Couple is infertile  Other  Don’t know  No response | #  #  1  2  3  -88  -99 | If “couple is infertile,” skip to E15. Otherwise, skip to D22. |
| D18a | **Would your partner like to have a/another child, or would he prefer not to have any / any more children?** | Have a/another child  No more  Couple is infertile  Undecided/DK  No response | 1  2  3  -88  -99 | If “have another child,” proceed to D18.  If any other response, skip to E1. |
| D18b | **How long would your partner like to wait from now before the birth of a/another child?**  *If you select months or years, you will enter a number for x on the next screen.*  *Select “Years” if more than 36 months.* | Months:  Years:  Soon/now  Couple is infertile  Other  Don’t know  No response | #  #  1  2  3  -88  -99 | If “couple is infertile,” skip to E15. Otherwise, skip to D22. |
| D19 | **Now I would like to ask a question about your current pregnancy.**  **At the time you became pregnant, did you want to become pregnant then, did you want to wait until later, or did you not want to have any / any more children at all?** | Then  Later  Not at all  No response | 1  2  3  -99 |  |
| D20 | **Now I have some questions about the future. After the child you are expecting now, would you like to have another child, or would you prefer not to have any more children?** | Have a/another child  No more  Couple is infertile  Undecided/DK  No response | 1  2  3  -88  -99 | If NOT “have another child,” skip to E1. |
| D21 | **After the birth of the child you are expecting now, how long would you like to wait before the birth of another child?**  *If you select months or years, you will enter a number for x on the next screen.*  *Select “Years” if more than 36 months.* | Months:  Years:  Soon/now  Couple is infertile  Other  Don’t know  No response | #  #  1  2  3  -88  -99 | If “couple is infertile,” skip to E1. |
| D22 | **In total, how many more children would you like to have?** | Number  Undecided/DK  No response | # -88  -99 |  |

**Section E. Contraception**

**Now I would like to talk about family planning - the various ways or methods that a couple can use to delay or avoid a pregnancy.**

An image will appear on the screen for some methods. If the respondent says that she has not heard of the method or if she hesitates to answer, read the probe aloud and show her the image, if available.

| **#** | **Question & probe** | **Instructions** | **Response set** | **Relevance** |
| --- | --- | --- | --- | --- |
| **Ask of everyone.** | | | | |
| E1 | Have you ever heard of female sterilization?  PROBE: Women can have an operation to avoid having any more children. |  | yes_no_nr_list |  |
| E2 | Have you ever heard of male sterilization?  PROBE: Men can have an operation to avoid having any more children. |  | yes_no_nr_list |  |
| E3 | Have you ever heard of the contraceptive implant?  PROBE: Women can have one or several small rods placed in their upper arm by a doctor or nurse, which can prevent pregnancy for one or more years. | [IMAGE OF METHOD WILL APPEAR ON SCREEN] | yes_no_nr_list |  |
| E4 | Have you ever heard of the IUD?  PROBE: Women can have a loop or coil placed inside them by a doctor or a nurse. | [IMAGE OF METHOD WILL APPEAR ON SCREEN] | yes_no_nr_list |  |
| E5 | Have you ever heard of injectables?  PROBE: Women can have an injection by a health provider that stops them from becoming pregnant for one or more months. | [IMAGE OF SAYANA PRESS AND DEPO PROVERA WILL APPEAR ON SCREEN] | yes_no_nr_list |  |
| E6 | Have you ever heard of the (birth control) pill?  PROBE: Women can take a pill every day to avoid becoming pregnant. | [IMAGE OF METHOD WILL APPEAR ON SCREEN] | yes_no_nr_list |  |
| E7 | Have you ever heard of emergency contraception?  PROBE: As an emergency measure after unprotected sexual intercourse women can take special pills at any time within five days to prevent pregnancy. |  | yes_no_nr_list |  |
| E8 | Have you ever heard of condoms?  PROBE: Men can put a rubber sheath on their penis before sexual intercourse. | [IMAGE OF METHOD WILL APPEAR ON SCREEN] | yes_no_nr_list |  |
| E9 | Have you ever heard of female condoms?  PROBE: Women can put a sheath in their vagina before sexual intercourse. | [IMAGE OF METHOD WILL APPEAR ON SCREEN] | yes_no_nr_list |  |
| E10 | Have you ever heard of the diaphragm?  PROBE: Women can place a thin flexible disk in their vagina before sexual intercourse. | [IMAGE OF METHOD WILL APPEAR ON SCREEN] | yes_no_nr_list |  |
| E11 | Have you ever heard of foam or jelly as a contraceptive method?  PROBE: Women can place a suppository, jelly, or cream in their vagina before sexual intercourse to prevent pregnancy. | [IMAGE OF METHOD WILL APPEAR ON SCREEN] | yes_no_nr_list |  |
| E12 | Have you ever heard of the standard days method or Cycle Beads?  PROBE: A woman can use a string of colored beads to know the days she can get pregnant. On the days she can get pregnant, she and her partner use a condom or do not have sexual intercourse. | [IMAGE OF METHOD WILL APPEAR ON SCREEN] | yes_no_nr_list |  |
| E13 | Have you ever heard of the Lactational Amenorrhea Method or LAM? |  | yes_no_nr_list |  |
| E14 | Have you ever heard of the rhythm method?  PROBE: Women can avoid pregnancy by not having sexual intercourse on the days of the month they think they can get pregnant. |  | yes_no_nr_list |  |
| E15 | Have you ever heard of the withdrawal method?  PROBE: Men can be careful and pull out before climax. |  | yes_no_nr_list |  |
| E16 | Have you ever heard of any other ways or methods that women or men can use to avoid pregnancy? |  | yes_no_nr_list |  |
| E17 | Specify other ways. |  | text | if E16=yes |
| **Ask of those who knew at least one method.** | | | | |
| E18 | Have you ever done something or used any method to delay or avoid getting pregnant? |  | yes_no_nr_list |  |
| E19 | What method or methods have you used? Probe: Anything else? | Select all that apply. | methods_list | if E18=yes |
| E19s | Specify |  | text | if E19=other |
| E20 | In the past 12 months, have you done something or used any method to delay or avoid getting pregnant? |  | yes_no_nr_list |  |
| E21 | What method or methods have you used in the past 12 months? Probe: Anything else? | Select all that apply. | methods_list | if E20=yes |
| E21s | Specify |  | text | if E21=other |
| E22 | Are you currently doing something or using any method to delay or avoid getting pregnant? |  | yes_no_nr_list |  |
| E23 | What method or methods are you using? Probe: Anything else? | Select all that apply. | methods_list | If E22=yes |
| E23s | Specify |  | text | if E23=other |
| **Ask of ever-users.** | | | | |
| E24 | Think about the first time you used a method to delay or prevent getting pregnant? How many living children did you have at that time, if any? |  | numeric | IF E18=yes |
| **Ask of those currently using or used a method in the last 12 months.** | | | | |
| E25 | Before you started using [MOST RECENT / CURRENT METHOD], did you talk with your husband/partner about using a contraceptive method? |  | yes_no_nr_list |  |
| E26 | Does your partner know that you are using a contraceptive method? |  | yes_no_dkdr_nr_list |  |
| E27 | Does your partner approve of you using a contraceptive method? |  | yes_no_nr_list | If E26=yes |
| E28 | Would your partner approve of you using a contraceptive method if he knew? |  | yes_no_nr_list | If E26=no or don’t know |
| E29 | Who decided that you would use contraception (in general) in this relationship? |  | decision_list |  |
| **Ask about each modern method currently using or used in past 12 months (loop).** | | | | |
| E30 | Who decided that you would use X method? |  | decision_list |  |
| E31 | Why did you start using X method? PROBE: Any other reason? | Select all that apply. | start_list |  |
| E31s | Specify |  | text | if E31=other |
| E32 | Is this your preferred method? |  | yes_no_nr_list |  |
| E33 | If no, what is your preferred method? |  | methods_list |  |
| E33s | Specify |  | text | If E33=other |
| E34 | Why are you not using your preferred method? PROBE: Any other reason? | Select all that apply. | nopref_list |  |
| E34s | Specify |  | text | if E34=other |
| E35 | Where did you obtain X method the last time? |  | locations_list |  |
| E35s | Specify |  | text | if E35=other |
| E36 | Did you obtain X method within the military camp last time? |  | yes_no_dkdr_nr_list |  |
| **Ask of all who currently use or have used a modern method in last 12 months.** | | | | |
| E37 | Overall, where would you prefer to obtain your contraceptive method(s)? |  | locations_list |  |
| E37s | Specify |  | text | If E37=other |
| E38 | Why do you prefer to obtain your method from this provider? PROBE: Any other reason? | Select all that apply. | provpref_list |  |
| E38s | Specify |  | text | if E38=other |
| E39 | In the last 12 months, have you and your husband/partner paid any fees for family planning services (including the most current method)? |  | yes_no_dkdr_nr_list |  |
| E40 | In total, how much have you and your husband/partner paid for family planning services in the last 12 months? | Enter amount in Congolese francs. | numeric | If E39=Yes |
| **Ask of all who have ever used withdrawal.** | | | | |
| E41 | Why did you start using withdrawal? PROBE: Any other reason? | Select all that apply. | start_list |  |
| E42 | Specify. |  | text | If E41=Other |
| **Ask of all who ever used withdrawal but not currently or within last 12 months.** | | | | |
| E43 | Why did you stop? PROBE: Any other reason? | Select all that apply | stop_list |  |
| E44 | Specify. |  | text | If E43=Other |
| **Ask if currently using withdrawal or used within the last 12 months.** | | | | |
| E45 | The last time you used withdrawal, who made the decision to use it: you, your husband/partner, or both? |  | decision_list |  |
| E46 | Have you experienced any difficulties using withdrawal? |  | yes_no_nr_list |  |
| E47 | What are the difficulties using withdrawal? |  | text | If E46=Yes |
| E48 | Do you and your partner discuss your fertile period? |  | yes_no_nr_list |  |
| E49 | Do you and your partner use withdrawal during your fertile periods only or every time you have sex? |  | fertile_list |  |
| E50 | Is withdrawal your preferred method to prevent or delay pregnancy? |  | yes_no_nr_list |  |
| E51 | Why or why not? |  | text |  |
| E52 | What method would you prefer to use? |  | methods_list | If E50=No |
| E53 | Why are you not using your preferred method? PROBE: Any other reason? | Select all that apply. | nopref_list | If E50=No |
| E53s | Specify |  | text | If e53=other |
| E54 | In the last three months when you’ve had sex, have you used the withdrawal method every time, sometimes, rarely, or never? |  | freq_list |  |
| E55 | Why have you not used withdrawal every time you’ve had sex in the past three months? |  | text | IF E54!=Every time |
| **Ask of all who have ever used withdrawal.** | | | | |
| E56 | Have you ever gotten pregnant despite using withdrawal? |  | yes_no_dkdr_nr_list |  |
| **Ask of all who have ever used the rhythm method.** | | | | |
| E57 | Why did you start using the rhythm method? PROBE: Any other reason? | Select all that apply. | start_list |  |
| E57s | Specify |  | text | If E57=other |
| **Ask of all who ever used the rhythm method but not currently or within last 12 months.** | | | | |
| E58 | Why did you stop using the rhythm method? PROBE: Any other reason? | Select all that apply. | stop_list |  |
| **Ask if currently using the rhythm method or used within the last 12 months.** | | | | |
| E59 | The last time you used the rhythm method, who made the decision to use it: you, your husband/partner, or both? |  | decision_list |  |
| E60 | Have you experienced any difficulties using the rhythm method? |  | yes_no_nr_list |  |
| E61 | What are the difficulties using the rhythm method? |  | text | If E60=yes |
| E62 | Does your partner know that you use the rhythm method? |  | yes_no_dkdr_nr_list |  |
| E63 | Does your partner agree or disagree with using the rhythm method? |  | yes_no_dkdr_nr_list |  |
| E64 | Do you and your partner discuss the days you need to abstain from sex, or not? |  | yes_no_nr_list |  |
| E65 | How often do you and your partner abstain from sex during your fertile period? Would you say “always, usually, about half the time, seldom, or never?” |  | freq_list |  |
| E66 | Why do you not always abstain during your fertile period? |  | text | if E65!=always |
| E67 | During your fertile days, do you ever use another method of contraception? |  | yes_no_nr_list |  |
| E68 | What contraceptive methods do you use when you have sex during your fertile period? PROBE: Any other methods? | Select all that apply. | methods_list | if E67=yes |
| E68s | Specify |  | text | if E68=other |
| E69 | In the last three months, have you used the rhythm method every month, some months, or hardly at all? |  | freq2_list |  |
| E70 | Why have you not used the rhythm method every month in the past three months? |  | text | If E69!=every month |
| **Ask of all who have ever used the rhythm method.** | | | | |
| E71 | Have you ever gotten pregnant despite using the rhythm method? |  | yes_no_dkdr_nr_list |  |
| A**sk of everyone**. | | | | |
| E72 | Do you think you will use or continue to use a contraceptive method to delay or avoid pregnancy at any time in the future? |  | yes_no_dkdr_nr_list |  |
| E73 | What types of contraception do you think you will use? PROBE: Any other methods? | Select all that apply. | methods_list | if E72=yes |
| E73s | Specify |  | text | if E73=other |
| E74 | In the last 12 months, were you visited by a community health worker who talked to you about family planning? |  | yes_no_dkdr_nr_list |  |
| E75 | In the last 12 months, did you participate in a group talk at the community level about family planning? |  | yes_no_dkdr_nr_list |  |
| E76 | In the last 12 months, have you visited a health facility for care for yourself or your children? |  | yes_no_dkdr_nr_list |  |
| E77 | Did any staff member at the health facility speak to you about family planning methods? |  | yes_no_dkdr_nr_list | if E76=yes |
| E78 | In the last few months have you heard about family planning on the radio? |  | yes_no_dkdr_nr_list |  |
| E79 | In the last few months have you seen anything about family planning on the television? |  | yes_no_dkdr_nr_list |  |
| E80 | In the last few months have you read about family planning in a newspaper or magazine? |  | yes_no_dkdr_nr_list |  |
| E81 | Have you seen this image before? | **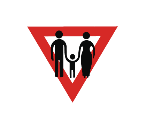** | yes_no_dkdr_nr_list |  |
| E82 | Where did you see this image? PROBE: Any other locations? | Select all that apply. | imageloc_list | If E81=yes |
| E82s | Specify |  | text | If E82=other |
| E83 | What is this image trying to say? Probe: Any other messages? | Select all that apply. | message_list |  |
| E83s | Specify |  | text | if E83=other |
| E84 | Have you seen this billboard or a similar one (showing a military family)? | **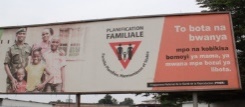** | yes_no_dkdr_nr_list |  |
| E85 | Have you ever seen an activity in your community called “Lelo FP?” |  | yes_no_dkdr_nr_list |  |
| E86 | Have you ever received a family planning method from a Lelo FP event? |  | yes_no_dkdr_nr_list | if E85=yes |
| **Ask based on current methods.** | | | | |
| E87 | If your husband/partner wanted to use a male method of contraception such as condoms or withdrawal, would you agree? |  | yes_no_dkdr_nr_list | If not currently using male method. |
| E88 | If you wanted to use a female method of contraception such as pills, IUD, or implant, would your husband agree? |  | yes_no_dkdr_nr_list | If not currently using female method. |
| E89 | If you wanted to use emergency contraception, would your husband agree? |  | yes_no_dkdr_nr_list | If not currently using EC. |
| **Ask of those who are not currently using a method and have not used a method in the last 12 months.** | | | | |
| E90 | Do you know of a place where you can obtain a method of family planning? |  | yes_no_nr_list |  |
| **Ask of current non-users who do not want another child.** | | | | |
| E91 | You said that you do not want any / anymore children and that you are not using a method to avoid pregnancy. Can you tell me the reason that you are not using a method to prevent pregnancy? PROBE: Any other reasons? | Select all that apply. | nokids_list |  |
| E91 | Specify |  | text | If E91=other |
| **Ask of everyone.** | | | | |
| E92 | How old were you when you first had sexual intercourse? | The respondent said she was [age from FQ1] years old at her last birthday.  [She has had x live births.]  Enter the age in years.  Enter -77 if she never had sex.  Enter -88 if respondent does not know.  Enter -99 for no response. | numeric |  |
| E93 | (Confirm: You have entered that the respondent was X years old when she first had sexual intercourse. Is this what she said? ) |  | yes_no_nr_list |  |
| E94 | When was the last time you had sexual intercourse? | If less than 12 months ago, answer must be recorded in months, weeks, or days.  Enter 0 days for today. | numeric |  |
| E95 | When was the last time you had sexual intercourse? |  | lastsex_list |  |

**Section F: Relationships and Empowerment**

*Now I would like to talk about your thoughts about marital relationships.*

| **NO** | **QUESTION** | **RESPONSES** | | **SKIP** |
| --- | --- | --- | --- | --- |
|  | **Do you think it is proper for a wife to leave her husband if:** |  |  |  |
| F1 | He does not support her and the children financially? | Yes  No  No response | 1  0  -99 |  |
| F2 | He beats her? | Yes  No  No response | 1  0  -99 |  |
| F3 | He is sexually unfaithful? | Yes  No  No response | 1  0  -99 |  |
| F4 | She thinks he might be infected with HIV? | Yes  No  No response | 1  0  -99 |  |
| F5 | He does not allow her to use family planning? | Yes  No  No response | 1  0  -99 |  |
| F6 | He cannot provide her with children? | Yes  No  No response | 1  0  -99 |  |
| F7 | He does not sexually satisfy her? | Yes  No  No response | 1  0  -99 |  |
|  | **Do you think that a woman has a right to refuse unprotected sex with her husband if:** |  |  |  |
| F8 | She thinks he might have a sexually-transmitted infection? | Yes  No  No response | 1  0  -99 |  |
| F9 | She thinks she might have a sexually-transmitted infection? | Yes  No  No response | 1  0  -99 |  |
| F10 | She does not want to become pregnant? | Yes  No  No response | 1  0  -99 |  |
| F11 | For any reason at all? | Yes  No  No response | 1  0  -99 |  |
|  | **If a woman refuses to have sex with her husband, is it acceptable for the husband to:** |  |  |  |
| F12 | Have sex with another partner? | Yes  No  No response | 1  0  -99 |  |
| F13 | Have sex with her by force? | Yes  No  No response | 1  0  -99 |  |
| F14 | Beat her? | Yes  No  No response | 1  0  -99 |  |
| F15 | Withhold money from her? | Yes  No  No response | 1  0  -99 |  |
| F16 | Leave/divorce her? | Yes  No  No response | 1  0  -99 |  |
|  | **Is it acceptable for a wife to do the following?** |  |  |  |
|  | Earn income | Yes  No  No response | 1  0  -99 |  |
|  | Work outside the home | Yes  No  No response | 1  0  -99 |  |
|  | Have her own cell phone | Yes  No  No response | 1  0  -99 |  |
|  | Travel outside of the city without her husband | Yes  No  No response | 1  0  -99 |  |
|  | **Is it acceptable for a wife to do the following without informing her husband?** |  |  |  |
| F17 | Go to the market | Yes  No  No response | 1  0  -99 |  |
| F18 | Go to the health center | Yes  No  No response | 1  0  -99 |  |
| F19 | Leave the military camp | Yes  No  No response | 1  0  -99 |  |
|  | **Who usually makes decisions about the following: you, your husband/partner, you and your wife/partner jointly, or someone else?** |  |  |  |
| F20 | Making large household purchases | Self (wife)  Husband/partner  Joint decision  Someone else  No response | 1  2  3  4  -99 |  |
| F21 | Making household purchases for daily needs | Self (wife)  Husband/partner  Joint decision  Someone else  No response | 1  2  3  4  -99 |  |
| F22 | Getting medical treatment for yourself | Self (wife)  Husband/partner  Joint decision  Someone else  No response | 1  2  3  4  -99 |  |
| F23 | Buying clothes for yourself | Self (wife)  Husband/partner  Joint decision  Someone else  No response | 1  2  3  4  -99 |  |
| F24 | How your earnings will be used | Self (wife)  Husband/partner  Joint decision  Someone else  Not applicable, no earnings  No response | 1  2  3  4  5  -99 |  |
| F25 | How your partner’s earnings will be used | Self (wife)  Husband/partner  Joint decision  Someone else  Not applicable, no earnings  No response/Not applicable | 1  2  3  4  5  -99 |  |
| F26 | **Taking all things together, how satisfied are you with your marriage/relationship your husband/partner?** | Very satisfied  Satisfied  Dissatisfied  Very dissatisfied  No response | 1  2  3  4  -99 |  |
| F27 | **How much does he make you feel loved?** | A great deal  Somewhat  Not at all  No response | 1  2  3  -99 |  |
| F28 | **How often is he willing to listen when you need to talk about your worries or problems?** | Always/almost always  Often  Sometimes  Rarely  Never  No response | 1  2  3  4  5  -99 |  |
| F29 | **How often would you say the two of you have unpleasant disagreements or conflicts?** | Daily/almost daily  Two or three times a week  About once a week  2 or 3 times a month  About once a month  Less than once a month  Never  No response | 1  2  3  4  5  6  7  -99 |  |
| F30 | **How often do you feel bothered or upset by your relationship with your husband/partner?** | Always/almost always  Often  Sometimes  Rarely  Never  No response | 1  2  3  4  5  -99 |  |
| F31 | **Have you and your husband/partner ever discussed the number of children you would like to have?** | Yes  No  No response | 1  0  -99 |  |
| F32 | **How many more children does your husband/partner want to have?** | Number  Don’t know  No response | #  -88  -99 |  |

**Section G: Military Camp Population Characteristics**

| **NO** | **QUESTION** | **RESPONSES** | | **SKIP** |
| --- | --- | --- | --- | --- |
| G1 | In the past 12 months, how many times did you sleep away from your household for one night or more? | Number  No response | #  -99 |  |
| G2 | In what province were you born? | Kinshasa  Bas-Uele  Équateur  Haut-Katanga  Haut-Lomami  Haut-Uele  Ituri  Kasaï  Kasaï-Central  Kasaï-Oriental  Kongo Central  Kwango  Kwilu  Lomami  Lualaba  Mai-Ndombe  Maniema  Mongala  Nord-Ubangi  North Kivu  Sankuru  South Kivu  Sud-Ubangi  Tanganyika  Tshopo  Tshuapa  Other location (outside of DRC)  No response | 1  2  3  4  5  6  7  8  9  10  11  12  13  14  15  16  17  18  19  20  21  22  23  24  25  26  27  -99 |  |
| G3 | For how long have you lived in Kinshasa this time? | Months  Years  No response |  |  |
| G4 | For how long have you lived in this camp this time (not including deployments)? | Months  Years  No response |  |  |

Thank the respondent for her time.

The respondent is finished, but there are still 2 more questions for you to complete outside the home.

**Section H: Data collector questions**

| **NO** | **QUESTION** | **RESPONSES** | | **SKIP** |
| --- | --- | --- | --- | --- |
| H1 | **Location**  *Take a GPS point near the entrance to the household. Record location when the accuracy is smaller than 6m.*  *GPS coordinates can only be collected when outside.* |  |  |  |
| H2 | **How many times have you visited this household to interview this female respondent?** | 1^st^ time  2^nd^ time  3^rd^ time | 1  2  3 |  |
| H3 | **Questionnaire result** | Completed  Not at home  Postponed  Refused  Partly completed  Incapacitated | 1  2  3  4  5  6 |  |
