# Supplementary material for: The gap in contraceptive knowledge and use between the military and non-military populations of Kinshasa, DRC, 2016–2019
Source: PLoS One. 2021 Jul 27;16(7):e0254915. doi: 10.1371/journal.pone.0254915 (PMC8315532; doi:10.1371/journal.pone.0254915)
Supplement: S4 File — (DOCX) [file pone.0254915.s004.docx]

**S4 File. 2020 military survey questionnaire, French**

**Section A : Identification**

| **NO** | **QUESTION** | **REPONSES** | | **SAUT** |
| --- | --- | --- | --- | --- |
| A1 | **Etes-vous dans le bon ménage ?**  **Voici la photo de la maison vue de face, prise lors du Questionnaire ménage.**  **SI NON, RETOURNEZ DANS LE BON MENAGE POUR ADMINISTRER L’INTERVIEW.**  [ODK affichera la phone jointe au Questionnaire ménage lié] | Oui  Non | 1  0 |  |
| A2 | **Votre nom :** [Nom de l’enquêtrice du **Questionnaire femme**]  **Est-ce votre nom ?** | Oui  Non | 1  0 | Si “oui,” allez à A4. |
| A3 | **Tapez votre nom.**  *Veuillez enregistrer votre nom.* |  |  |  |
| A4 | **Date et heure actuelles.** [ODK les affichera à l’écran]  **Cette date et cette heure sont-elles exactes ?** | Oui  Non | 1  0 | If “oui,” skip to A6. |
| A5 | **Enregistrez la date et l’heure exactes.** |  |  |  |
| A6 | **Les informations suivantes ont été tirées du Questionnaire femme. Veuillez les relire pour vous assurer que vous êtes en train d’interroger la bonne personne.**  [ODK affichera la province, la ville, la commune et le quartier des ZD de Kinshasa, et la province, le district, l’aire de santé et le village pour celles du Kongo Central. De plus, les Zones de dénombrement, le Numéro de la structure et le Numéro du ménage saisis dans le Questionnaire ménage lié au Questionnaire femme s’afficheront également.]  **Les informations ci-dessus sont-elles correctes ?** | Oui  Non | 1  0 |  |
| A7 | **CONTRÔLE : Vous devriez être en train d’essayer d’interviewer [Nom de la répondante]. Est-ce exact ?**  *Si ce n’est pas la bonne personne, vous avez deux options :*  *(1) sortir et ignorer les changements de ce formulaire. Ouvrir le formulaire exact.*  *Ou*  *(2) trouver et interviewer la personne dont le nom s’affiche ci-dessus.* | Oui  Non | 1  0 |  |
| A8 | **La personne à enquêter est-elle présente et disponible aujourd’hui ?** | Oui  Non | 1  0 | Si “non,” allez à H1. |

**Section B : Consentement éclairé**

*Trouvez la femme entre 15 et 49 ans qui est associée à un Questionnaire de Suivi Femme. La femme doit être mariée ou vivre en union.*

*L’enquête doit avoir lieu en privé, sans que personne d’autre ne puisse entendre la conversation. Merci de lire le texte qui suit après avoir salué la répondante :*

*Bonjour. Je m’appelle ________________________________ et je travaille pour l’École de Santé Publique de l’Université de Kinshasa en collaboration avec le Ministère de la Santé. Nous menons actuellement une enquête à Kinshasa et au Kongo Central sur plusieurs thèmes liés à la santé reproductive. Nous aimerions beaucoup que vous fassiez partie de cette enquête. Les informations que nous collecterons aideront à informer le gouvernement afin de mieux planifier les services de santé. Le questionnaire prend généralement entre 15 et 20 minutes.*

*Toutes les informations que vous nous donnerez seront strictement confidentielles et ne seront partagées avec personne d’autre que les membres de notre équipe. Nous interviewerons également votre partenaire mais nous ne partagerons pas vos réponses avec lui. La participation à cette enquête est volontaire, et s’il y a une question à laquelle vous ne souhaitez pas répondre, faîtes le moi savoir et je passerai à la suivante ; ou vous pouvez également interrompre l’entretien à tout moment. Cependant, nous espérons que vous accepterez de participer à cette enquête car votre point de vue est important.*

*Est-ce que vous souhaitez maintenant me poser des questions concernant cette enquête ? Est-ce que vous êtes d’accord pour participer à cette enquête ?*

| **NO** | **QUESTION** | **REPONSES** | | **SAUT** |
| --- | --- | --- | --- | --- |
| B1 | *Veuillez remettre un exemplaire papier du Formulaire de consentement à la répondante et le lui expliquer. Puis, demandez :*  **Puis-je commencer l’interview à présent ?** | Oui  Non | 1  0 | Si “non,” allez à H1. |
| B2 | **Signature de la répondante**  *Demandez à la répondante de signer ou de cocher la case pour témoigner de son consentement à participer.* | Case à cocher:  |  |  |
| B3 | **Nom de l’enquêtrice :** [Nom de l’enquêtrice du Questionnaire ménage]  *Tapez votre nom en tant que témoin du processus de consentement.* |  |  |  |
| B4 | **Nom de la répondante :**  [ODK affichera le nom de la répondante du Tableau du ménage lié]  *Vous pouvez corriger l’orthographe ici si elle n’est pas exacte, mais vous devez interviewer la personne dont le nom s’affiche ci-dessous.* |  |  |  |

**Section C : Caractéristiques sociodémographiques, statut conjugal et caractéristiques du ménage de la répondante**

J’aimerais maintenant vous poser des questions sur vos origines et vos conditions socioéconomiques.

| **NO** | **QUESTION** | **REPONSES** | | **SAUT** |
| --- | --- | --- | --- | --- |
| C1 | **En quel mois et quelle année êtes-vous née ?**  **L’âge indiqué dans le tableau du ménage est [AGE].** | Mois :  Années : |  |  |
| C2 | **Quel âge avez-vous eu à votre dernier anniversaire ?**  *Doit coïncider avec C1.* |  |  | Si moins de 15, arrêtez l’interview et allez à H1. |
| C3 | **Quel est le plus haut niveau d’études que vous ayez atteint ?** | Jamais scolarisée  Primaire  Secondaire  Troisième cycle  Pas de réponse | 0  1  2  3  -99 |  |
| C4 | Are you in the military? | Yes No |  |  |
| C5 | What is your rank in this service? | Soldier 1st class  Soldier 2nd class  Corporal  Sargent  Sargent major  1st Sargent  1st Sargent Adjoint  Adjudant de 2ème classe  Adjudant  Adjudant in chef  Second Lieutenant  Lieutenant  Captain  Major  Lieutenant Colonel  Colonel  General Brigadier  Lieutenant General  General of Army Corps  General or Army | 1  2  3  4  5  6  7  8  9  10  11  12  13  14  15  16  17  18  19  20 |  |
| C6 | En général, diriez-vous que votre état de santé est :  *Lire les modalités de réponse.* | Excellent  Très bon  Bon  Moyen Faible  Pas de réponse | 1  2  3  4  5  -99 |  |
| C7 | **(Confirmez) Etes-vous actuellement mariée ou vivez-vous avez un homme comme si vous étiez mariés ?** | Non  Oui, mariée  Oui, vivent ensemble | 0  1  2 | Si “non,” arrêtez l’interview et allez à H1. |
| C8 | **Avez-vous été mariée ou avez-vous vécu avec un homme seulement une fois ou plus d’une fois ?** | Seulement une fois  Plus d’une fois  Pas de réponse | 1  2  -99 | Si “seulement une fois,” allez à C9. |
| C9 | **En quel mois et quelle année avez-vous commencé à vivre avec votre PREMIER mari/partenaire ?**  *Saisir Jan 2020 pour pas de réponse.* | Mois :  Année : |  |  |
| C10 | [Si ≤15 ans le jour du mariage, ODK affichera :]  **CONTRÔLE : Selon la réponse que vous avez saisie à la question C7, la répondante avait possiblement 15 ans ou moins lors de son premier mariage. Avez-vous enregistré la réponse C7 correctement ?** | Oui  Non | 1  0 |  |
| C11 | **J’aimerais maintenant vous demander quand vous avez commencé à vivre avec votre mari/partenaire ACTUEL ou LE PLUS RÉCENT. En quel mois et année était-ce ?**  *Saisir Jan 2020 pour pas de réponse.* | Mois :  Année : |  |  |
| C12 | [Si ≤15 ans le jour du mariage, ODK affichera :]  **CONTRÔLE : Selon la réponse que vous avez saisie à la question C9, la répondante avait possiblement 15 ans ou moins lors de son mariage le plus récent. Avez-vous enregistré la réponse C9 correctement ?** | Oui  Non | 1  0 |  |
| C13 | **Votre mari/partenaire a-t-il d’autres épouses, ou vit-il avec d’autres femmes comme s’ils étaient mariés ?** | Oui  Non  Ne sait pas  Pas de réponse | 1  0  -88  -99 |  |
| C14 | **Votre mari/partenaire vit-il avec vous en ce moment où ailleurs ?** | Vit avec la répondante  Vit ailleurs  Pas de réponse | 1  2  -99 |  |

**Section D : Préférences de reproduction et de fécondité**

J’aimerais à présent vous poser des questions sur tous les accouchements que vous avez eus pendant votre vie.

| **NO** | **QUESTION** | **REPONSES** | | **SAUT** |
| --- | --- | --- | --- | --- |
| D1 | **Combien de fois avez-vous été enceinte ?** |  |  |  |
| D2 | **Combien de fois avez-vous accouché ?**  *Zéro est une réponse possible.* | Nombre  Pas de réponse | #  -99 |  |
| D2 | **Ces naissances étaient-elles toutes des naissances vivantes ?**  *Si non, retournez en arrière et modifiez D2 pour n’enregistrer que les naissances vivantes.* | Oui  Non  Pas de réponse | 1  0  -99 |  |
| D3 | **Avez-vous donné naissance à un enfant qui est né vivant mais est décédé ensuite ?**  *SI NON, RELANCEZ : Un bébé qui pleure, montre des signes de vie mais ne survit pas.* | Oui  Non  Pas de réponse | 1  0  -99 | Si “non” ou “pas de réponse,” allez à D5. |
| D4 | **Combien sont décédés ?** | Nombre  Ne sait pas  Pas de réponse | #  -88  -99 |  |
| D5 | **Juste pour être sûre de ne pas me tromper : vous avez accouché__ fois au total dans votre vie, et de ces accouchements sont nés vivants __ fils et filles.**  **Est-ce exact ?** | Oui  Non  Pas de réponse | 1  0  -99 | Si “non,” revenez en arrière et corrigez D2-D4. |
| D6 | **Avez-vous adopté des enfants ou avez des enfants qui vivent avec vous au lieu de leurs parents biologiques ?** | Oui  Non | 1  0 | Si “non,” allez à D8. |
| D7 | **Combien de ces enfants avez-vous adoptés/vivent avec vous ?** |  |  |  |
| D8 | **Quand avez-vous accouché pour la PREMIÈRE FOIS ?**  *Veuillez enregistrer la date de la première naissance vivante. La date devrait être déterminée en remontant ou avançant le temps à partir d’événements mémorables au besoin. Saisir Jan 2020 pour pas de réponse.* | Mois  Année |  |  |
| D9 | **Quand avez-vous accouché LE PLUS RÉCEMMENT ?**  *Veuillez enregistrer la date de la naissance vivante LA PLUS RÉCENTE. La date devrait être déterminée en remontant ou avançant le temps à partir d’événements mémorables au besoin. Saisir Jan 2020 pour pas de réponse.* | Mois  Année |  | Si pas l’an dernier, et/ou D5=1, allez à D11. |
| D10 | **Quand avez-vous accouché avant votre accouchement le plus récent ?**  *Veuillez enregistrer la date de l’avant dernière naissance. La date devrait être déterminée en remontant le temps à partir d’événements mémorables au besoin. Saisir Jan 2020 pour pas de réponse.* | Mois  Année |  | Si D5 = 1, allez à D11 |
| D11 | **Votre dernier bébé/enfant est-il toujours en vie ?** | Oui  Non  Ne sait pas  Pas de réponse | 1  0  -88  -99 | Si “oui,” “ne sait pas,” ou “pas de réponse,” allez à D13. |
| D12 | **Quand votre dernier bébé/enfant est-il décédé ?**  *Veuillez enregistrer la date du décès de l’enfant.*  *La date devrait être déterminée en remontant le temps à partir d’événements mémorables au besoin. Saisir Jan 2020 pour pas de réponse.* | Mois  Année |  |  |
| D13 | **Quand vos dernières menstrues ont-elles commencé ?**  *Si vous sélectionnez des jours, semaines, mois ou années, vous devrez saisir un chiffre pour x à l’écran suivant.*  *Saisir 0 jours pour aujourd’hui, pas 0 semaines/mois/années.* | Il y a _ jours  Il y a _ semaines  Il y a _mois  Il y a _ans  Ménopause/hystérectomie  Avant le dernier accouchement  Jamais eu de menstrues  Pas de réponse | 5  6  7  -99 |  |
| D14 | **Etes-vous enceinte ?** | Oui  Non  Ne sait pas  Pas de réponse | 1  0  -88  -99 |  |
| D15 | **De combien de mois êtes-vous enceinte ?**  *Enregistrez le nombre de mois révolus.* | Nombre  Ne sait pas  Pas de réponse | 1-9  -88  -99 |  |
|  | *CONTRÔLE D14 : Enceinte ?* |  |  | Si pas enceinte, allez à D16.  Si enceinte, allez à D19. |
| D16 | **J’aimerais à présent vous poser une question sur votre dernier accouchement.**  **Quand vous êtes tombée enceinte, souhaitiez-vous être enceinte à ce moment-là, souhaitiez-vous atteindre un peu plus, ou est-ce que vous ne souhaitiez pas/plus avoir d’enfant du tout ?** | À ce moment là  Plus tard  Pas/plus du tout  Pas de réponse | 1  2  3  -99 |  |
| D17 | **J’ai quelques questions à vous poser sur votre avenir. Souhaitez-vous avoir un (autre) enfant, ou préférez-vous ne pas/plus en avoir ? a** | Avoir un (autre) enfant  Ne plus en avoir  Couple stérile  Indécise/Ne sait pas  Pas de réponse | 1  2  3  -88  -99 | Si “avoir un (autre) enfant,” allez à D18.  Pour toutes les autres réponses, allez à E1. |
| D18 | **Combien de temps aimeriez-vous attendre entre maintenant et la naissance de votre prochain enfant ?**  *Si vous sélectionnez mois ou années, vous devrez enregistrer un nombre pour x à l’écran suivant.*  *Sélectionnez “Années” si plus de 36 mois.* | Mois :  Années :  Bientôt/maintenant  Couple stérile  Autre  Ne sait pas  Pas de réponse | #  #  1  2  3  -88  -99 | Si “couple stérile,” allez à E15. Sinon, allez à D22. |
| D19 | **J’ai maintenant une question sur votre grossesse actuelle.**  **Quand vous êtes tombée enceinte, souhaitiez-vous être enceinte à ce moment-là, souhaitiez-vous atteindre un peu plus, ou est-ce que vous ne souhaitiez pas/plus avoir d’enfant du tout ?** | À ce moment là  Plus tard  Pas/plus du tout  Pas de réponse | 1  2  3  -99 |  |
| D20 | **Et maintenant, j’ai quelques questions sur votre avenir. Après l’enfant que vous attendez, souhaitez-vous avoir un autre enfant, ou préféreriez-vous ne plus en avoir ?** | Avoir un autre enfant  Ne plus en avoir  Couple stérile  Indécise/Ne sait pas  Pas de réponse | 1  2  3  -88  -99 | Si PAS “avoir un autre enfant,” allez à E1. |
| D21 | **Après la naissance de l’enfant que vous attendez, combien de temps souhaitez-vous attendre avant d’avoir un autre enfant ?**  *Si vous sélectionnez mois ou années, vous devrez enregistrer un nombre pour x à l’écran suivant.*  *Sélectionnez “Années” si plus de 36 mois.* | Mois :  Années :  Bientôt/maintenant  Couple stérile  Autre  Ne sait pas  Pas de réponse | #  #  1  2  3  -88  -99 | Si “couple stérile,” allez à E1. |
| D22 | **Au total, combien d’enfants de plus souhaitez-vous avoir ?** | Nombre  Indécise/Ne sait pas  Pas de réponse | # -88  -99 |  |

**Section E. Contraception**

**J’aimerais à présent vous parler de la planification familiale, soit les différentes méthodes qu’un couple peut utiliser pour retarder ou éviter une grossesse.**

Une image s’affichera à l’écran pour certaines méthodes. Si la répondante déclare qu’elle n’a pas entendu parler de la méthode en question ou si elle hésite à répondre, lisez la relance à voix haute et montrez-lui l’image, le cas échéant.

| **NO** | **QUESTION** | **REPONSES** | | **SAUT** |
| --- | --- | --- | --- | --- |
| E1 | **Avez-vous déjà entendu parler de la stérilisation féminine ?**  RELANCE : Les femmes peuvent se faire opérer pour ne plus avoir d’enfant.  [PAS D’IMAGE] | Oui  Non  Pas de réponse | 1  0  -99 | Si non, allez à E5. |
| E2 | **Vous êtes-vous déjà fait opérer pour ne plus avoir d’enfant (stérilisation féminine/ligature des trompes) ?** | Oui  Non  Pas de réponse | 1  0  -99 | Si non, allez à E5. |
| E3 | **Quand avez-vous eu recours à la stérilisation féminine ?** | Jour :  Mois :  Année : |  | Saisir 00 si date inconnue |
| E4 | **Pourquoi avez-vous eu recours à la stérilisation féminine ?** | N’aimait pas la méthode antérieure  Seule méthode disponible  Financièrement abordable  Facile à utiliser  Recommandation du prestataire de santé  Efficace  Effets secondaires limités/inexistants  Préférence du partenaire  Discret/partenaire ne le sait pas  Recommandée par une amie/famille  Durée d’utilisation  Autre :______________  Pas de réponse | 1  2  3  4  5  6  7  8  9  10  11  12  -99 |  |
| E5 | **Avez-vous entendu parler de la stérilisation masculine ?**  RELANCE : Les hommes peuvent se faire opérer pour ne plus avoir d’enfant.  [PAS D’IMAGE] | Oui  Non  Pas de réponse | 1  0  -99 | Si non, allez à E9. |
| E6 | **Votre mari/partenaire s’est-il fait opérer pour ne plus avoir d’enfant (stérilisation masculine/vasectomie) ?** | Oui  Non  Pas de réponse | 1  0  -99 | Si non, allez à E11. |
| E7 | **Quand a-t-il eu recours à la stérilisation masculine ?** | Jour :  Mois :  Année : |  | Saisir 00 si date inconnue |
| E8 | **Pourquoi a-t-il eu recours à la stérilisation masculine ?** | N’aimait pas la méthode antérieure  Seule méthode disponible  Financièrement abordable  Facile à utiliser  Recommandation du prestataire de santé  Efficace  Effets secondaires limités/inexistants  Préférence du partenaire  ~~Discret/partenaire ne le sait pas~~  Recommandée par un ami/famille  Durée d’utilisation  Autre :______________  Pas de réponse | 1  2  3  4  5  6  7  8  9  10  11  12  -99 |  |
| E9 | **A-t-il eu recours à un renversement de stérilisation masculine depuis ?** | Oui  Non  Pas de réponse | 1  0  -99 | Si non, allez à E11. |
| E10 | **Quand a-t-il eu recours au renversement de stérilisation masculine?** | Jour :  Mois :  Année : |  | Saisir 00 si date inconnue |
| E11 | **Avez-vous déjà entendu parler de l’implant contraceptif ?**  RELANCE : Les femmes peuvent se faire insérer un ou plusieurs petits bâtonnets dans le haut du bras par un médecin ou un infirmier, ce qui permet de ne pas tomber enceinte pendant un an ou plus.  [UNE IMAGE S’AFFICHERA] | Oui  Non  Pas de réponse | 1  0  -99 | Si non, allez à E19. |
| E12 | **Avez-vous déjà utilisé l’implant contraceptif ?** | Oui  Non  Pas de réponse | 1  0  -99 | Si non, allez à E19. |
| E13 | **Quand avez-vous utilisé l’implant contraceptif pour la première fois ?** | Jour :  Mois :  Année : |  | Saisir 00 si date inconnue |
| E14 | **Pour quelle raison principale avez-vous commencé à utiliser l’implant contraceptif ?** | N’aimait pas la méthode antérieure  Seule méthode disponible  Financièrement abordable  Facile à utiliser  Recommandation du prestataire de santé  Efficace  Effets secondaires limités/inexistants  Préférence du partenaire  Discret/partenaire ne le sait pas  Recommandée par une amie/famille  Durée d’utilisation  Autre :______________  Pas de réponse | 1  2  3  4  5  6  7  8  9  10  11  12  -99 |  |
| E15 | **Quand vous êtes-vous fait insérer un implant contraceptif pour la dernière fois ?** | Jour :  Mois :  Année : |  | Saisir 00 si date inconnue |
| E16 | **Avez-vous actuellement un implant contraceptif ?** | Oui  Non  Pas de réponse | 1  0  -99 | If oui, skip to E19. |
| E17 | **Quand vous êtes-vous fait retirer votre implant contraceptif ?** | Jour :  Mois :  Année : |  | Saisir 00 si date inconnue |
| E18 | **Pour quelle raison principale avez-vous arrêté d’utiliser l’implant contraceptif ?** | Rapports sexuels peu fréquents/mari absent  Est tombée enceinte en l’utilisant  Voulait tomber enceinte  Mari/partenaire opposé  Voulait une méthode plus efficace  Méthode non disponible  Préoccupations de santé  Peur des effets secondaires  Manque d’accès/ trop loin  Trop cher  Peu pratique à utiliser  Fataliste  Difficulté à concevoir/  ménopausée  Interfère avec les processus du corps  Autre : __________  Ne sait pas  Pas de réponse | 1  2  3  4  5  6  7  8  9  10  11  12  13  14  15  -99 |  |
| E19 | **Avez-vous déjà entendu parler du DIU ?**  RELANCE : Les femmes peuvent se faire insérer un dispositif en forme de T dans leur cavité utérine par un médecin ou un infirmier.  [UNE IMAGE S’AFFICHERA] | Oui  Non  Pas de réponse | 1  0  -99 | Si non, allez à E27. |
| E20 | **Avez-vous déjà utilisé le DIU ?** | Oui  Non  Pas de réponse | 1  0  -99 | Si non, allez à E27. |
| E21 | **Quand avez-vous utilisé le DIU pour la première fois ?** | Jour :  Mois :  Année : |  | Saisir 00 si date inconnue. |
| E22 | **Pour quelle raison principale avez-vous commencé à utiliser le DIU ?** | N’aimait pas la méthode antérieure  Seule méthode disponible  Financièrement abordable  Facile à utiliser  Recommandation du prestataire de santé  Efficace  Effets secondaires limités/inexistants  Préférence du partenaire  Discret/partenaire ne le sait pas  Recommandée par une amie/famille  Durée d’utilisation  Autre :______________  Pas de réponse | 1  2  3  4  5  6  7  8  9  10  11  12  -99 |  |
| E23 | **Quand vous êtes-vous fait insérer un DIU pour la dernière fois ?** | Jour :  Mois :  Année : |  | Saisir 00 si date inconnue |
| E24 | **Avez-vous un DIU actuellement ?** | Oui  Non  Pas de réponse | 1  0  -99 | Si oui, allez à E27. |
| E25 | **Quand vous êtes-vous fait retirer votre DIU ?** | Jour :  Mois :  Année : |  | Saisir 00 si date inconnue |
| E26 | **Pour quelle raison principale avez-vous arrêté d’utiliser le DIU ?** | Rapports sexuels peu fréquents/mari absent  Est tombée enceinte en l’utilisant  Voulait tomber enceinte  Mari/partenaire opposé  Voulait une méthode plus efficace  Méthode non disponible  Préoccupations de santé  Peur des effets secondaires  Manque d’accès/ trop loin  Trop cher  Peu pratique à utiliser  Fataliste  Difficulté à concevoir/  ménopausée  Interfère avec les processus du corps  Autre : __________  Ne sait pas  Pas de réponse | 1  2  3  4  5  6  7  8  9  10  11  12  13  14  15  -99 |  |
| E27 | **Avez-vous déjà entendu parler des contraceptifs injectables ?**  RELANCE : Les femmes peuvent se faire administrer une injection par un prestataire de santé pour ne pas tomber enceinte pendant un mois ou plus.  [IMAGES DE SAYANA PRESS ET DEPO PROVERA S’AFFICHERONT À L’ÉCRAN] | Oui  Non  Pas de réponse | 1  0  -99 | If no, skip to E38. |
| E28 | **Avez-vous déjà utilisé un contraceptif injectable ?** | Oui  Non  Pas de réponse | 1  0  -99 | If no, skip to E38. |
| E29 | **Quand avez-vous utilisé pour la première fois un contraceptif injectable ?** | Jour :  Mois :  Année : |  | Enter 00 if unknown |
| E30 | **Pour quelle raison principale avez-vous commencé à utiliser le contraceptif injectable ?** | N’aimait pas la méthode antérieure  Seule méthode disponible  Financièrement abordable  Facile à utiliser  Recommandation du prestataire de santé  Efficace  Effets secondaires limités/inexistants  Préférence du partenaire  Discret/partenaire ne le sait pas  Recommandée par une amie/famille  Durée d’utilisation  Autre :______________  Pas de réponse | 1  2  3  4  5  6  7  8  9  10  11  12  -99 |  |
| E31 | **L’injection a-t-elle été administrée via une seringue ou une petite aiguille la première fois ?**  *Montrez l’image à la répondante.*  [DES IMAGES DES DEUX SYSTEMES D’INJECTION S’AFFICHERONT A L’ECRAN] | Seringue  Petite aiguille (Sayana Press)  Les deux  Ne sait pas  Pas de réponse | 1  2  3  -88  -99 | Si pas “petite aiguille,” allez à E33. |
| E32 | **Où avez-vous obtenu votre injection de Sayana Press pour la première fois ?** | Centre de santé/hôpital  Distributeur à base communautaire (DBC)  Pharmacie  Auto-injection  Autre lieu  Pas de réponse | 1  2  3  4  5  -99 |  |
| E33 | **Quand avez-vous obtenu votre injection pour la dernière fois ?** | Jour :  Mois :  Année : |  | Saisir 00 si date inconnue |
| E234 | **L’injection a-t-elle été administrée via une seringue ou une petite aiguille la dernière fois ?**  *Montrez l’image à la répondante.*  [DES IMAGES DES DEUX SYSTEMES D’INJECTION S’AFFICHERONT A L’ECRAN] | Seringue  Petite aiguille (Sayana Press)  Les deux  Ne sait pas  Pas de réponse | 1  2  3  -88  -99 | Si pas “petite aiguille,” allez à E36. |
| E35 | **Où avez-vous obtenu votre injection de Sayana Press pour la dernière fois ?** | Centre de santé/hôpital  Distributeur à base communautaire (DBC)  Pharmacie  Auto-injection  Autre lieu  Pas de réponse | 1  2  3  4  5  -99 |  |
| E36 | **Utilisez-vous actuellement l’injectable pour ne pas tomber enceinte ?** | Oui  Non  Pas de réponse | 1  0  -99 | Si oui ou pas de réponse, allez à E38. |
| E37 | **Pour quelle raison principale avez-vous arrêté d’utiliser des injectables ?** | Rapports sexuels peu fréquents/mari absent  Est tombée enceinte en l’utilisant  Voulait tomber enceinte  Mari/partenaire opposé  Voulait une méthode plus efficace  Méthode non disponible  Préoccupations de santé  Peur des effets secondaires  Manque d’accès/ trop loin  Trop cher  Peu pratique à utiliser  Fataliste  Difficulté à concevoir/  ménopausée  Interfère avec les processus du corps  Autre : __________  Ne sait pas  Pas de réponse | 1  2  3  4  5  6  7  8  9  10  11  12  13  14  15  -99 |  |
| E38 | **Avez-vous déjà entendu parler de la pilule (contraceptive) ?**  RELANCE : Les femmes peuvent prendre une pilule tous les jours pour ne pas tomber enceintes.  [UNE IMAGE S’AFFICHERA] | Oui  Non  Pas de réponse | 1  0  -99 | Si non, allez à E45. |
| E39 | **Avez-vous déjà entendu parler de la pilule ?** | Oui  Non  Pas de réponse | 1  0  -99 | Si non, allez à E45. |
| E40 | **Quand avez-vous pris la pilule pour la première fois ?** | Jour :  Mois :  Année : |  | Saisir 00 si date inconnue |
| E41 | **Pour quelle raison principale avez-vous commencé à utiliser la pilule ?** | N’aimait pas la méthode antérieure  Seule méthode disponible  Financièrement abordable  Facile à utiliser  Recommandation du prestataire de santé  Efficace  Effets secondaires limités/inexistants  Préférence du partenaire  Discret/partenaire ne le sait pas  Recommandée par une amie/famille  Durée d’utilisation  Autre :______________  Pas de réponse | 1  2  3  4  5  6  7  8  9  10  11  12  -99 |  |
| E42 | **Quand avez-vous pris la pilule pour la dernière fois ?** | Jour :  Mois :  Année : |  | Saisir 00 si date inconnue |
| E43 | **Prenez-vous actuellement la pilule pour éviter de tomber enceinte ?** | Oui  Non  Pas de réponse | 1  0  -99 | If oui or no response, skip to E45. |
| E44 | **Pour quelle raison principale avez-vous arrêté de prendre la pilule ?** | Rapports sexuels peu fréquents/mari absent  Est tombée enceinte en l’utilisant  Voulait tomber enceinte  Mari/partenaire opposé  Voulait une méthode plus efficace  Méthode non disponible  Préoccupations de santé  Peur des effets secondaires  Manque d’accès/ trop loin  Trop cher  Peu pratique à utiliser  Fataliste  Difficulté à concevoir/  ménopausée  Interfère avec les processus du corps  Autre : __________  Ne sait pas  Pas de réponse | 1  2  3  4  5  6  7  8  9  10  11  12  13  14  15  -99 |  |
| E45 | **Avez-vous entendu parler de la contraception d’urgence ?**  RELANCE : Par mesure d’urgence après un rapport sexuel non protégé, les femmes peuvent prendre une pilule spéciale dans les cinq jours suivant le rapport sexuel non protégé pour éviter de tomber enceintes.  [PAS D’IMAGE] | Oui  Non  Pas de réponse | 1  0  -99 | Si non, allez à E52. |
| E46 | **Avez-vous déjà utilisé la contraception d’urgence ?** | Oui  Non  Pas de réponse | 1  0  -99 | Si non, allez à E52. |
| E47 | **Quand avez-vous utilisé la contraception d’urgence pour la première fois ?** | Jour :  Mois :  Année : |  | Saisir 00 si date inconnue |
| E48 | **Pour quelle raison principale avez-vous commencé à utiliser la contraception d’urgence ?** | N’aimait pas la méthode antérieure  Seule méthode disponible  Financièrement abordable  Facile à utiliser  Recommandation du prestataire de santé  Efficace  Effets secondaires limités/inexistants  Préférence du partenaire  Discret/partenaire ne le sait pas  Recommandée par une amie/famille  Durée d’utilisation  Autre :______________  Pas de réponse | 1  2  3  4  5  6  7  8  9  10  11  12  -99 |  |
| E49 | **Quand avez-vous pris la contraception d’urgence pour la dernière fois ?** | Jour :  Mois :  Année : |  | Saisir 00 si date inconnue |
| E50 | **La prochaine fois que vous aurez un rapport sexuel, pensez-vous que vous utiliserez la contraception d’urgence pour éviter de tomber enceinte ?** | Oui  Non  Pas de réponse | 1  0  -99 | Si oui, allez à E52. |
| E51 | **Pourquoi n’utiliserez-vous pas la contraception d’urgence la prochaine fois ?** | Rapports sexuels peu fréquents/mari absent  Est tombée enceinte en l’utilisant  Voulait tomber enceinte  Mari/partenaire opposé  Voulait une méthode plus efficace  Méthode non disponible  Préoccupations de santé  Peur des effets secondaires  Manque d’accès/ trop loin  Trop cher  Peu pratique à utiliser  Fataliste  Difficulté à concevoir/  ménopausée  Interfère avec les processus du corps  Autre : __________  Ne sait pas  Pas de réponse | 1  2  3  4  5  6  7  8  9  10  11  12  13  14  15  -99 |  |
| E52 | **Avez-vous entendu parler du préservatif ?**  RELANCE : Les hommes peuvent placer un matériau étanche et élastique sur leur pénis avant un rapport sexuel.  [UNE IMAGE S’AFFICHERA] | Oui  Non  Pas de réponse | 1  0  -99 | Si non, allez à E59. |
| E53 | **Avez-vous déjà utilisé le préservatif ?** | Oui  Non  Pas de réponse | 1  0  -99 | Si non, allez à E59. |
| E54 | **Quand avez-vous utilisé le préservatif pour la première fois ?** | Jour :  Mois :  Année : |  | Saisir 00 si date inconnue |
| E55 | **Pour quelle raison principale avez-vous commencé à utiliser le préservatif ?** | N’aimait pas la méthode antérieure  Seule méthode disponible  Financièrement abordable  Facile à utiliser  Recommandation du prestataire de santé  Efficace  Effets secondaires limités/inexistants  Préférence du partenaire  Discret/partenaire ne le sait pas  Recommandée par une amie/famille  Durée d’utilisation  Autre :______________  Pas de réponse | 1  2  3  4  5  6  7  8  9  10  11  12  -99 |  |
| E56 | **Quand avez-vous utilisé le préservatif pour la dernière fois ?** | Jour :  Mois :  Année : |  | Saisir 00 si date inconnue |
| E57 | **La prochaine fois que vous aurez un rapport sexuel, pensez-vous que vous utiliserez le préservatif pour éviter de tomber enceinte ?** | Oui  Non  Pas de réponse | 1  0  -99 | Si oui, allez à E59. |
| E58 | **Pourquoi n’utiliserez-vous pas le préservatif la prochaine fois ?** | Rapports sexuels peu fréquents/mari absent  Est tombée enceinte en l’utilisant  Voulait tomber enceinte  Mari/partenaire opposé  Voulait une méthode plus efficace  Méthode non disponible  Préoccupations de santé  Peur des effets secondaires  Manque d’accès/ trop loin  Trop cher  Peu pratique à utiliser  Fataliste  Difficulté à concevoir/  ménopausée  Interfère avec les processus du corps  Autre : __________  Ne sait pas  Pas de réponse | 1  2  3  4  5  6  7  8  9  10  11  12  13  14  15  -99 |  |
| E59 | **Avez-vous entendu parler du préservatif féminin ?**  RELANCE : Les femmes peuvent placer un matériau étanche et élastique dans leur vagin avant un rapport sexuel.  [UNE IMAGE S’AFFICHERA] | Oui  Non  Pas de réponse | 1  0  -99 | Si non, allez à E66. |
| E60 | **Avez-vous déjà utilisé le préservatif féminin ?** | Oui  Non  Pas de réponse | 1  0  -99 | Si non, allez à E66. |
| E61 | **Quand avez-vous utilisé le préservatif féminin pour la première fois ?** | Jour :  Mois :  Année : |  | Saisir 00 si date inconnue |
| E62 | **Pour quelle raison principale avez-vous commencé à utiliser le préservatif féminin ?** | N’aimait pas la méthode antérieure  Seule méthode disponible  Financièrement abordable  Facile à utiliser  Recommandation du prestataire de santé  Efficace  Effets secondaires limités/inexistants  Préférence du partenaire  Discret/partenaire ne le sait pas  Recommandée par une amie/famille  Durée d’utilisation  Autre :______________  Pas de réponse | 1  2  3  4  5  6  7  8  9  10  11  12  -99 |  |
| E63 | **Quand avez-vous utilisé le préservatif féminin pour la dernière fois ?** | Jour :  Mois :  Année : |  | Saisir 00 si date inconnue |
| E64 | **La prochaine fois que vous aurez un rapport sexuel, pensez-vous que vous utiliserez un préservatif féminin pour éviter de tomber enceinte ?** | Oui  Non  Pas de réponse | 1  0  -99 | Si oui, allez à E66. |
| E65 | **Pourquoi n’utiliserez-vous pas le préservatif féminin ?** | Rapports sexuels peu fréquents/mari absent  Est tombée enceinte en l’utilisant  Voulait tomber enceinte  Mari/partenaire opposé  Voulait une méthode plus efficace  Méthode non disponible  Préoccupations de santé  Peur des effets secondaires  Manque d’accès/ trop loin  Trop cher  Peu pratique à utiliser  Fataliste  Difficulté à concevoir/  ménopausée  Interfère avec les processus du corps  Autre : __________  Ne sait pas  Pas de réponse | 1  2  3  4  5  6  7  8  9  10  11  12  13  14  15  -99 |  |
| E66 | **Avez-vous entendu parler du diaphragme ?**  RELANCE : Les femmes peuvent placer un disque fin et flexible dans leur vagin avant un rapport sexuel.  [UNE IMAGE S’AFFICHERA] | Oui  Non  Pas de réponse | 1  0  -99 | Si non, allez à E73. |
| E67 | **Avez-vous déjà utilisé le diaphragme ?** | Oui  Non  Pas de réponse | 1  0  -99 | Si non, allez à E73. |
| E68 | **Quand avez-vous commencé à utiliser le diaphragme pour la première fois ?** | Jour :  Mois :  Année : |  | Saisir 00 si date inconnue |
| E69 | **Pour quelle raison principale avez-vous commencé à utiliser le diaphragme ?** | N’aimait pas la méthode antérieure  Seule méthode disponible  Financièrement abordable  Facile à utiliser  Recommandation du prestataire de santé  Efficace  Effets secondaires limités/inexistants  Préférence du partenaire  Discret/partenaire ne le sait pas  Recommandée par une amie/famille  Durée d’utilisation  Autre :______________  Pas de réponse | 1  2  3  4  5  6  7  8  9  10  11  12  -99 |  |
| E70 | **Quand avez-vous utilisé le diaphragme pour la dernière fois ?** | Jour :  Mois :  Année : |  | Saisir 00 si date inconnue |
| E71 | **La prochaine fois que vous aurez un rapport sexuel, pensez-vous que vous utiliserez le diaphragme pour éviter de tomber enceinte ?** | Oui  Non  Pas de réponse | 1  0  -99 | Si oui, allez à E73. |
| E72 | **Pourquoi n’utiliserez-vous pas le diaphragme la prochaine fois ?** | Rapports sexuels peu fréquents/mari absent  Est tombée enceinte en l’utilisant  Voulait tomber enceinte  Mari/partenaire opposé  Voulait une méthode plus efficace  Méthode non disponible  Préoccupations de santé  Peur des effets secondaires  Manque d’accès/ trop loin  Trop cher  Peu pratique à utiliser  Fataliste  Difficulté à concevoir/  ménopausée  Interfère avec les processus du corps  Autre : __________  Ne sait pas  Pas de réponse | 1  2  3  4  5  6  7  8  9  10  11  12  13  14  15  -99 |  |
| E73 | **Avez-vous entendu parler de la mousse ou gelée comme méthode de contraception ?**  RELANCE : Les femmes peuvent appliquer un suppositoire, de la gelée ou de la crème dans leur vagin avant un rapport sexuel pour éviter de tomber enceintes.  [UNE IMAGE S’AFFICHERA] | Oui  Non  Pas de réponse | 1  0  -99 | Si non, allez à E80. |
| E74 | **Avez-vous déjà utilisé de la mousse ou de la gelée ?** | Oui  Non  Pas de réponse | 1  0  -99 | Si non, allez à E80. |
| E75 | **Quand avez-vous utilisé de la mousse ou de la gelée pour la première fois ?** | Jour :  Mois :  Année : |  | Saisir 00 si date inconnue |
| E76 | **Pour quelle raison principale avez-vous commencé à utiliser de la mousse ou de la gelée ?** | N’aimait pas la méthode antérieure  Seule méthode disponible  Financièrement abordable  Facile à utiliser  Recommandation du prestataire de santé  Efficace  Effets secondaires limités/inexistants  Préférence du partenaire  Discret/partenaire ne le sait pas  Recommandée par une amie/famille  Durée d’utilisation  Autre :______________  Pas de réponse | 1  2  3  4  5  6  7  8  9  10  11  12  -99 |  |
| E77 | **Quand avez-vous utilisé de la mousse ou de la gelée pour la dernière fois ?** | Jour :  Mois :  Année : |  | Saisir 00 si date inconnue |
| E78 | **La prochaine fois que vous aurez un rapport sexuel, pensez-vous que vous utiliserez de la mousse ou de la gelée pour éviter de tomber enceinte ?** | Oui  Non  Pas de réponse | 1  0  -99 | Si oui, allez à E80. |
| E79 | **Pourquoi n’utiliserez-vous pas de la mousse ou de la gelée la prochaine fois ?** | Rapports sexuels peu fréquents/mari absent  Est tombée enceinte en l’utilisant  Voulait tomber enceinte  Mari/partenaire opposé  Voulait une méthode plus efficace  Méthode non disponible  Préoccupations de santé  Peur des effets secondaires  Manque d’accès/ trop loin  Trop cher  Peu pratique à utiliser  Fataliste  Difficulté à concevoir/  ménopausée  Interfère avec les processus du corps  Autre : __________  Ne sait pas  Pas de réponse | 1  2  3  4  5  6  7  8  9  10  11  12  13  14  15  -99 |  |
| E80 | **Avez-vous entendu parler de la Méthode des Jours Fixes ou du Collier du cycle ?**  RELANCE : Les femmes peuvent utiliser un collier de perles de couleur pour connaître les jours où elles peuvent tomber enceintes. Les jours où elles peuvent tomber enceintes, elles ou leurs partenaires utilisent un préservatif ou s’abstiennent d’avoir des rapports sexuels.  [UNE IMAGE S’AFFICHERA] | Oui  Non  Pas de réponse | 1  0  -99 | Si non, allez à E87. |
| E81 | **Avez-vous déjà utilisé la Méthode des Jours Fixes ou le Collier du cycle ?** | Oui  Non  Pas de réponse | 1  0  -99 | Si non, allez à E87. |
| E82 | **Quand avez-vous utilisé la Méthode des Jours Fixes ou le Collier du cycle pour la première fois ?** | Jour :  Mois :  Année : |  | Saisir 00 si date inconnue |
| E83 | **Pour quelle méthode principale avez-vous commencé à utiliser la Méthode des Jours Fixes ou le Collier du cycle ?** | N’aimait pas la méthode antérieure  Seule méthode disponible  Financièrement abordable  Facile à utiliser  Recommandation du prestataire de santé  Efficace  Effets secondaires limités/inexistants  Préférence du partenaire  Discret/partenaire ne le sait pas  Recommandée par une amie/famille  Durée d’utilisation  Autre :______________  Pas de réponse | 1  2  3  4  5  6  7  8  9  10  11  12  -99 |  |
| E84 | **Quand avez-vous utilisé la Méthode des Jours Fixes ou le Collier du cycle pour la dernière fois ?** | Jour :  Mois :  Année : |  | Saisir 00 si date inconnue |
| E85 | **Utilisez-vous actuellement la Méthode des Jours Fixes ou le Collier du cycle pour éviter de tomber enceinte ?** | Oui  Non  Pas de réponse | 1  0  -99 | Si oui ou pas de réponse, allez à E87. |
| E86 | **Pourquoi avez-vous arrêté d’utiliser la Méthode des Jours Fixes/Collier du cycle ?** | Rapports sexuels peu fréquents/mari absent  Est tombée enceinte en l’utilisant  Voulait tomber enceinte  Mari/partenaire opposé  Voulait une méthode plus efficace  Méthode non disponible  Préoccupations de santé  Peur des effets secondaires  Manque d’accès/ trop loin  Trop cher  Peu pratique à utiliser  Fataliste  Difficulté à concevoir/  ménopausée  Interfère avec les processus du corps  Autre : __________  Ne sait pas  Pas de réponse | 1  2  3  4  5  6  7  8  9  10  11  12  13  14  15  -99 |  |
| E87 | **Avez-vous entendu parler de la Méthode de l’aménorrhée de lactation, ou MAMA ?**  [PAS DE DESCRIPTION ; PAS D’IMAGE] | Oui  Non  Pas de réponse | 1  0  -99 | Si non, allez à E94. |
| E88 | **Avez-vous déjà utilisé la méthode MAMA ?** | Oui  Non  Pas de réponse | 1  0  -99 | Si non, allez à E94. |
| E89 | **Quand avez-vous utilisé la méthode MAMA pour la première fois ?** | Jour :  Mois :  Année : |  |  |
| E90 | **Pour quelle raison principale avez-vous commencé à utiliser la méthode MAMA ?** | N’aimait pas la méthode antérieure  Seule méthode disponible  Financièrement abordable  Facile à utiliser  Recommandation du prestataire de santé  Efficace  Effets secondaires limités/inexistants  Préférence du partenaire  Discret/partenaire ne le sait pas  Recommandée par une amie/famille  Durée d’utilisation  Autre :______________  Pas de réponse | 1  2  3  4  5  6  7  8  9  10  11  12  -99 |  |
| E91 | **Quand avez-vous utilisé la méthode MAMA pour la dernière fois ?** | Jour :  Mois :  Année : |  |  |
| E92 | **Utilisez-vous actuellement la méthode MAMA pour éviter de tomber enceinte ?** | Oui  Non  Pas de réponse | 1  0  -99 | Si oui ou pas de réponse, allez à E94. |
| E93 | **Pourquoi avez-vous arrêté d’utiliser la méthode MAMA ?** | Rapports sexuels peu fréquents/mari absent  Est tombée enceinte en l’utilisant  Voulait tomber enceinte  Mari/partenaire opposé  Voulait une méthode plus efficace  Méthode non disponible  Préoccupations de santé  Peur des effets secondaires  Manque d’accès/ trop loin  Trop cher  Peu pratique à utiliser  Fataliste  Difficulté à concevoir/  ménopausée  Interfère avec les processus du corps  Autre : __________  Ne sait pas  Pas de réponse | 1  2  3  4  5  6  7  8  9  10  11  12  13  14  15  -99 |  |
| E94 | **Avez-vous entendu parler de la méthode du rythme ?**  RELANCE : Les femmes peuvent éviter de tomber enceintes en s’abstenant d’avoir des rapports sexuels les jours du mois où elles pensent qu’elles peuvent tomber enceintes.  [PAS D’IMAGE] | Oui  Non  Pas de réponse | 1  0  -99 | Si non, allez à E101. |
| E95 | **Avez-vous déjà utilisé la méthode du rythme ?** | Oui  Non  Pas de réponse | 1  0  -99 | Si non, allez à E101. |
| E96 | **Quand avez-vous utilisé la méthode du rythme pour la première fois ?** | Jour :  Mois :  Année : |  | Saisir 00 si date inconnue |
| E97 | **Pour quelle raison principale avez-vous commencé à utiliser la méthode du rythme ?** | N’aimait pas la méthode antérieure  Seule méthode disponible  Financièrement abordable  Facile à utiliser  Recommandation du prestataire de santé  Efficace  Effets secondaires limités/inexistants  Préférence du partenaire  Discret/partenaire ne le sait pas  Recommandée par une amie/famille  Durée d’utilisation  Autre :______________  Pas de réponse | 1  2  3  4  5  6  7  8  9  10  11  12  -99 |  |
| E98 | **Quand avez-vous utilisé la méthode du rythme pour la dernière fois ?** | Jour :  Mois :  Année : |  | Saisir 00 si date inconnue |
| E99 | **Utilisez-vous actuellement la méthode du rythme pour éviter de tomber enceinte ?** | Oui  Non  Pas de réponse | 1  0  -99 | Si oui ou pas de réponse, allez à E101. |
| E100 | **Pour quelle raison principale avez-vous arrêté d’utiliser la méthode du rythme ?** | Rapports sexuels peu fréquents/mari absent  Est tombée enceinte en l’utilisant  Voulait tomber enceinte  Mari/partenaire opposé  Voulait une méthode plus efficace  Méthode non disponible  Préoccupations de santé  Peur des effets secondaires  Manque d’accès/ trop loin  Trop cher  Peu pratique à utiliser  Fataliste  Difficulté à concevoir/  ménopausée  Interfère avec les processus du corps  Autre : __________  Ne sait pas  Pas de réponse | 1  2  3  4  5  6  7  8  9  10  11  12  13  14  15  -99 |  |
| E101 | **Avez-vous entendu parler de la méthode du retrait ou du coït interrompu ?**  RELANCE : Les hommes peuvent faire attention et se retirer avant l’éjaculation.  [PAS D’IMAGE] | Oui  Non  Pas de réponse | 1  0  -99 | Si non, allez à E108. |
| E102 | **Avez-vous déjà utilisé la méthode du retrait ?** | Oui  Non  Pas de réponse | 1  0  -99 | Si non, allez à E108. |
| E103 | **Quand avez-vous utilisé la méthode du retrait pour la première fois ?** | Jour :  Mois :  Année : |  | Saisir 00 si date inconnue |
| E104 | **Pour quelle raison principale avez-vous commencé à utiliser la méthode du retrait ?** | N’aimait pas la méthode antérieure  Seule méthode disponible  Financièrement abordable  Facile à utiliser  Recommandation du prestataire de santé  Efficace  Effets secondaires limités/inexistants  Préférence du partenaire  Discret/partenaire ne le sait pas  Recommandée par une amie/famille  Durée d’utilisation  Autre :______________  Pas de réponse | 1  2  3  4  5  6  7  8  9  10  11  12  -99 |  |
| E105 | **Quand avez-vous utilisé la méthode du retrait pour la dernière fois ?** | Jour :  Mois :  Année : |  | Saisir 00 si date inconnue |
| E106 | **La prochaine fois que vous aurez un rapport sexuel, pensez-vous que vous utiliserez la méthode du retrait pour éviter de tomber enceinte ?** | Oui  Non  Pas de réponse | 1  0  -99 | Si oui ou pas de réponse, allez à E108. |
| E107 | **Pourquoi n’utiliserez-vous pas la méthode du retrait la prochaine fois ?** | Rapports sexuels peu fréquents/mari absent  Est tombée enceinte en l’utilisant  Voulait tomber enceinte  Mari/partenaire opposé  Voulait une méthode plus efficace  Méthode non disponible  Préoccupations de santé  Peur des effets secondaires  Manque d’accès/ trop loin  Trop cher  Peu pratique à utiliser  Fataliste  Difficulté à concevoir/  ménopausée  Interfère avec les processus du corps  Autre : __________  Ne sait pas  Pas de réponse | 1  2  3  4  5  6  7  8  9  10  11  12  13  14  15  -99 |  |
| E108 | **Avez-vous entendu parler d’autres manières ou méthodes que les femmes ou les hommes peuvent utiliser pour éviter une grossesse ?** | Oui  Non  Pas de réponse | 1  0  -99 | Si “non,” ou “pas de réponse,” passez au contrôle avant E110. |
| E109 | **De quelles autres méthodes avez-vous entendu parler ?** | [Question ouverte] |  |  |
|  | ***CONTROLE*** *: a déjà utilisé une méthode. Si oui, passez à E110. Si non, allez à E111.* |  |  |  |
| E110 | **Rappelez-vous de la première fois que vous avez utilisé une méthode pour retarder ou éviter une grossesse : combien d’enfants en vie aviez-vous à ce moment-là ?**  *NB : la répondante a déclaré avoir donné naissance [nombre de naissances vivantes] fois à la question D2.* | Nombre  Pas de réponse | #  -99 |  |
| E111 | **Pensez-vous que vous utiliserez ou continuerez d’utiliser une méthode de contraception pour retarder ou éviter une grossesse à un moment donné plus tard ?** | Oui  Non  Pas de réponse | 1  0  -99 | Si non ou pas de réponse, allez à E113. |
| E112 | **Quels types de contraception pensez-vous que vous utiliserez ?**  **RELANCE : Rien d’autre ?**  *Sélectionnez toutes les méthodes mentionnées. Assurez-vous de faire dérouler la liste jusqu’en bas pour voir toutes les modalités de réponse.* | Stérilisation féminine  Stérilisation masculine  Implant  DIU  Injectable  Pilule  Contraception d’urgence  Préservatif masculin  Préservatif féminin  Diaphragme  Mousse/Gelée  Jours Fixes/Collier du cycle  MAMA  Rythme  Retrait  Autre traditionnelle  Pas de réponse | 1  2  3  4  5  6  7  8  9  10  11  12  13  14  15  16  -99 |  |
|  | ***CONTROLE :*** *Déjà utilisé la contraception ?* |  |  | Si non, allez à E129. |
| E113 | **Avant de commencer à utiliser [METHODE ACTUELLE/ LA PLUS RECENTE], avez-vous parlé à votre mari/partenaire de la possibilité d’utiliser une méthode de contraception ?** | Oui  Non  Ne sait pas  Pas de réponse | 1  0  -88  -99 |  |
| E114 | **Does your partner know that you are using a family planning method?** | Yes  No  Don’t know  No response | 1  0  -88  -99 |  |
| E115 | **Does your partner approve of you using a family planning method?** | Yes  No  Don’t know  No response | 1  0  -88  -99 |  |
| E116 | **Vous avez commencé à utiliser [MÉTHODE ACTUELLE/LA PLUS RECENTE] en [DATE DE FQ27]. Où avez-vous cette méthode à ce moment-là ?**  *Faites dérouler la liste jusqu’en bas pour voir toutes les modalités de réponse.* | Hôpital/Clinique  Centre de santé  Poste sanitaire  Pharmacie  ASC  Boutique/Ligablo  Autre  Ne sait pas  Pas de réponse | 1  2  3  4  5  6  7  -88  -99 |  |
| E117 | **Quand vous avez obtenu [METHODE ACTUELLE/ LA PLUS RECENTE], le prestataire vous a-t-il parlé des effets secondaires ou problèmes que vous pourriez rencontrer avec cette méthode pour retarder ou éviter une grossesse ?** | Oui  Non  Ne sait pas  Pas de réponse | 1  0  -88  -99 | Si non, allez à E19. |
| E118 | **Vous a-t-on dit ce qu’il fallait faire en cas d’effets secondaires ou de problèmes ?** | Oui  Non  Ne sait pas  Pas de réponse | 1  0  -88  -99 |  |
| E119 | **À ce moment-là, le prestataire vous a-t-il parlé d’autres méthodes de planification familiale à part [METHODE ACTUELLE/LA PLUS RECENTE] que vous pourriez utiliser ?** | Oui  Non  Ne sait pas  Pas de réponse | 1  0  -88  -99 |  |
| E120 | **Pendant cette consultation, avez-vous obtenu la méthode que vous vouliez pour retarder ou éviter une grossesse ?** | Oui  Non  Ne sait pas  Pas de réponse | 1  0  -88  -99 | Si oui, allez à E122. |
| E121 | **Pourquoi n’avez-vous pas obtenu la méthode que vous vouliez ?** | La méthode était en rupture de stock ce jour-là  La méthode n’était pas disponible du tout  Le prestataire n’était pas formé pour fournir la méthode  Le prestataire a recommandé une autre méthode  Pas éligible à la méthode  Décidé de ne pas choisir de méthode du tout  Trop cher  Autre  Ne sait pas  Pas de réponse | 1  2  3  4  5  6  7  8  -88  -99 |  |
| E122 | **Pendant cette consultation, qui a pris la décision finale de la méthode que vous avez obtenue ?** | Vous seule  Prestataire  Partenaire  Vous et le prestataire  Vous et votre partenaire  Autre  Ne sait pas  Pas de réponse | 1  2  3  4  5  6  -88  -99 |  |
| E123 | **Retourneriez-vous voir ce prestataire ?**  *Prestataire : [Type de prestataire de E34]* | Oui  Non  Pas de réponse | 1  0  -99 |  |
| E124 | **Recommanderiez-vous ce prestataire/ structure sanitaire à une amie ou un membre de votre famille ?** | Oui  Non  Pas de réponse | 1  0  -99 |  |
| E125 | **Dans les 12 derniers mois, votre mari/partenaire et vous-même avez-vous payé des frais pour des services de planification familiale (y compris la méthode actuelle/la plus récente) ?** | Oui  Non  Pas de réponse | 1  0  -99 | Si non, allez à E127. |
| E126 | **Au total, combien vous et votre mari/partenaire avez-vous payé pour des services de planification familiale dans les 12 derniers mois ?**  *Saisir tous les prix en francs congolais.* | Francs  Ne sait pas  Pas de réponse | #  -88  -99 |  |
| E127 | **Où avez-vous obtenu [METHODE ACTUELLE] la dernière fois ?** | Hôpital/Clinique  Centre de santé  Poste sanitaire  Pharmacie  ASC  Boutique/Ligablo  Autre  Ne sait pas  Pas de réponse | 1  2  3  4  5  6  7  -88  -99 |  |
| E128 | **Avez-vous obtenu votre méthode actuelle dans le camp militaire ou dehors ?** | Dans le camp  En dehors du camp  Pas de réponse | 1  2  -99 |  |
| E129 | **Où préféreriez-vous obtenir votre méthode ?** | Hôpital/Clinique  Centre de santé  Poste sanitaire  Pharmacie  ASC  Boutique/Ligablo  Autre  Ne sait pas  Pas de réponse | 1  2  3  4  5  6  7  -88  -99 |  |
| E130 | **Pourquoi préférez-vous obtenir cette méthode de ce type de prestataire ?**  *Sélectionnez toutes les réponses mentionnées.* | Moins cher avec ce prestataire plutôt qu’un autre  Le prestataire est près de chez moi  Le prestataire est près de mon travail  Je connais le prestataire  Je connais quelqu’un qui travaille ici /avec ce prestataire  Je pense que ce prestataire a de meilleures connaissances/expertise que les autres prestataires  Je pense que ce prestataire est plus gentil/respectueux que les autres prestataires  Une amie ou un membre de ma famille me l’a recommandé  J’ai vu une pancarte ou une publicité disant que ce prestataire offrait la planification familiale  Ce prestataire est confidentiel / anonyme  Ce prestataire est accessible  Je sais que je peux obtenir la méthode de PF de mon choix avec ce prestataire  Les méthodes de PF sont gratuites avec ce prestataire  Autre  Ne sait pas  Pas de réponse | 1  2  3  4  5  6  7  8  9  10  11  12  13  14  -88  -99 |  |
| E131 | **Dans les 12 derniers mois, un agent de santé communautaire vous a-t-il rendu visite pour vous parler de la planification familiale ?** | Oui  Non  Ne sait pas  Pas de réponse | 1  0  -88  -99 |  |
| E132 | **Dans les 12 derniers mois, avez-vous participé à une discussion de groupe au niveau communautaire sur la planification familiale ?** | Oui  Non  Ne sait pas  Pas de réponse | 1  0  -88  -99 |  |
| E133 | **Dans les 12 derniers mois, vous êtes-vous rendue dans un centre de santé pour obtenir des soins pour vous-même ou vos enfants ?**  *Pour quelque type de service de santé que ce soit.* | Oui  Non  Ne sait pas  Pas de réponse | 1  0  -88  -99 |  |
| E134 | **Est-ce qu’un membre du personnel de la structure de santé vous a parlé des méthodes de planification familiale ?** | Oui  Non  Ne sait pas  Pas de réponse | 1  0  -88  -99 |  |
| E135 | **Dans les derniers mois, avez-vous entendu parler de la planification familiale à la radio ?** | Oui  Non  Ne sait pas  Pas de réponse | 1  0  -88  -99 |  |
| E136 | **Dans les derniers mois, avez-vous vu quelque chose sur la planification familiale à la télévision ?** | Oui  Non  Ne sait pas  Pas de réponse | 1  0  -88  -99 |  |
| E137 | **Dans les derniers mois, avez-vous lu quelque chose sur la planification familiale dans un journal ou un magazine ?** | Oui  Non  Ne sait pas  Pas de réponse | 1  0  -88  -99 |  |
| E138 | **Avez-vous déjà vu cette image ?**    ***Montrez le logo à la répondante.***  **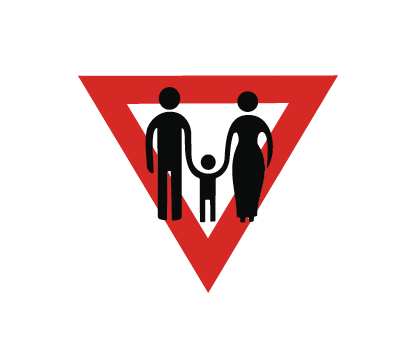** | Oui  Non  Ne sait pas  Pas de réponse | 1  0  -88  -99 | Si non ou pas de réponse, allez à E141. |
| E139 | **Où avez-vous vu cette image ?**  **RELANCE : Ailleurs ?**  *Sélectionnez toutes les réponses mentionnées.* | Centre de santé  Rue  Pharmacie  Panneau d’affichage  Télévision  Dépliant  Veste/Gilet d’un ASC  Autre  Ne sait pas  Pas de réponse | 1  2  3  4  5  6  7  8  -88  -99 |  |
| E140 | **Que veut dire cette image ?**  *Sélectionnez toutes les réponses mentionnées.* | Planification familiale  Espacement des naissances  Santé reproductive  Unité familiale  Autre  Ne sait pas  Pas de réponse | 1  2  3  4  5  -88  -99 |  |
| E141 | **Avez-vous vu ce panneau ou un similaire (montrant une famille militaire) ?**  **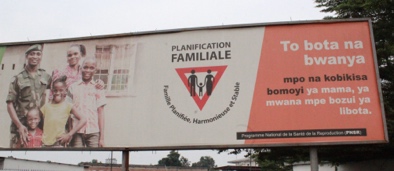**  *Montrez une photo du panneau à la répondante.* | Oui  Non  Ne sait pas  Pas de réponse | 1  0  -88  -99 |  |
| E142 | **Que veut dire cette image ?**  *Sélectionnez toutes les réponses mentionnées.* | Planification familiale  Espacement des naissances  Santé reproductive  Unité familiale  Autre  Ne sait pas  Pas de réponse | 1  2  3  4  5  -88  -99 |  |
| E143 | **Have you ever seen an activity in your community called “Lelo FP?”** | Yes  No  Don’t know  No response | 1  0  -88  -99 | Si non, allez à E145. |
| E144 | **Have you ever received a family planning method from a Lelo FP event?** | Oui  Non  Pas de réponse | 1  2  -99 |  |
| E145 | **Si votre mari/partenaire voulait utiliser la contraception masculine réversible (stérilisation masculine, préservatif, etc.) seriez-vous d’accord ?** | Oui  Non  Dépend de la méthode  Pas de réponse | 1  2  3  -99 |  |
| E146 | **Si vous vouliez utiliser la contraception réversible (implant, UID, etc.), serait-il d’accord ?** | Oui  Non  Dépend de la méthode  Pas de réponse | 1  2  3  -99 |  |
| E147 | **Connaissez-vous un endroit où vous pouvez obtenir une méthode de planification familiale ?** | Oui  Non  Pas de réponse | 1  0  -99 |  |
| E148 | **Contrôle D1- & D12 :** Désir d’avoir d’autres enfants  **Contrôle D11 & D13** : 2 ans ou plus avant le prochain enfant ?  **Contrôle E1-E178 :** Utilise actuellement une méthode de contraception ? |  |  | Posez aux non utilisatrices (actuelles ou n’ayant jamais utilisé) qui ne veulent pas (d’autre) enfant ou pas avant 2 ans. Sinon, allez à E145. |
| E149 | **Vous avez dit que vous ne voulez pas/plus avoir d’enfant et que vous n’utilisez aucune méthode pour éviter de tomber enceinte.**  **Pouvez-vous me dire les raisons pour lesquelles vous n’utilisez pas de méthode pour empêcher une grossesse ?**  **RELANCE : D’autres raisons ?**  *ENREGISTREZ TOUTES LES REPONSES MENTIONNEES.*  *Ne peut sélectionner “Ne sait pas” ou “Pas de réponse” avec d’autres options.*  *Ne peut sélectionner “Non mariée” si la réponse à C5 est “Oui, mariée”.*  *Faites défiler la liste jusqu’en bas pour voir toutes les modalités de réponse.* | Pas mariée  Rapports sexuels peu fréquents  Ménopausée /Hystérectomie  Difficulté à concevoir/ stérile  Pas eu ses menstrues depuis la dernière naissance  Allaitement  Séparés/ éloignés pour plusieurs jours  S’en remet à Dieu/ fataliste  Répondante opposée  Mari/ partenaire opposé  Autres opposés  Prohibition religieuse  Ne connait aucune méthode  Ne connait aucune source  Peur des effets secondaires  Préoccupations de santé  Manque d’accès/ trop loin  Trop cher  Méthode préférée non disponible  Aucune méthode disponible  Peu pratique à utiliser  Interfère avec les processus du corps  Prend trop de temps sur les obligations régulières/ trop occupée  Intention d’en utiliser une mais pas encore eu l’occasion d’aller au centre de santé  Mère opposée  Autre  Ne sait pas  Pas de réponse | 1  2  3  4  5  6  7  8  9  10  11  12  13  14  15  16  17  18  19  20  21  22  23  24  25  26  -88  -99 |  |
|  | **VERIFIEZ QU’IL N’Y AIT PERSONNE D’AUTRE PRESENT. AVANT DE CONTINUER, ASSUREZ VOUS QUE PERSONNE NE PUISSE VOUS ENTENDRE.**  ***Préparez verbalement la répondante aux questions sur l’activité sexuelle.*** |  |  |  |
| E150 | **Quel âge aviez-vous quand vous avez eu des rapports sexuels pour la première fois ?**  ***La répondante a déclaré avoir eu [âge de FQ1] ans à son dernier anniversaire.***  ***[Elle a eu x naissances vivantes.]***  *Saisir l’âge en années.*  *Saisir -77 si n’a jamais eu de rapport sexuel.*  *Saisir -88 si ne sait pas.*  *Saisir -99 pour pas de réponse.* | Age  N’a jamais eu de rapport sexuel  Ne sait pas  Pas de réponse | #  -77  -88  -99 | Si “n’a jamais eu de rapport sexuel,” allez à F1. |
| E151 | [Si l’âge au premier rapport sexuel est <10 ans :]  **Vous avez enregistré que la répondante avait X ans lors de son premier rapport sexuel. Est-ce bien ce qu’elle a dit ?**  *Retournez en arrière et corrigez FQ48 si ce n’est pas exact.* | Oui  Non | 1  0 |  |
| E152 | **Quand avez-vous eu un rapport sexuel pour la dernière fois ?**  *S’il y a moins de 12 mois, la réponse doit être enregistrée en mois, semaines ou jours.*  *Saisir 0 jours pour aujourd’hui.* | Il y a _ jours  Il y a _ semaines  Il y a _ mois  Il y a _ ans |  |  |

**Section F : Relations et autonomie**

*Maintenant, j’aimerais vous poser quelques questions sur ce que vous pensez des relations conjugales.*

| **NO** | **QUESTION** | **REPONSES** | | **SAUT** |
| --- | --- | --- | --- | --- |
|  | **Pensez-vous qu’il soit approprié pour une femme de quitter son mari si :** |  |  |  |
| F1 | Il ne soutient pas sa femme et ses enfants financièrement ? | Oui  Non  Pas de réponse | 1  0  -99 |  |
| F2 | Il la bat ? | Oui  Non  Pas de réponse | 1  0  -99 |  |
| F3 | Il lui est sexuellement infidèle ? | Oui  Non  Pas de réponse | 1  0  -99 |  |
| F4 | Elle pense qu’il pourrait être atteint du VIH ? | Oui  Non  Pas de réponse | 1  0  -99 |  |
| F5 | Il ne lui permet pas d’utiliser la planification familiale ? | Oui  Non  Pas de réponse | 1  0  -99 |  |
| F6 | Il ne peut pas lui donner d’enfant ? | Oui  Non  Pas de réponse | 1  0  -99 |  |
| F7 | Il ne la satisfait pas sexuellement ? | Oui  Non  Pas de réponse | 1  0  -99 |  |
|  | **Pensez-vous qu’une femme a le droit de refuser d’avoir un rapport sexuel non protégé avec son mari si :** |  |  |  |
| F8 | Elle pense qu’il pourrait avoir une infection sexuellement transmissible ? | Oui  Non  Pas de réponse | 1  0  -99 |  |
| F9 | Elle pense qu’elle pourrait avoir une infection sexuellement transmissible ? | Oui  Non  Pas de réponse | 1  0  -99 |  |
| F10 | Elle ne veut pas tomber enceinte ? | Oui  Non  Pas de réponse | 1  0  -99 |  |
| F11 | Pour toute autre raison ? | Oui  Non  Pas de réponse | 1  0  -99 |  |
|  | **Si une femme refuse d’avoir un rapport sexuel avec son mari, est-il acceptable que le mari :** |  |  |  |
| F12 | Ait des rapports sexuels avec une autre partenaire ? | Oui  Non  Pas de réponse | 1  0  -99 |  |
| F13 | Ait des rapports sexuels avec elle par la force ? | Oui  Non  Pas de réponse | 1  0  -99 |  |
| F14 | La batte ? | Oui  Non  Pas de réponse | 1  0  -99 |  |
| F15 | Lui confisque de l’argent ? | Oui  Non  Pas de réponse | 1  0  -99 |  |
| F16 | La quitte/se divorce d’elle ? | Oui  Non  Pas de réponse | 1  0  -99 |  |
|  | **Est-ce acceptable pour une femme mariée de faire les choses suivantes ?** |  |  |  |
|  | Gagner des revenus | Oui  Non  Pas de réponse | 1  0  -99 |  |
|  | Travailler en dehors de chez elle | Oui  Non  Pas de réponse | 1  0  -99 |  |
|  | Avoir son propre téléphone portable | Oui  Non  Pas de réponse | 1  0  -99 |  |
|  | Voyager en dehors de la ville sans son mari | Oui  Non  Pas de réponse | 1  0  -99 |  |
|  | **Est-il acceptable pour une femme mariée de faire les choses suivantes sans en informer son mari ?** |  |  |  |
| F17 | Aller au marché | Oui  Non  Pas de réponse | 1  0  -99 |  |
| F18 | Aller au centre de santé | Oui  Non  Pas de réponse | 1  0  -99 |  |
| F19 | Quitter le camp militaire | Oui  Non  Pas de réponse | 1  0  -99 |  |
|  | **Qui prend généralement les décisions sur les sujets suivants : vous, votre mari/partenaire, vous et votre mari/partenaire conjointement, ou quelqu’un d’autre ?** |  |  |  |
| F20 | Gros achats pour le ménage | Moi-même (femme)  Mari/partenaire  Décision commune  Quelqu’un d’autre  Pas de réponse | 1  2  3  4  -99 |  |
| F21 | Achats pour les besoins quotidiens du ménage | Moi-même (femme)  Mari/partenaire  Décision commune  Quelqu’un d’autre  Pas de réponse | 1  2  3  4  -99 |  |
| F22 | Obtenir un traitement médical pour vous-même | Moi-même (femme)  Mari/partenaire  Décision commune  Quelqu’un d’autre  Pas de réponse | 1  2  3  4  -99 |  |
| F23 | Acheter des vêtements pour vous-même | Moi-même (femme)  Mari/partenaire  Décision commune  Quelqu’un d’autre  Pas de réponse | 1  2  3  4  -99 |  |
| F24 | Comment vos revenus seront dépensés | Moi-même (femme)  Mari/partenaire  Décision commune  Quelqu’un d’autre  Non applicable, pas de revenus  Pas de réponse | 1  2  3  4  5  -99 |  |
| F25 | Comment les revenus de votre mari seront dépensés | Moi-même (femme)  Mari/partenaire  Décision commune  Quelqu’un d’autre  Non applicable, pas de revenus  Pas de réponse | 1  2  3  4  5  -99 |  |
| F26 | **Globalement, quel est votre degré de satisfaction de votre mariage/relation avec votre mari/partenaire ?** | Très satisfaite  Satisfaite  Insatisfaite  Très insatisfaite  Pas de réponse | 1  2  3  4  -99 |  |
| F27 | **À quel point vous fait-il vous sentir aimée ?** | Beaucoup  Un peu  Pas du tout  Pas de réponse | 1  2  3  -99 |  |
| F28 | **À quelle fréquence est-il disposé à vous écouter quand vous avez besoin de parler de vos préoccupations ou vos problèmes ?** | Toujours/presque toujours  Souvent  Parfois  Rarement  Jamais  Pas de réponse | 1  2  3  4  5  -99 |  |
| F29 | **À quelle fréquence diriez-vous que vous rencontrez tous les deux des désaccords désagréables ou êtes en conflit ?** | Tous les jours/Presque tous les jours  Deux ou trois fois par semaine  Environ une fois par semaine  2 ou 3 fois par mois  Environ une fois par mois  Moins d’une fois par mois  Jamais  Pas de réponse | 1  2  3  4  5  6  7  -99 |  |
| F30 | **À quelle fréquence vous sentez-vous dérangée ou contrariée par votre relation avec votre mari/partenaire ?** | Toujours/presque toujours  Souvent  Parfois  Rarement  Jamais  Pas de réponse | 1  2  3  4  5  -99 |  |
| F31 | **Votre mari/partenaire et vous-même avez-vous déjà parlé du nombre d’enfants que vous souhaitez avoir ?** | Oui  Non  Pas de réponse | 1  0  -99 |  |
| F32 | **Combien d’autres enfants votre mari/partenaire veut-il ?** | Nombre  Ne sait pas  Pas de réponse | #  -88  -99 |  |
|  | *CONTRÔLE C7d : A des enfants ?* |  |  | Si “pas d’enfant,” allez à G1. |
| F33 | **J’aimerais maintenant vous poser une question sur votre dernier enfant.**  **Quand vous êtes tombée enceinte, souhaitiez-vous tomber enceinte à ce moment-là, souhaitiez-vous attendre un peu, ou ne souhaitiez-vous pas/plus avoir d’enfant du tout ?** | Souhaitait tomber enceinte  Souhaitait attendre  Ne voulait pas d’enfant  Ne sait pas  Pas de réponse | 1  2  3  -88  -99 |  |

**Section G : Caractéristiques démographiques du camp militaire**

| **NO** | **QUESTION** | **REPONSES** | | **SAUT** |
| --- | --- | --- | --- | --- |
| G1 | Dans les 12 derniers mois, combien de fois avez-vous dormi en dehors de votre ménage pour une nuit ou plus ? | Nombre  Pas de réponse | #  -99 |  |
| G2 | Dans quelle province êtes-vous née ? | Kinshasa  Bas-Uele  Équateur  Haut-Katanga  Haut-Lomami  Haut-Uele  Ituri  Kasaï  Kasaï-Central  Kasaï-Oriental  Kongo Central  Kwango  Kwilu  Lomami  Lualaba  Mai-Ndombe  Maniema  Mongala  Nord-Ubangi  Nord Kivu  Sankuru  Sud Kivu  Sud-Ubangi  Tanganyika  Tshopo  Tshuapa  Autre lieu de naissance (en dehors de la RDC)  Pas de réponse | 1  2  3  4  5  6  7  8  9  10  11  12  13  14  15  16  17  18  19  20  21  22  23  24  25  26  27  -99 |  |
| G3 | Depuis combien de temps vivez-vous à Kinshasa cette fois-ci ? | Mois  Années  Pas de réponse |  |  |
| G4 | Depuis combien de temps vivez-vous dans ce camp cette fois-ci (sans compter les déploiements) ? | Mois  Années  Pas de réponse |  |  |

Remerciez la répondante du temps qu’elle a bien voulu vous accorder.

Elle a terminé, mais vous devez répondre encore à 2 questions en dehors de la maison.

**Section H : Questions pour l’enquêtrice**

| **NO** | **QUESTION** | **REPONSES** | | **SAUT** |
| --- | --- | --- | --- | --- |
| H1 | **Lieu**  *Prenez une coordonnée GPS près de l’entrée du ménage. Enregistrez la géolocalisation lorsque l’exactitude est inférieure à 6m.*  *Les coordonnées GPS ne peuvent être collectées que de dehors.* |  |  |  |
| H2 | **Combien de fois vous êtes-vous rendue dans ce ménage pour interviewer cette répondante femme ?** | 1^ère^ fois  2^ème^ fois  3^ème^ fois | 1  2  3 |  |
| H3 | **Résultat du questionnaire** | Terminé  Pas à son domicile  Reporté  Refusé  Terminé en partie  Incapacité | 1  2  3  4  5  6 |  |
