# Supplementary material for: The gap in contraceptive knowledge and use between the military and non-military populations of Kinshasa, DRC, 2016–2019
Source: PLoS One. 2021 Jul 27;16(7):e0254915. doi: 10.1371/journal.pone.0254915 (PMC8315532; doi:10.1371/journal.pone.0254915)
Supplement: S1 Table — (DOCX) [file pone.0254915.s005.docx]

**S1 Table. Adjusted Odd Ratios (OR) for factors associated with modern contraceptive use in Kinshasa, 2016–2019**

| Explanatory variables | Uses modern contraception | | | |
| --- | --- | --- | --- | --- |
|  | OR | Standard Error | 95% CI | p-value |
| Population |  |  |  |  |
| Non-Military | 1.00 |  |  |  |
| Military | 0.37 | 0.37 | 0.16 – 0.85 | **0.018** |
| Year of Survey |  |  |  |  |
| 2016 | 1.00 |  |  |  |
| 2019 | 2.15 | 0.39 | 1.52 – 3.06 | **<0.001** |
| Interaction term (Population*Year) | 1.14 | 0.70 | 0.34 – 3.83 | 0.838 |
| Age | 0.92 | 0.01 | 0.90 – 0.95 | **<0.001** |
| Number of live births | 1.18 | 0.05 | 1.08 – 1.29 | **<0.001** |
| Desire for another child(ren) |  |  |  |  |
| No | 1.00 |  |  |  |
| Yes | 1.88 | 0.42 | 1.21 – 2.93 | **0.005** |
| Level of education |  |  |  |  |
| None/Primary | 1.00 |  |  |  |
| Middle/secondary | 0.90 | 0.16 | 0.63 – 1.28 | 0.552 |
| Tertiary | 1.43 | 0.37 | 0.86 – 2.36 | 0.165 |
| Husband has other wives |  |  |  |  |
| No | 1.00 |  |  |  |
| Yes | 1.03 | 0.26 | 0.64 – 1.68 | 0.897 |
| Staff member at health facility spoke to you about FP methods in the past 12 months |  |  |  |  |
| No | 1.00 |  |  |  |
| Yes | 1.40 | 0.32 | 0.90 – 2.18 | 0.131 |
| Saw FP messages via the media (score) | 1.24 | 0.12 | 1.01 – 1.51 | **0.035** |
| Visited by Health worker in the past 12 months |  |  |  |  |
| No | 1.00 |  |  |  |
| Yes | 1.71 | 0.36 | 1.14 – 2.57 | **0.010** |
| Constant | 1.03 | 0.41 | 0.48 – 2.23 | 0.937 |
| Log likelihood | **-1313.20** |  |  |  |
| Number of level 1 units | **2517** |  |  |  |
| Number of level 2 units | **66** |  |  |  |
