# Supplementary material for: The gap in contraceptive knowledge and use between the military and non-military populations of Kinshasa, DRC, 2016–2019
Source: PLoS One. 2021 Jul 27;16(7):e0254915. doi: 10.1371/journal.pone.0254915 (PMC8315532; doi:10.1371/journal.pone.0254915)
Supplement: Attachment — Submitted filename: Response to reviewers.docx [file pone.0254915.s008.docx]

**Authors’ replies to the editors/reviewers’ comments: ONE-D-21-04034**

**(previous title) Closing the Gap: Contraceptive dynamics in military and non-military populations of Kinshasa, DRC, 2016-2020**

**(new title) The Gap in Contraceptive Knowledge and Use between the Military and Non-military populations of Kinshasa, DRC, 2016-2019**

*Note: in this document, all comments from the editors/reviewers are shown in bold (non-italics). All responses from the authors are shown in non-bold italics. In addition, we would like to draw attention to the change in the data of the second survey in our revised manuscript. Based on the actual dates of data collection, the surveys in both the military and non-military populations are much closer to a three- than a four-year interval. (Because the data collection for the second survey in both cases extended into 2020, we had opted for simplicity to describe it as a comparison of 2016 and 2019. We now specify that there was a three-year interval and (again for simplicity) have labeled the second surveys as occurring in 2019, although the exact dates are given in the methods section.*

*On May 26, 2021, the editor/reviewer sent us a series of new comments to address. Below are those points, as well as our replies.*

**The data link to the military survey,** [**http://doi.org/10.5061/dryad.k0p2ngf85**](http://doi.org/10.5061/dryad.k0p2ngf85)**, does not work.**

*Reply: we have experienced difficulty in posting the data set to Dryad. Instead, we are submitting it directly to the journal as supplementary file S6. If this is not satisfactory, please let us know.*

**The main concern on the editor side is that the revision did not take into proper consideration the main limitation raised in the previous version: That the military populations of 2016 and 2020 are different populations as it can be grasped from table 1. This is understandable, since military population might be deployed to different settlements, maybe some of the barracks have a specific “meaning” attached (eg: for new recruits, …). The fact is that the populations are different and that more can be done in the analysis regarding this.**

*Reply: we concur and have edited the text consistent with the points below.*

**1. It does not invalidate the analysis, but there should be no suggestions that the military population in the two periods is the same. The title changed, which is in the right direction since we do not know if there is a closing gap or the “new” military population comes from a different background? More examples that remain:**

**l. 43: “Similarly, modern contraceptive use increased significantly in both 44 populations, but the military women continued to trail their non-military counterparts on MCPR 45 (24.2% versus 29.7%.)”**

*Reply: we have reworded the results section of the abstract to read:*

*The socio-demographic profile of women in the military camps differed between 2016 and 2019, which may reflect the more mobile nature of this population. In both populations, knowledge of modern contraceptive methods increased significantly. Similarly, use of a modern contraceptive method also increased significantly in both, though by 2019 women in the military camps were less likely to use modern contraception (24.9%) than their non-military counterparts (29.7%). Multivariate analysis showed no significant difference in the amount of increase in MCPR for the two populations. Among contraceptive users in both populations, the implant was the leading method. Potential effects of FP programming were evident in the military population: exposure to FP messaging increased (in comparison to a decrease among the non-military population). Moreover, women who had lived in the camps for 4+ years had a higher MCPR than those living in the camps for less than four years.*

**l. 47: “by 2019 the women in the military population had caught up to where the non-military population was as of 2016.”**

*Reply: see above; line removed.*

**You can use more neutral language talking about higher levels of contraceptive use or increase. You should avoid language that suggest change in behaviour “the women in …”: They are (or might be) different women. These two examples come from the abstract. Please revise thoroughly the text.**

*Reply: we have attempted to simply describe the military population in 2019 without implying a change in behavior. For example, when referring to the military population, in several places we have removed “increased significantly” and replaced it with “was significantly higher in 2019 than in 2016.” However, this change results in extra words that do not read as smoothly as “increased.” If the editor or reviewer feels that we need to remove ALL MENTION of “increased” among the military population, we can do so. However, we would argue that the additional language around the differences between surveys in the military population should be sufficient to communicate the desired message.*

**2. Also, more can be done to address the changing nature of the military population. Any additional information regarding the specific characteristics of the military population would be helpful. We know that the vast majority of women are not serving. It would be helpful to know who is serving (husband, son, father, …), length of residence in Kinshasa, duration of service in the military, place of origin (item G2), ethnic background. All these might influence the particular SRH needs of the military population vis a vis the general population.**

*Reply: we have added data in Table 1 and in the text on the following variables for the military population: length of residence in military camps, length of residence in Kinshasa, percent reporting to have been interviewed in 2016, percent born in Kinshasa. We did not have information on the other variables listed above (relationship to the person serving in the military, place of origin, ethnic background).*

**We also see a drastic change in the proportion living with the partners, but that does not necessarily indicate a different population: it might happen that the partner is deployed to a different setting while the woman stays. Information on ongoing conflicts or anything that might sed light would be helpful. It is stated “The same units of residence 162 were used in both military surveys, although the residents could have changed”. You have information on length of stay in the camp (item G4) and in Kinshasa (item G3). Please provide details on this. Maybe a table aside from table 1 looking more in detail at the military population, comparing 2016, 2020, and 2020 separated according to length of stay (4 years of more, or less).**

*Reply: we have added information to Table 1 and in the text on the length of residence in Kinshasa and in the military camps as well as percent born in Kinshasa.*

**3. You can also use this as a covariate to see differences in cp in the military population according to length of stay in Kinshasa, or compare as three different groups those in 2016, those in 2020 that were in 2016 and the newly arrived. The interpretation in terms of success of the military RH campaign is very different. Eg: You might observe a sharp increase in CP of those stayers indicating success of the programs but low CP of new entrants, or the reverse. Those two cases have two very different policy implications. Since this is connected to the main contribution of the paper it needs to be explored.**

*Reply: we have added the suggested comparison of the 3 groups: 2016, 2019-residence in military camps of 4+ years, and 2019-residence of <4 years; see Figure 2.*

**Other minor comments:**

**“Kinshasa, the capital city, has shown the usual effects of urbanization on family size.”: The reader does not need to know what the “usual effects” are. Please describe what you mean, and if it is “usual”, provide a reference.**

*Reply: we have added two citations.*

*White MJ, Muhidin S, Andrzejewski C, Tagoe E, Knight R, Reed H. Urbanization and fertility: an event-history analysis of coastal Ghana. Demography. 2008;45(4):803-16. Epub 2008/12/30. doi: 10.1353/dem.0.0035. PubMed PMID: 19110898; PubMed Central PMCID: PMCPMC2834382.*

*Lerch M. Regional variations in the rural-urban fertility gradient in the global South. PLoS One. 2019;14(7):e0219624. Epub 2019/07/20. doi: 10.1371/journal.pone.0219624. PubMed PMID: 31323039; PubMed Central PMCID: PMCPMC6641161.*

**I would like further consideration of the influence of a prior unplanned pregnancy on use of modern contraception. On line 367 of the mark-up the authors the results section you comment on the higher rate of the most recent pregnancy being unintended among the military population. Then you say "This finding is consistent with the lower levels of knowledge and use among women in the military population." In the discussion section (line 485 of the mark up) you argue that the women in the military population "started from behind" in knowledge and use and the increase in knowledge and use is evidence of this population catching as a result of the family planning programing. If intendedness of the most recent pregnancy is a marker for lower knowledge and use, and I agree with you that it probably is along with use of less effective methods of contraception, then wouldn't this population be the most likely to benefit from family planning programing as they are starting from such low levels of knowledge? Why did you exclude this variable from your analyses?**

*Reply: we introduced the variable “unintended pregnancy” into the analysis of factors affecting MCPR. It was not significant, and it reduced the power because it reduced the number of cases from 2517 to 1873 (omitted were women who had not had a pregnancy in the last five years or were not currently pregnant). We could mention this finding in the text, but our strong preference is to simply omit discussion of unintended pregnancy in this context.*

**2. In the discussion section (Line 530 of the mark up version), you discuss two papers that you are currently writing. I do not think this belongs in your manuscript. I think you can describe these topics as interesting opportunities for future research, but I don't think it is OK to warn off other researchers by describing papers that have not been published.**

*Reply: We have removed the two sentences in question about the other two articles.*

**Please submit your revised manuscript by Jul 10 2021 11:59PM. If you will need more time than this to complete your revisions, please reply to this message or contact the journal office at** [**plosone@plos.org**](mailto:plosone@plos.org)**. When you're ready to submit your revision, log on to** [**https://www.editorialmanager.com/pone/**](https://www.editorialmanager.com/pone/) **and select the 'Submissions Needing Revision' folder to locate your manuscript file.

Please include the following items when submitting your revised manuscript:**

- **A rebuttal letter that responds to each point raised by the academic editor and reviewer(s). You should upload this letter as a separate file labeled 'Response to Reviewers'.**
- **A marked-up copy of your manuscript that highlights changes made to the original version. You should upload this as a separate file labeled 'Revised Manuscript with Track Changes'.**
- **An unmarked version of your revised paper without tracked changes. You should upload this as a separate file labeled 'Manuscript'.**

**If you would like to make changes to your financial disclosure, please include your updated statement in your cover letter. Guidelines for resubmitting your figure files are available below the reviewer comments at the end of this letter.

If applicable, we recommend that you deposit your laboratory protocols in protocols.io to enhance the reproducibility of your results. Protocols.io assigns your protocol its own identifier (DOI) so that it can be cited independently in the future. For instructions see:** [**http://journals.plos.org/plosone/s/submission-guidelines#loc-laboratory-protocols**](https://nam11.safelinks.protection.outlook.com/?url=http%3A%2F%2Fjournals.plos.org%2Fplosone%2Fs%2Fsubmission-guidelines%23loc-laboratory-protocols&data=04%7C01%7Cbertrand%40tulane.edu%7Cde92ad9d79e94014be2d08d920b57091%7C9de9818325d94b139fc34de5489c1f3b%7C1%7C0%7C637576785909657300%7CUnknown%7CTWFpbGZsb3d8eyJWIjoiMC4wLjAwMDAiLCJQIjoiV2luMzIiLCJBTiI6Ik1haWwiLCJXVCI6Mn0%3D%7C1000&sdata=VC4zUyOCkvSG%2FBvkcDsEHfB39ZFVMzYktB6IQjRIF%2F0%3D&reserved=0)**. Additionally, PLOS ONE offers an option for publishing peer-reviewed Lab Protocol articles, which describe protocols hosted on protocols.io. Read more information on sharing protocols at** [**https://plos.org/protocols?utm_medium=editorial-email&utm_source=authorletters&utm_campaign=protocols**](https://nam11.safelinks.protection.outlook.com/?url=https%3A%2F%2Fplos.org%2Fprotocols%3Futm_medium%3Deditorial-email%26utm_source%3Dauthorletters%26utm_campaign%3Dprotocols&data=04%7C01%7Cbertrand%40tulane.edu%7Cde92ad9d79e94014be2d08d920b57091%7C9de9818325d94b139fc34de5489c1f3b%7C1%7C0%7C637576785909667296%7CUnknown%7CTWFpbGZsb3d8eyJWIjoiMC4wLjAwMDAiLCJQIjoiV2luMzIiLCJBTiI6Ik1haWwiLCJXVCI6Mn0%3D%7C1000&sdata=peJO83DKWZNxZ2ZmsK3nfGxvHUECVhuiAkCau5RLcLg%3D&reserved=0)**.**

*Reply: laboratory protocols are not relevant to this paper.*  **We look forward to receiving your revised manuscript.**
